# New Pyrimidinone Bearing Aminomethylenes and Schiff Bases as Potent Antioxidant, Antibacterial, SARS-CoV-2, and COVID-19 Main Protease M^Pro^ Inhibitors: Design, Synthesis, Bioactivities, and Computational Studies

**DOI:** 10.1021/acsomega.3c09393

**Published:** 2024-06-04

**Authors:** Muhammad Sarfraz, Muhammad Ayyaz, Abdul Rauf, Asma Yaqoob, Tooba, Muhammad Arif Ali, Sabir Ali Siddique, Ashfaq Mahmood Qureshi, Muhammad Hassan Sarfraz, Reem M. Aljowaie, Saeedah Musaed Almutairi, Muhammad Arshad

**Affiliations:** † Institute of Chemistry, The Islamia University of Bahawalpur, Bahawalpur 63100, Pakistan; ‡ Institute of Biochemistry, Biotechnology, and Bioinformatics. Department of Biochemistry and Molecular Biology, The Islamia University of Bahawalpur, Bahawalpur 63100, Pakistan; § Department of Chemistry, Government Sadiq College Women University, Bahawalpur 63100, Pakistan; ∥ Nuffield Department of Orthopaedics, Rheumatology and Musculoskeletal Sciences, Botnar Institute of Musculoskeletal Sciences, University of Oxford, OxfordOX3 7LD, United Kingdom; ⊥ Department of Botany and Microbiology, College of Science, King Saud University, P O 2455 Riyadh 11451, Saudi Arabia

## Abstract

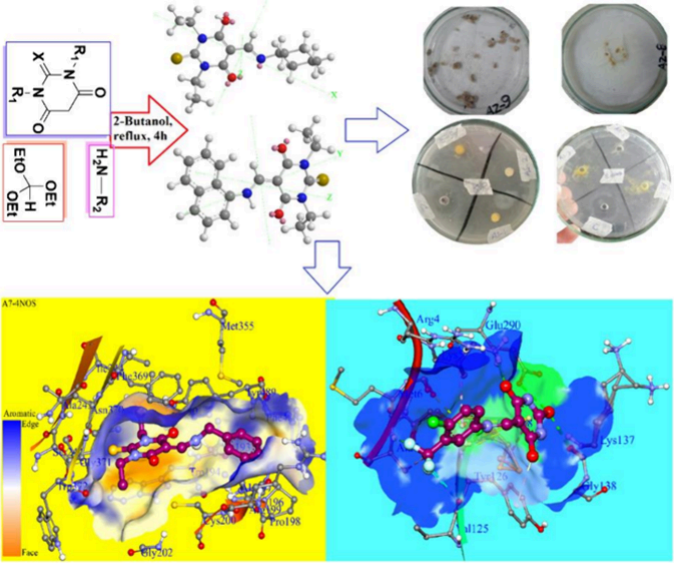

New 2-thioxopyrimidinone derivatives (**A1**–**A10**) were synthesized in 87–96% yields via a simple three-component condensation reaction. These compounds were screened extensively through *in vitro* assays for antioxidant and antibacterial investigations. The DPPH assays resulted in the excellent potency of **A6**–**A10** as antioxidants with IC_50_ values of 0.83 ± 0.125, 0.90 ± 0.77, 0.36 ± 0.063, 1.4 ± 0.07, and 1.18 ± 0.06 mg/mL, which were much better than 1.79 ± 0.045 mg/mL for the reference ascorbic acid. These compounds exhibited better antibacterial potency against *Klebsiella* with IC_50_ values of 2 ± 7, 1.32 ± 8.9, 1.19 ± 11, 1.1 ± 12, and 1.16 ± 11 mg/mL for **A6**–**A10**. High-throughput screenings (HTS) of these motifs were carried out including investigation of drug-like behaviors, physiochemical property evaluation, and structure-related studies involving DFT and metabolic transformation trends. The radical scavenging ability of the synthesized motifs was validated through molecular docking studies through ligand–protein binding against human inducible nitric oxide synthase (HINOS) PDB ID: 4NOS, and the results were promising. Furthermore, the antiviral capability of the compounds was examined by *in silico* studies using two viral proteins PDB ID: 6Y84 and PDB ID: 6LU7. Binding poses of ligands were discussed, and amino acids in the protein binding pockets were investigated, where the tested compounds showed much better binding affinities than the standard inhibitors, proving to be suitable leads for antiviral drug discovery. The stabilities of the molecular docked complexes in real systems were validated by molecular dynamics simulations.

## 1 Introduction

**Figure 1:**
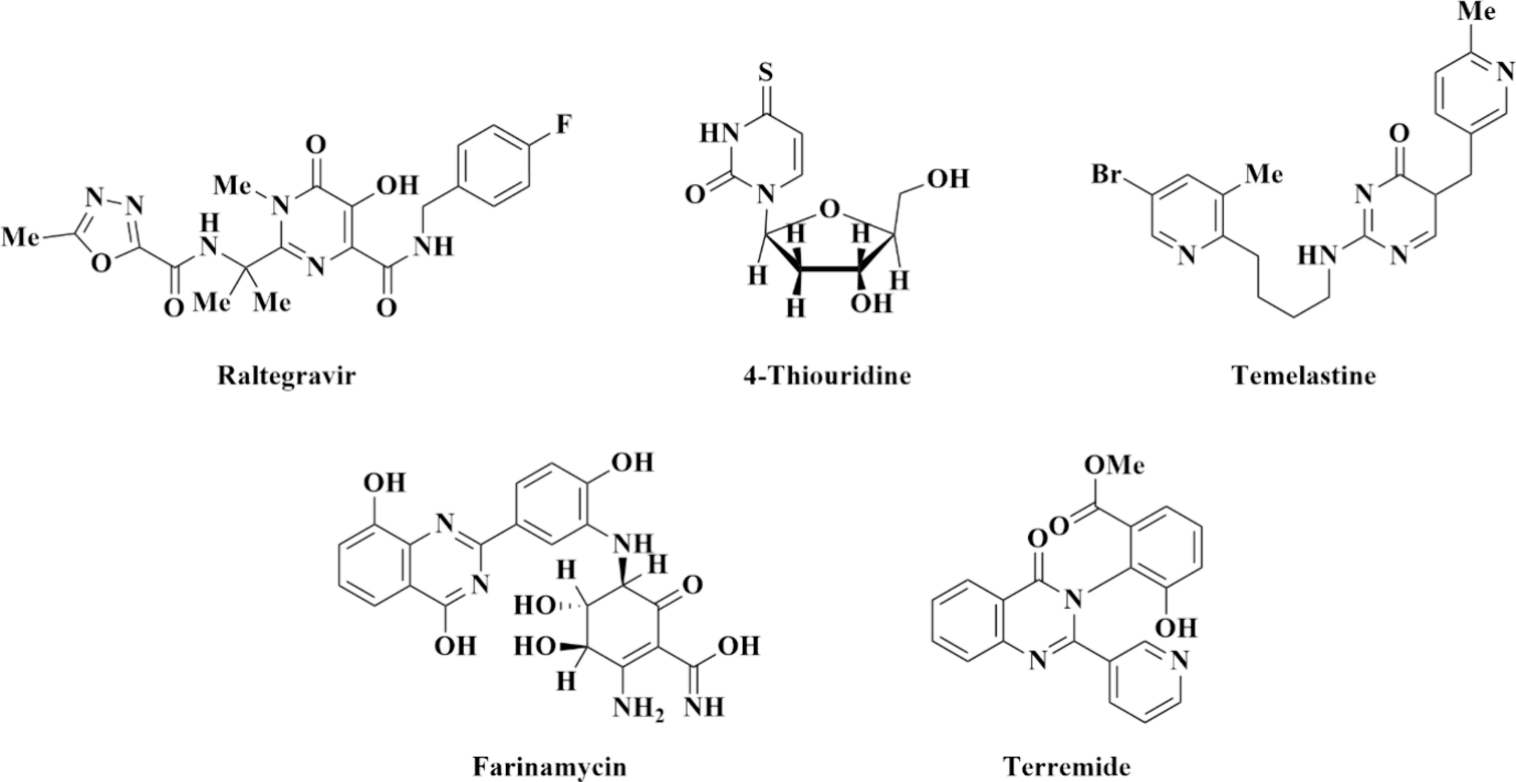
Structures of bioactive natural products and drugs.

Pyrimidines are a subclass of nitrogen-containing heterocycles that have immense importance in medicinal chemistry and drug discovery.^1^ They serve as the core structure in several biologically active compounds,^2^ including antiviral,^3^ anticancer,^4^ and antimicrobial agents.^5^ Additionally, the presence of these heterocyclic fragments modifies the solubility, polarity, lipophilicity, and hydrogen bonding capacity, among other physiochemical and pharmacokinetic features, improving the ADMET qualities of biological molecules making them potential therapeutic candidates as compared to the analogues lacking such features.^6,7^ Furthermore, in drug design, the pyrimidinone moiety is essential because it affects the pharmacological properties of different molecules.^8^ Its adaptability aids in the creation of medications for a variety of therapeutic categories. Medicinal chemistry and the development of novel pharmacological treatments depend on our ability to comprehend and utilize the characteristics of the pyrimidinone scaffold.^9,10^ The structures of some drugs and natural products represented in Figure 1 elaborate the significance of the pyrimidinone moiety in the field of drug discovery. Furthermore, computational chemistry has proven very helpful in creating and predicting the properties of novel pyrimidinone derivatives.^11,12^

The viral disease that has been a matter of interest in the near past, the SARS-CoV-2 disease,^13^ is a contagious disease owing to coronavirus, due to which a great pandemic situation occurred worldwide.^14^ These coronaviruses are a cluster of RNA viruses, which may cause different or similar diseases in various species.^15^ In this regard, various new *in silico* and *in vitro* studies have been conducted to remedy this problem^16−18^ and to approach the lead compounds against SARS-CoV protein substrates.

Moreover, pyrimidines may act as effective electron donors, thus neutralizing harmful free radicals and preventing oxidative stress-induced damage.^19,20^ While further research is needed, pyrimidines hold promise as a valuable agent in combating oxidative stress-related diseases and promoting overall cellular health.^21^ Moreover, NO scavenging is crucial for maintaining proper physiological balance; NOS enzymes play a key role in NO production, and their dysregulation can contribute to various diseases.^22,23^ Effective scavenging of NO helps regulate its levels and prevent associated pathological conditions.^24^ The DPPH antioxidant potential of pyrimidines has also been significantly explored.^25,26^ Through their structural features and electron-donating abilities, pyrimidines can effectively neutralize DPPH radicals,^27^ highlighting their promising role as antioxidant agents with potential applications in health and medicine.^28^ Furthermore, in contemporary research, the biological evaluation of pyrimidines holds immense significance in drug discovery.^29^ Pyrimidines exhibit diverse pharmacological activities, making them pivotal candidates for development of novel therapeutics.^30^ Researchers employ advanced biological assays to assess the potential of pyrimidines in modulating biological targets, including enzymes and receptors.^31^ These evaluations encompass studies on cytotoxicity,^32^ anti-inflammatory properties,^33^ and interaction with specific molecular pathways.^34^ The versatility of pyrimidines, coupled with their proven biological activities, underscores their role as a valuable scaffold in the ongoing quest for innovative drug candidates with enhanced efficacy and reduced side effects.^35^ The structures of free radical scavenging molecules bearing a pyrimidinone moiety are shown in Supplementary Data, Figure S2.

Keeping in view the above facts, we employed three target substrates, i.e., SARS-CoV-2 main protease enzyme, COVID-19 main protease M^Pro^, and HINOS enzyme, to dock newly synthesized pyrimidinone derivatives **A1**–**A10** along with some reference compounds including a naturally bound inhibitor N3 with the COVID enzymes, remdesivir, hydroxychloroquine, chloroquine, and ronoptrin. Ligand-protein tying affinities and ligand efficiency values of scrutinized motifs **A1**–**A10** against their target substrates were compared with each other and with the reference compounds.

## 2 Materials and Methods

### 2.1 Chemical Synthesis

#### 2.1.1 Experimental Methodology Details

Reagent-grade chemicals and solvents were bought from Sigma-Aldrich/Merck, and precoated aluminum plates for thin layer chromatography (TLC) were obtained from Merck silica gel F254 followed by visualization of developed chromatograms under two wavelengths 254 and 360 nm. The melting points of the synthesized compounds were determined by using a Gallenkamp (Griffin) apparatus. Infrared (IR) spectra were acquired using a Nicolet AVATAR 360 FTIR spectrometer equipped with a Smart OMNI-Sampler and analyzed in terms of wavenumbers (cm^–1^). A Micromass Platform LCZ spectrometer using the electrospray ionization (ESI) technique was employed to record low-resolution mass spectra (LRMS) in methanol. High-resolution mass spectra (HRMS) were recorded with a Waters QTof Xevo spectrometer or Fisons/VG AutoSpec TOF spectrometer at 70 eV with the ESI technique. Proton (^1^H) and carbon (^13^C) nuclear magnetic resonance (NMR) spectra were recorded at a Varian INOVA 500 MHz spectrometer with 500 and 125 MHz frequencies or a Varian Mercury 400 MHz spectrometer with 400 and 100 MHz frequencies, respectively. 1D and 2D NMR spectra, attached proton test (APT), gCOSY, gHSQC, and gHMBC were used to assign proton and carbon atoms of all of the synthesized compounds.

#### 2.1.2 General Procedure for Synthesis

**Scheme 1:**
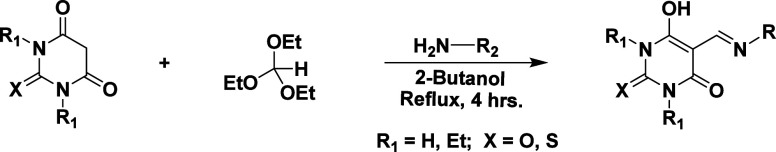
Reaction Scheme for the Single-Step Condensation of 1,3-Diethyl-2-thioxodihydropyrimidine-4,6(1*H*,5*H*)-dione, triethyl orthoformate, and Aromatic Amines

In a 100 mL round-bottom flask, 0.3 g (1.5 mmol) of 1,3-diethyl-2-sulfanylidene-1,3-diazinane-4,6-dione/1,3-diazinane-2,4,6-trione was taken followed by the addition of 2-butanol (20 mL). To make the solution, the prepared suspension was heated for 20 min after being sonicated for 15 min at 50 °C. 0.3 mL (1.8 mmol) of triethyl orthoformate was added to the sonicated mixture followed by the addition of 1.5 mmol of the respective aromatic or alicyclic amines (cyclohexanamine, 4-(2-ethylamino)benzene-1-sulfonamide, 3-aminobenzene-1-sulfonamide, 3-aminobenzonitrile, and 4-chloro-3-(trifluoromethyl)aniline), and the mixture was refluxed for 4–5 h (Scheme 1). Following this, the contents in the flask were rendered to stand for 5 min at ambient temperature for the precipitation or crystallization of the product, which was followed by filtering using gentle suction. A few milliliters of EtOAc was then added to fully wash out 2-butanol into the filtrate. For spectrometric analyses, this solid residue was further cleaned by being washed three times with acetone.

##### (*E*)-5-((Cyclohexylimino)methyl)-1,3-diethyl-6-hydroxy-2-thioxo-2,3-dihydropyrimidin-4(1*H*)-one (**A1**)

Off-white powder. Yield 88%; mp 171–173 ^ο^C; IR (neat, cm^–1^) υ_max_ 2977 (w), 2931 (m), 2858 (w), 1646 (s), 1577 (s), 1384 (s), 1292 (s), 1101 (s), 792 (m); ^1^H NMR (*d6*-DMSO, ppm) δ 7.74 (1H, br.s, −O***H***), 6.71 (1H, s, −N=C***H***−), 4.31–4.36 (4H, q, *J* 6.8, −C***H*_2_**–CH_3_), 2.93–2.99 (1H, m, −N–C***H***<), 1.86–1.88 (2H, m, eq 2-CH-C***H***-CH_2_), 1.67–1.73 (2H, m, eq 2-CH_2_–C***H*_2_**-CH_2_), 1.56–1.59 (1H, m, eq −CH_2_–C***H*_2_**-CH_2_−), 1.18–1.25 (4H, m, C2 and C3 ax. ring ***H***), 1.07–1.11 (7H, m, 2-CH_2_–C***H*_3_** and ax. −CH_2_–C***H*_2_**–CH_2_−); ^13^C NMR (*d6*-DMSO, ppm) δ 175.5 (**C**=S), 161.4 (3**C**), 146.1 (**C**H), 49.3 (**C**H), 40.9 (2**C**H_2_), 30.4 (2**C**H_2_), 24.5 (**C**H_2_), 23.7 (2**C**H_2_), 12.7 (2**C**H_3_); MS (EI) *m*/*z* 308 (M-H 95%), 249 (70), 212 (90), 158 (55), 99 (80); HRMS (ESI) [M – H]^−^, found 309.43 for C_15_H_23_N_3_O_2_S.

##### (*E*)-4-(2-(((1,3-Diethyl-6-hydroxy-4-oxo-2-thioxo-1,2,3,4-tetrahydropyrimidin-5-yl)methyl-ene)amino)ethyl)benzenesulfonamide (**A2**)

Off-white powder. Yield 91%; mp 209–211 °C. IR (neat, cm^–1^) υ_max_ 3305 (w), 3222 (w), 2983 (w), 1676 (m), 1638 (s), 1598 (m), 1472 (m), 1457 (m), 1393 (m), 1382 (m), 1328 (s), 1156 (m); ^1^H NMR (*d6*-DMSO, ppm) δ 10.65 (1H, br.s, −OH), 8.32 (1H, s, −N=C***H***−), 7.76–7.77 (2H, d, *J* =8.0, Ar***H***), 7.44–7.46 (2H, d, *J* = 8.5, Ar***H***), 7.31 (2H, br.s, −SO_2_N***H*_2_**), 4.36–4.42 (4H, m, *J* 6.5, −C***H*_2_**–CH_3_), 3.79–3.82 (2H, t, *J* 7.0, −N–C***H*_2_**–CH_2_−), 3.01–3.04 (2H, t, *J* = 7.0, −N–CH_2_–C***H*_2_**−), 1.13–1.19 (6H, m, (C***H*_3_**); ^13^C NMR (*d6*-DMSO, ppm) δ 178.1 (**C**=S), 161.4 (**C**=O), 160.6 (**C**H), 160.2 (**C**), 142.4 (**C**), 142.2 (**C**), 129.3 (2**C**H), 125.8 (2**C**H), 91.4 (**C**), 51.0 (**C**H_2_), 42.1 (**C**H_2_), 41.5 (**C**H_2_), 35.6 (**C**H_2_), 12.3 (**C**H_3_), 12.2 (**C**H_3_); MS (EI) *m*/*z* 409 (M-H 35%), 353 (10), 329 (16), 269 (23), 199 (100); HRMS (ESI) [M – H]^−^, found 409.1012 for C_17_H_21_N_4_O_4_S_2_.

##### (*E*)-3-(((1,3-Diethyl-6-hydroxy-4-oxo-2-thioxo-1,2,3,4-tetrahydropyrimidin-5-yl)methyl-ene)amino)benzenesulfonamide (**A3**)

Greenish-yellow solid. Yield 96%; mp 197–199 °C; IR (neat, cm^–1^) υ_max_ 3312 (w), 3220 (w), 3093 (w), 2963 (w), 1679 (w), 1635 (w), 1592 (m), 1585 (m), 1280 (s), 1080 (s), 1015 (m), 795 (s); ^1^H NMR (*d6*-DMSO, ppm) δ 12.25 (1H, s, −O***H***), 8.71 (1H, s, −N=C***H***−), 8.04 (1H, s, Ar***H***), 7.85 (1H, d, *J* 8.5, Ar***H***), 7.71 (1H, d, *J* 7.5, Ar***H***), 7.64 (1H, t, *J* 7.5, Ar***H***), 7.47 (2H, br.s, −SO_2_N***H*_2_**), 4.45 (4H, q, *J* 6.5, -C***H*_2_**-CH_3_), 1.22 (6H, t, *J* 6.5, −CH_2_–C***H*_3_**); ^13^C NMR (*d6*-DMSO, ppm) δ 178.4 (**C**=S), 160.0 (**C**), 157.5 (**C**=O), 153.9 (**C**H), 145.5 (**C**), 139.0 (**C**), 130.4 (**C**H), 123.2 (**C**H), 123.0 (**C**H), 116.0 (**C**H), 94.7 (**C**), 42.5 (**C**H_2_), 41.9 (**C**H_2_), 12.2 (2**C**H_3_); MS (EI) *m*/*z* 381 (M-H 100%), 293 (7), 364 (40), 273 (2), 227 (4), 159 (2); HRMS (ESI) [M – H]^−^, found 381.0705 for C_15_H_17_N_4_O_4_S_2_.

##### (*E*)-3-(((6-Hydroxy-2,4-dioxo-1,2,3,4-tetrahydropyrimidin-5-yl)methylene)amino)benzonitrile (**A4**)

Light-yellow powder. Yield 91%; mp 176–178 °C; IR (neat, cm^–1^) υ_max_ 3582 (w), 3229 (m), 3214 (w), 3202 (w), 3169 (w), 3120 (w), 2818 (w), 2238 (w), 1767 (m), 1651 (s), 1617 (m), 1589 (s), 1345 (s), 1322 (s), 525 (s), 497 (s); ^1^H NMR (*d6*-DMSO, ppm) δ 11.85 (1H, br.s,-O***H***),10.96 (2H, br.s, 2 > N***H***), 8.59 (1H, s, −N=C***H***−), 8.13 (1H, s, Ar***H***), 7.86 (1H, d, *J* 7.2, Ar***H***), 7.67 (1H, d, *J* 7.6, Ar***H***), 7.60 (1H, t, *J* 8.0, Ar***H***); ^13^C NMR (*d6*-DMSO, ppm) δ 151.3 (**C**), 151.2 (**C**H), 150.0 (2**C**=O), 139.3 (**C**), 130.4 (**C**H), 128.6 (**C**H), 123.0 (**C**H), 121.7 (**C**H), 117.5 (**C**), 112.3 (**C**), 93.4 (**C**); MS (EI) *m*/*z* 255 (M-H 100%), 213 (9), 169 (1), 141 (5); HRMS (ESI) [M – H]^−^, found 255.0524 for C_12_H_7_N_4_O_3_.

##### (*E*)-5-(((4-Chloro-3-(trifluoromethyl)phenyl)imino)methyl)-6-hydroxypyrimidine-2,4(1*H*,3*H*)-dione (**A5**)

Light-yellow powder. Yield 87%; mp 188–190 °C; IR (neat, cm^–1^) υ_max_ 3192 (m), 3069 (w), 1704 (s), 1683 (s), 1347 (s), 1157 (s), 1034 (m), 846 (m), 526 (s), 440 (s); ^1^H NMR (*d6*-DMSO, ppm) δ 11.86 (1H, br.s, −OH), 10.94 (2H, br.s, 2 > NH), 8.57 (1H, s, −N=C***H***−), 8.10 (1H, d, *J* 2.4, Ar***H***), 7.86–7.88 (1H, dd, *J* 8.8, 2.8, Ar***H***), 7.73–7.75 (1H, d, *J* 8.8, Ar***H***); ^13^C NMR (*d6*-DMSO, ppm) δ 165.7 (**C**=O), 164.1 (**C**=O), 151.9 (**C**H), 150.6 (**C**), 138.4 (**C**), 132.6 (**C**H), 127.03–127.78 (**C**, q, *J* 31.25), 126.8 (**C**), 123.8 (**C**H), 119.25–125.78 (**C**F_3_, q, *J* 272.5), 118.9–119.05 (**C**H), 93.7 (**C**); MS (EI) *m*/*z* 255 (M – H 100%), 213 (9), 169 (1), 141 (5); HRMS (ESI) [M – H]^−^, found 332.0062 for C_12_H_6_N_3_O_3_F_3_Cl.

##### 1,3-Diethyl-5-{[(naphthalen-1-yl)amino]methylidene}-2-sulfanylidenedihydropyrimidine-4,6(1*H*,5*H*)-dione (**A6**)

Yellow powder. Yield 82%; IR (neat, cm^–1^) υ_max_ 3056 (w), 2980 (w), 2931 (w), 1672 (s), 1628 (s), 1606 (s), 1595 (s), 1455 (s), 1407 (s), 1381 (s), 1301 (s), 1278 (s), 1081 (s), 840 (m), 466 (m); ^1^H NMR (CDCl_3_, 300 MHz) δ 13.12 (1H, d, *J* 12.9, =CH–N***H***−), 8.87 (1 H, d, *J* 13.5, =C***H***–NH−), 8.08 (1H, d, *J* 8.4, Ar***H***), 7.94 (1H, d, *J* 8.1 Ar***H***), 7.80–7.86 (1H, m, Ar***H***), 7.51–7.71 (4H, m, Ar***H***), 4.61 (4H, q, *J* 7.0, −C***H*_2_**–CH_3_), 1.30–1.40 (6H, m, −CH_2_–C***H*_3_**); ^13^C NMR (CDCl_3_, 75 MHz) δ 178.9 (**C**=S), 163.5 (**C**=O), 160.8 (**C**=O), 154.5 (**C**H), 134.3 (**C**), 134.2 (**C**), 128.8 (**C**H), 127.7 (**C**H), 127.6 (**C**H), 127.2 (**C**H), 125.7 (**C**H), 125.4 (**C**), 120.3 (**C**H), 114.8 (**C**H), 95.6 (**C**), 43.1 (**C**H_2_), 42.5 (**C**H_2_), 12.5 (**C**H_3_), 12.3 (**C**H_3_); HRMS (ESI) [M + H]^+^, found 354.12724 for C_19_H_20_N_3_O_2_S.

##### 5-[(Benzylamino)methylidene]-1,3-diethyl-2-sulfanylidenedihydropyrimidine-4,6(1*H*,5*H*)-dione (**A7**)

White crystals. Yield 86%; IR (neat, cm^–1^) υ_max_ 3056 (w), 2968 (w), 2936 (w), 1665 (m), 1635 (m), 1590 (m), 1485 (s), 1440 (s), 1367 (m), 1226 (s), 1112 (m), 686 (m), 480 (m); ^1^H NMR (d6-DMSO, 300 MHz) δ 10.56 (1H, s, =CH–N***H***−), 8.15 (1 H, s, =C***H***–NH−), 7.36–7.48 (5H, m, Ar***H***), 4.33 (4H, q, *J* 6.9, 2 −C***H*_2_**–CH_3_), 4.04 (2H, s, −C***H*_2_**–Ar), 1.09 (6H, t, *J* 6.9, 3 −CH_2_–C***H*_3_**); ^13^C NMR (d6-DMSO, 75 MHz) δ 175.4 (**C**=S), 165.7 (**C**=O), 164.8 (**C**=O), 161.3 (**C**H), 134.0 (**C**), 128.8 (2**C**H), 128.7 (2**C**H), 128.5 (**C**H), 80.0 (**C**), 42.3 (**C**H_2_), 41.0 (2**C**H_2_), 12.8 (2**C**H_3_); HRMS (ESI) [M – H]^−^, found 316.18454 for C_16_H_18_N_3_O_2_S.

##### 1,3-Diethyl-5-{[(2-phenylethyl)amino]methylidene}-2-sulfanylidenedihydropyrimidine-4,6(1*H*,5*H*)-dione (**A8**)

White crystals. Yield 86%; IR (neat, cm^–1^) υ_max_ 3026 (w), 2978 (w), 2930 (w), 1675 (m), 1635 (m), 1597 (m), 1495 (s), 1450 (s), 1377 (m), 1216 (s), 1102 (m), 696 (m), 489 (m); ^1^H NMR (d6-DMSO, 300 MHz) δ 10.62 (1H, s, =CH–N***H***-), 8.23 (1 H, s, =C***H***–NH−), 7.22–7.38 (5H, m, Ar***H***), 4.38 (4H, m, 2 −C***H*_2_**–CH_3_), 3.78 (2H, t, *J* 7.3, −C***H*_2_**–NH−), 2.94 (2H, t, *J* 7.3, −C***H*_2_**–Ar), 1.20–1.12 (6H, m, 2 −C***H*_3_**); ^13^C NMR (d6-DMSO, 75 MHz) δ 178.2 (**C**=S), 161.4 (**C**=O), 160.6 (CH), 160.2 (C=O), 138.0 (**C**), 128.9 (2**C**H), 128.5 (2**C**H), 126.6 (**C**H), 91.3 (**C**), 51.4 (**C**H_2_), 42.1 (**C**H_2_), 41.5 (**C**H_2_), 35.9 (**C**H_2_), 12.3 (2**C**H_3_); HRMS (ESI) [M – H]^−^, found 330.12815 for C_17_H_20_N_3_O_2_S.

##### 5-{[([1,1′-Biphenyl]-2-yl)amino]methylidene}-1,3-diethyl-2-sulfanylidenedihydropyrimid-ine-4,6(1*H*,5*H*)-dione (**A9**)

Light-yellow crystals. Yield 84%; IR (neat, cm^–1^) υ_max_ 3261 (w), 3081 (w), 3028 (w), 2989 (w), 2973 (w), 2926 (w), 2868 (w), 1670 (m), 1610 (s), 1579 (s), 1454 (s), 1426 (s), 1371 (s), 1303 (s), 1280 (s), 1234 (s), 1099 (s), 841 (m), 487 (s); ^1^H NMR (d6-DMSO, 500 MHz) δ 12.20 (1H, d, *J* 10.1, =CH–N***H***−), 8.70 (1 H, d, *J* 10.9, =C***H***–NH−), 7.80 (1H, d, *J* 8.1, Ar***H***), 7.41–7.52 (8H, m, Ar***H***), 4.35 (4H, dd, *J* 6.3, 2 −C***H*_2_**), 1.10–1.23 (6H, m, 3 −C***H*_3_**); ^13^C NMR (d6-DMSO, 125 MHz) δ 178.2 (**C**=S), 161.9 (**C**=O), 159.8 (**C**=O), 154.5 (**C**H), 136.5 (**C**), 133.3 (**C**), 131.0 (**C**H), 129.4 (**C**), 129.3 (**C**H), 129.2 (2**C**H), 129.1 (2**C**H), 128.4 (**C**H), 127.1 (**C**H), 118.8 (**C**H), 94.3 (**C**), 42.3 (**C**H_2_), 41.6 (**C**H_2_), 12.2 (**C**H_3_), 12.1 (**C**H_3_); HRMS (ESI) [M – H]^−^, found 378.1285 for C_21_H_20_N_3_O_2_S.

##### 5-{[([1,1′-Biphenyl]-4-yl)amino]methylidene}-1,3-diethyl-2-sulfanylidenedihydropyrimid-ine-4,6(1*H*,5*H*)-dione (**A10**)

Yellow powder. Yield 85%; IR (neat, cm^–1^) υ_max_ 3344 (w), 3058 (w), 3030 (w), 2978 (w), 2933 (w), 2871 (w), 1678 (m), 1615 (s), 1580 (s), 1465 (s), 1433 (s), 1372 (s), 1302 (s), 1280 (s), 1239 (s), 1079 (s), 832 (m), 473 (s); ^1^H NMR (d6-DMSO, 500 MHz) δ 12.24 (1H, d, *J* 14.3, =CH–N***H***−), 8.73 (1 H, d, *J* 14.3, =C***H***–NH−), 7.35–7.77 (9H, m, Ar***H***), 4.46 (2H, q, *J* 6.9, −C***H*_2_**), 4.44 (2H, q, *J* 6.9, −C***H*_2_**), 1.24 (3H, t, *J* 6.9, −C***H*_3_**), 1.21 (3H, t, *J* 6.9, −C***H*_3_**); ^13^C NMR (d6-DMSO, 125 MHz) δ 178.2 (**C**=S), 161.7 (**C**=O), 160.0 (**C**=O), 153.4 (**C**H), 138.9 (**C**), 138.3 (**C**), 137.7 (**C**), 129.0 (2**C**H), 127.8 (2**C**H), 126.5 (2**C**H), 119.7 (2**C**H), 114.4 (**C**H), 94.2 (**C**), 42.3 (**C**H_2_), 41.8 (**C**H_2_), 12.3 (**C**H_3_), 12.2 (**C**H_3_); HRMS (ESI) [M + H]^+^, found 380.1433 for C_21_H_22_N_3_O_2_S.

### 2.2 DPPH (1,1-Diphenyl-2-picryl-hydrazyl) Free Radical Scavenging Methodology

Antioxidant properties of the synthesized compounds were evaluated by employing DPPH free radical scavenging assays with slight modifications. A mixture made up of 900 mL of DDPH solution (varying from 100 to 1000 g) and 100 mL of the test substance solubilized in DMSO was created and then kept at 37 °C for 1 h in a light-protected condition. Analyses were carried out thrice for each test compound, and light absorbance at 517 nm was recorded by UV-3000 voltage 100–240 V50/60 Hz. The percent inhibition of DPPH activity was determined using the following formula.
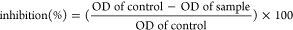
1

The IC_50_ values were determined by repeating the assays with appropriately diluted test solutions. To compute the IC_50_ values for the active compounds, the data was analyzed by GraphPad Prism software and the results were manifested as mean ± SEM, *n* = 3.

### 2.3 Antibacterial Assay

The bacterial strains were cultured in the broth for 8 h at 37 °C. The level of antibiotic activity for the test materials was determined using the disc diffusion technique for antimicrobial sensitivity evaluation. Some bacterial cultures were used to spread the microbes evenly on the agar plates with the help of a sterile swab. The discs that had been coated with several of the test samples were put on the agar surface after the dishes had dried for 15 min. The standard antibiotic disc was 10 μg for *Klebsiella pneumoniae* and *Escherichia coli*, and the negative control was solvent without the sample. Based on the type of bacteria employed in the experiment, the plate was then incubated at 37 °C for 18 to 24 h and the plates were tested for an inhibitory zone. To ensure the reliability of these experimental protocols, the tests were conducted thrice. Determination of minimum inhibition concentrations (MICs) was carried out using the ICD method.^36^ The MIC value for each bacterial strain was determined by noting the smallest dosage that prevents bacterial growth.

### 2.4 Computational Details

All quantum mechanical calculations of the synthesized compounds **A1**–**A10** were conducted using the Gaussian 16 program package.^37^ The optimized geometries were sketched with GaussView 6.1.1.^38^ The NMR estimates were carried out using the GIAO formalism at the B3LYP/6-31G(d) level of theory.^39^ The chemical shifts of compounds **A1**–**A10** were computed with reference to TMS. The Multiwfn software^40^ and VMD software were utilized for visualizing and drawing the geometries^41^ as well as performing frontier molecular orbital analysis.

### 2.5 Medicinal Chemistry, Docking, and MD Simulation Protocols

The chemical structures were drawn using ChemDraw Professional 19.1.0.8. Energy minimizations were performed using PyRx-Python Prescription 0.8 software (https://pyrx.sourceforge.io/) for optimization purposes. Various tools including molinspiration (https://www.molinspiration.com/cgi-bin/properties), molsoft (http://www.molsoft.com/mprop/), eADMET (http://www.eadmet.com/en/physprop.php),^42^ OSIRIS Property Explorer Online, and DataWarrior 5.0.0 software were utilized to assess the physicochemical properties, toxicities, drug-like characteristics, and ADMET profiles. Some of the physiochemical properties and the medicinal chemistry of the test compounds were estimated by the application of the ADMETlab 2.0 server (https://admetmesh.scbdd.com/service/evaluation/index) and RO5^43^ was validated. Validation of the BOILED-Egg model, bioavailability score, and lead likeness was measured through the SwissADME server (http://www.SwissADME.ch/index.php). Moreover, the AutoDockTools 1.5.6 program^44^ was used to determine ligand-protein binding complexes with least binding powers and ligand efficacies of small molecules against the target substrates. The AutoDockTools 1.5.6 software, utilizing the Lamarckian genetic algorithm (LGA) with a number of GA runs of 50, was employed for the docking simulations. The grid box parameters were optimized for each specific substrate, considering the appropriate spacing values. The best docked energy complexes were further analyzed and visualized through BIOVIA Discovery Studio software.^45^ A pre-compiled binary program, NAMD 2.14 software,^46^ acquired from http://www.ks.uiuc.edu/Research/namd/, was employed for MD simulation analyses of ligand–substrate complexes followed by simulation setup, trajectory analysis, visualization, and further analyses of simulated ligand–substrate complexes using VMD 1.9.3.^47^ CGenFF^48,49^ with the CHARMM GUI-based ligand reader and modeler was used to generate ligand topology.^50,51^

## 3 Results and Discussion

### 3.1 Candidate Ligands

**Figure 2:**
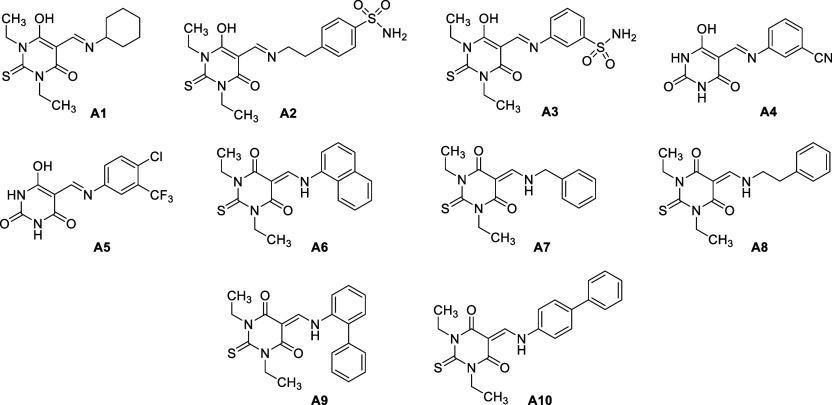
Structures of newly synthesized Schiff bases as candidate ligands **A1**–**A5** and aminomethylene-based thiobarbiturates **A6**–**A10**.

The medicinal importance of Schiff bases^52^ prompted us to work on the development of the aminomethylene moiety, which might be applicable to the synthesis of Schiff bases **A1**–**A5** through a one-step condensation reaction of 1,3-dithyl-2-thiobarbituric/barbituric acid, triethyl orthoformate, and aromatic or alicyclic amines. The structures of compounds **A1**–**A10** are shown in Figure 2. The compounds **A6**–**A10** were also synthesized by the same methodology, but the results were unexpectedly different and only the aminomethylenes were predominantly obtained; also, both sets of compounds were distinguished by a singlet (**A1**–**A5**) or doublet (**A6**–**A10**) appearing in the region of 8.00–9.00 ppm in the ^1^H NMR spectrum.

#### 3.1.1 Spectral Characterization

NMR, IR, and MS techniques were employed to characterize the chemical structures (Figures S4–S39 in the Supplementary Data). NMR data reveal that the spectra of all the compounds represent a singlet signal in the range of 8.32–8.71 ppm for the −CH=N– group, except compound **A1** that showed a singlet signal for one proton at 4.28 ppm attributing the proton of the −CH=N– group, suggesting the formation of an imine linkage. Further evidence of this linkage is attributed from the two-bond HMBC weak correlation of this proton with *C4*, *C5*, and *C6* of the pyrimidinone ring as well as the three-bond HMBC strong correlation with the iminium *N*-substituted carbon atom of the aromatic ring in compounds **A3**–**A5**. Another evidence for −CH=N– linkage is attributed in compound **A2** where two protons of the iminium *N*-substituted carbon of the ethyl moiety show a three-bond HMBC strong correlation with the carbon atom of the −CH=N– linkage appearing at 160.63 ppm. In addition, those two protons of compound **A2**, which are mentioned above, also exhibit a strong three-bond HMBC correlation with the quaternary carbon atom of the aromatic ring appearing at 142.42. Considering compound **A1**, a broad singlet at 7.74 ppm suggested the attribution of a hydroxyl proton on the pyrimidinone ring. The protons of the CH_2_ group of the ethyl chain substituted at the nitrogen atom of the pyrimidinone ring, appearing at 4.41 and 4.36 ppm as a quartet, show a three-bond HMBC correlation with the carbon atoms of C=S and C=O functionalities. The appearance of one quartet for two CH_2_ and a triplet for two CH_3_ groups appearing at 4.41–4.36 and 1.07–1.11 ppm, respectively, for two ethyl groups substituted at both nitrogen atoms of the pyrimidinone ring exhibits the chemical and magnetic equivalence of these two ethyl groups and hence proves that the *C5* of the pyrimidinone ring is singly bonded to that side chain, which further contains the imine functionality, categorizing the whole compound as a Schiff base. This equivalence of the two ethyl groups is attributed to the free rotation of the single bond between *C5* and the side chain; however, in contrast to this, if there was a double bond instead of a previously discussed single bond, then one ethyl group was *syn* and another *anti* to the nitrogen atom of the side chain substituted to *C5* of the pyrimidinone ring. Furthermore, axial and equatorial protons of the cyclohexyl ring appear differently in the ^1^HNMR spectrum as multiplets being equatorial protons appearing downfield compared to axial protons by approximately 0.5 ppm. Examining compound **A3**, a broad singlet for one proton appearing at 12.25 ppm attributes the presence of hydroxy proton at the pyrimidinone ring; however, a peak appearing at 8.71 as a singlet attributes the presence of the −CH=N– group and four peaks for aromatic protons in the range 7.63–8.04 ppm. It is worth noting that the aromatic proton appearing at 7.63–7.66 ppm, as a triplet shows a three-bond HMBC correlation, and the aromatic proton at 7.84–7.86 ppm, as a doublet of doublet of doublet shows a two-bond HMBC correlation with the quaternary carbon at 139.03 ppm similar to the carbon showing an HMBC correlation with proton of the −CH=N– group appearing at 8.71 ppm. Furthermore, a broad singlet signal appearing at 7.47 ppm, integrating for two protons, attributes the presence of −SONH_2_ groups at the aromatic ring. The two ethyl groups, one at each of the nitrogen atom of the pyrimidinone ring, produce only one quartet at 4.43–4.47 ppm, integrating for four protons, and a triplet 1.20–1.23 ppm, integrating for six protons, showing that both ethyl groups are chemically and magnetically equivalent. Also, CH_2_ groups of these ethyl chains show HMBC correlations with carbon atoms of C=S and C=O functionalities, appearing at 178.35 and 160.00 ppm, respectively, along with the two-bond HMBC correlation with the carbon atoms of adjacent CH_3_ groups, appearing at 12.19 ppm.

### 3.2 DPPH (1,1-Diphenyl-2-picryl-hydrazyl) Free Radical Scavenging Assay

The DPPH activity results were obtained for five compounds (**A6**–**A10**) along with a standard reference, ascorbic acid. These results offer an understanding of the potency and effectiveness of these compounds in scavenging DPPH radicals, which indicate their potential as antioxidants. Among the tested compounds, **A8** demonstrated the highest potency with an IC_50_ value of 0.36 ± 0.063 mg/mL. This indicates that **A8** has a strong ability to neutralize DPPH radicals at a relatively lower concentration. This finding suggests that **A8** possesses significant antioxidant activity and could potentially serve as an effective free radical scavenger. Compound **A6** showed an IC_50_ value of 0.83 ± 0.125 mg/mL, indicating its ability to effectively scavenge DPPH radicals but at a slightly higher concentration compared to **A8**. Similarly, **A10** displayed an IC_50_ value of 1.18 ± 0.06 mg/mL, indicating a potency slightly lower than that of **A6**. Nonetheless, both compounds demonstrated notable antioxidant activity and the potential to counteract free radicals.

On the other hand, **A7** and **A9** exhibited relatively lower potency in the DPPH assay. **A7** displayed an IC_50_ value of 0.90 ± 0.77 mg/mL, indicating a moderate ability to scavenge DPPH radicals. **A9** had an IC_50_ value of 1.4 ± 0.07 mg/mL, suggesting a comparatively lower potency among the tested compounds. Although these compounds showed relatively weaker antioxidant activity, they still exhibited some level of the DPPH scavenging ability. Comparing the inhibition percentages at a concentration of 0.5 mg, **A8** demonstrated the highest inhibition at 68.75 ± 0.05%. This indicates that **A8** is highly effective at inhibiting the DPPH radicals. **A6** exhibited an inhibition percentage of 30.00 ± 0.136%, while **A10** showed an inhibition percentage of 21.09 ± 0.071%. These values demonstrate their ability to scavenge free radicals, albeit at a slightly lower level compared to **A8**. Compounds **A7** and **A9** displayed lower inhibition percentages at the concentration of 0.5 mg, with values of 25.12 ± 0.008 and 17.53 ± 0.28%, respectively. These results suggest a relatively weaker capacity of these compounds to scavenge DPPH radicals compared to those of the other tested compounds.

When the results are compared to the standard reference, ascorbic acid, it can be observed that **A8** exhibited higher potency in scavenging DPPH radicals. Ascorbic acid, with an IC_50_ value of 1.79 ± 0.045 mg/mL, served as the benchmark antioxidant compound in this study. The superior potency of **A8** indicates its potential as a strong antioxidant compound with a significant free radical scavenging activity. Overall, these findings highlight the varying degrees of potency among the tested compounds in scavenging DPPH radicals. **A8** demonstrated the highest efficacy, followed by **A6** and **A10**, while **A7** and **A9** exhibited relatively lower activity; the results are summarized in Table 1.

**Table 1:** DPPH Free Radical Scavenging Activities of Compounds **A6**–**A10** (IC_50_, mg/mL)

**sr. no.**	**compounds**	**inhibition (%) at 0.5 mg**	**inhibition (%) at 1.0 mg**	**DPPH free radical scavenging activity**^a^**IC**_**50**_(mg/mL)
**1**	**A6**	30.00±0.136	38.46 ± 0.04	0.83 ± 0.125
**2**	**A7**	25.12±0.008	24.55 ± 0.3	0.90 ± 0.77
**3**	**A8**	68.75 ± 0.05	74.769 ± 0.26	0.36 ± 0.063
**4**	**A9**	17.53 ± 0.28	10.76 ± 1.84	1.4 ± 0.07
**5**	**A10**	21.09 ± 0.071	36.3 ± 0.04	1.18 ± 0.06
**6**	ascorbic acid	positive control^b^		1.79 ± 0.045

a Standard error of mean of three assays.

b Positive control used in the assays. Data shown are values from triplicate experiments.

### 3.3 Antibacterial Assays

The literature reports the antibacterial efficacy of pyrimidinones against a variety of bacteria.^53,54^ Therefore, we studied the synthesized motifs **A6**–**A10** and a general antibacterial sensitivity test (inhibition zone, mm) was accomplished on three test organisms (*Klebsiella*, *S. aureus*, and *E. coli*)^55^ for the new 5-substituted pyrimidinone derivatives and the clinical standard ampicillin. Zones of inhibition (ZOIs) were used to estimate the antibacterial potential of these compounds **A6**–**A10** against three microorganisms in an *in vitro* test, and the results are summarized in Table 2. Among the tested compound, **A9** exhibited the greatest activity against *Klebsiella* showing an IC_50_ value of 1.1±12 mg/mL followed by **A10** and **A8** with comparable activities bearing the values of 1.16±11 and 1.19±11 mg/mL, respectively, whereas **A7** was appropriately active against *S. aureus* strain with an IC_50_ value of 1.38±9 mg/mL; the rest of the analogues showed poor activity against *S. aureus*. The tested compounds **A6**–**A10** showed moderated activity against *E. coli* among which compound **A9** exhibited the greatest antibacterial behavior with an IC_50_ value of 2.08±8.4, A6 (IC_50_ = 2.5±7 mg/mL) and **A10** (IC_50_ = 2.5±15 mg/mL) were moderately active, whereas the remaining compounds **A7** and **A8** showed poor activities against *E. coli* with IC_50_ values of 3.67±4.8 and 3.125±5.6 mg/mL, respectively.

**Table 2:** Antibacterial Activities of Compounds **A6**–**A10** (IC_50_, mg/mL)

		**IC**_**50**_mg/mL	**500 μg/mL**	100 μg/mL	**IC**_**50**_mg/mL	500 μg/mL	100 μg/mL	**IC**_**50**_mg/mL	500 μg/mL	100 μg/mL
		***Klebsiella***			***Staphylococcus aureus***			***E. coli***		
**sr. no.^a^**	**code^b^**		**ZOI Mm**			**ZOI Mm**			**ZOI Mm**	
**1**	**A6**	2 ± 7	12	2	6.25 ± 2.8	4	0	2.5 ± 7	10	0
**2**	**A7**	1.32 ± 8.9	18.8	5	1.38 ± 9	18	5	3.67 ± 4.8	6.8	0
**3**	**A8**	1.19 ± 11	21	5	12.5 ± 1.4	2	0	3.125 ± 5.6	8	0
**4**	**A9**	1.1 ± 12	22.7	5	3.571 ± 4.9	7	0	2.08 ± 8.4	12	0
**5**	**A10**	1.16 ± 11	21.5	5	4.55 ± 3.9	5.5	0	2.5 ± 15	32	10
**6**	control	0.74 ± 16	34	10	0.78 ± 15	32	10	0.73 ± 11	34	18

a Standard error of mean of three assays.

b Positive control used in the assays. Data shown are values from triplicate experiments.

**Figure 3:**
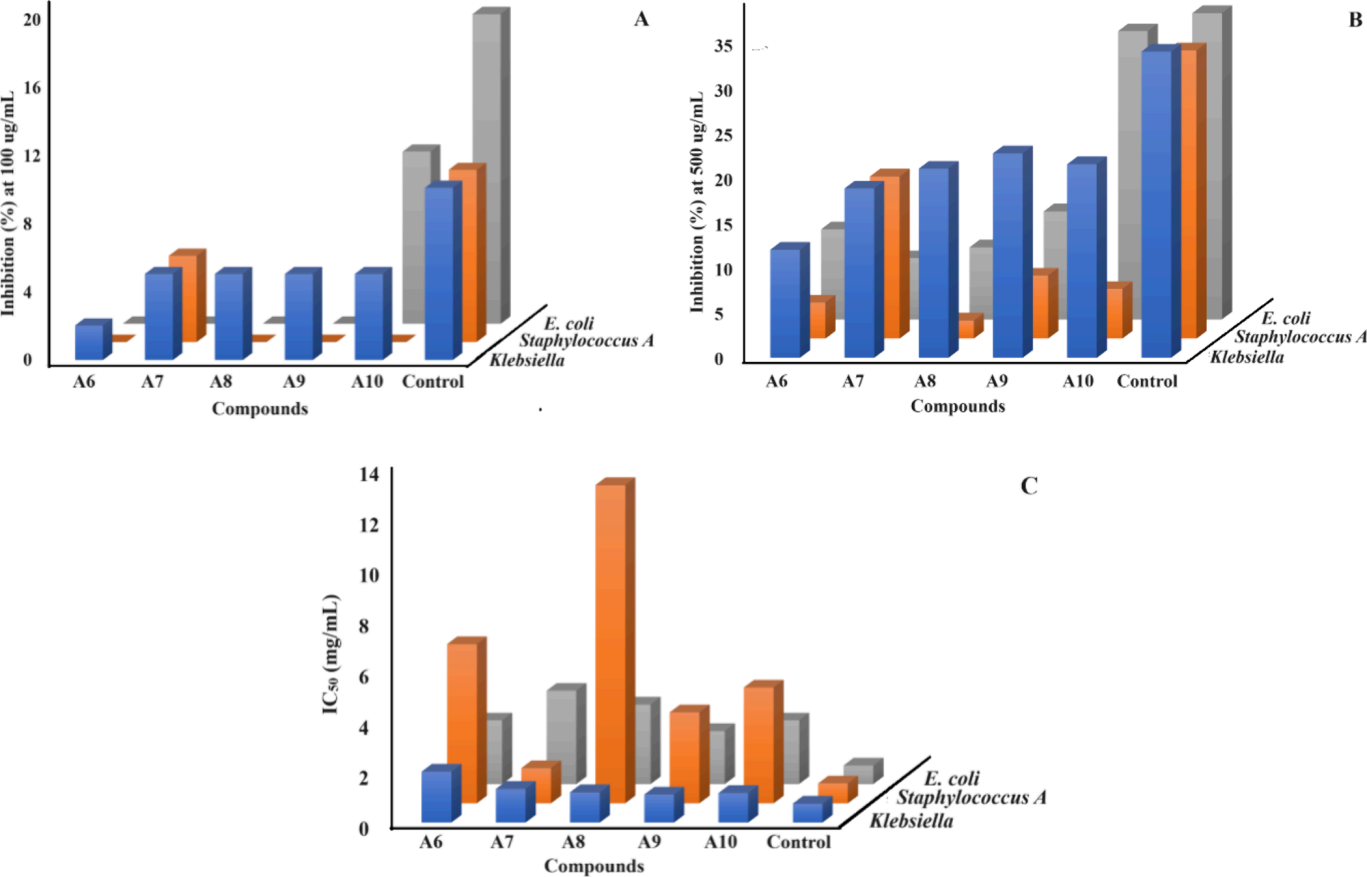
Graphical representation depicting the results of antibacterial assays of **A6**–**A10**, where (A) inhibition (%) at 100 μg/mL, (B) inhibition (%) at 500 μg/mL, and (C) IC_50_ graphs of screened derivatives.

Furthermore, the inhibition percentages at 100 and 500 μg/mL and IC_50_ values against three bacterial strains *Klebsiella*, *S. aureus*, and *E. coli* represented in Figure 3A–C depicts an easily understandable comparison of the potencies of the synthesized motifs. Figure 3A depicts the moderate antibacterial potential of compounds **A7**, **A8**, **A9**, and **A10** with respect to the standards at the 100 μg/mL concentration drug against *Klebsiella*, whereas numerous increments in the efficiencies of synthesized motifs were found when the concentration was increased to 500 μg/mL; for instance, compound **A10** exhibited an inhibition percent comparable to the reference, as shown in Figure 3B. Similarly, Figure 3C represents the IC_50_ evaluation, which reveals that the potencies of **A7**, **A8**, **A9**, and **A10** were similar to the reference drug, indicating them to be promising candidates for further investigations against *Klebsiella*, while compound **A7** was found an auspicious candidate against *S. aureus* and the screened compounds were found to be moderately active against *E. coli*. These results suggest detailed investigations regarding the antimicrobial potential of the aminomethylene derivatives.

### 3.4 Computational Studies

#### 3.4.1 DFT Studies

##### 3.4.1.1 Frontier Molecular Orbital (FMO) Analysis

The excited-state characteristics of molecules involved in charge transfer are studied with the help of FMO analysis. For effective reactivity, a better charge transfer mechanism between the highest and lowest orbitals with the minimum energy gap (*E*_g_) is necessary. The lower the *E*_g_ value, the easier the electron movement from HOMO to LUMO, where HOMO refers to bonding characteristics and LUMO relates to antibonding characteristics, and so *E*_g_ affects molecular stability.^56^ In pharmacological and biological research, the HOMO–LUMO excitation gaps are widely used to evaluate the degree of ICT and the lowering of the energy gap enhances molecular biological activity.^57^

**Figure 4:**
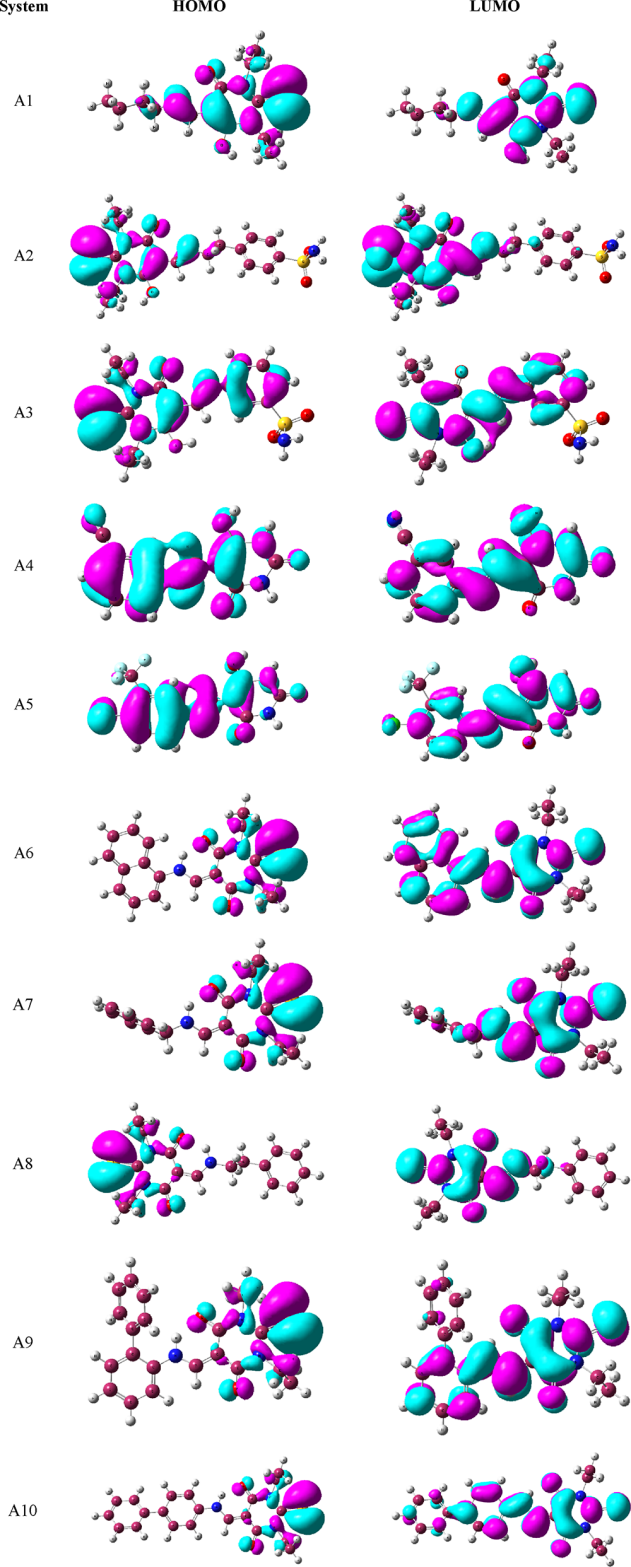
Frontier molecular orbitals of newly synthesized candidate ligands **A1**–**A10**.

The FMOs of all molecules are shown in Figure 4 with their values in Table S1. The *E*_g_ values are in the range of 3.590–5.167 eV. Compound **A1** has the greatest energy gap because it has the least amount of conjugation than other molecules. **A10** and then **A6** have the least values of energy gap due to the highest amount of conjugation. The molecules like **A2**, **A3**, **A4**, and **A5** have electron-withdrawing groups attached to benzene, which decreases the electron transfer to the other side of the molecules. Due to this, these molecules having a high-energy gap and electron transfer require more energy than the remaining molecules. Thus, the HOMO–LUMO energy gap shows the following increasing order for all compounds: **A10** < **A6** < **A9** < **A7** < **A8** < **A3** < **A5** < **A4** < **A2** < **A1**.

##### 3.4.1.2 UV–Visible Study

**Figure 5:**
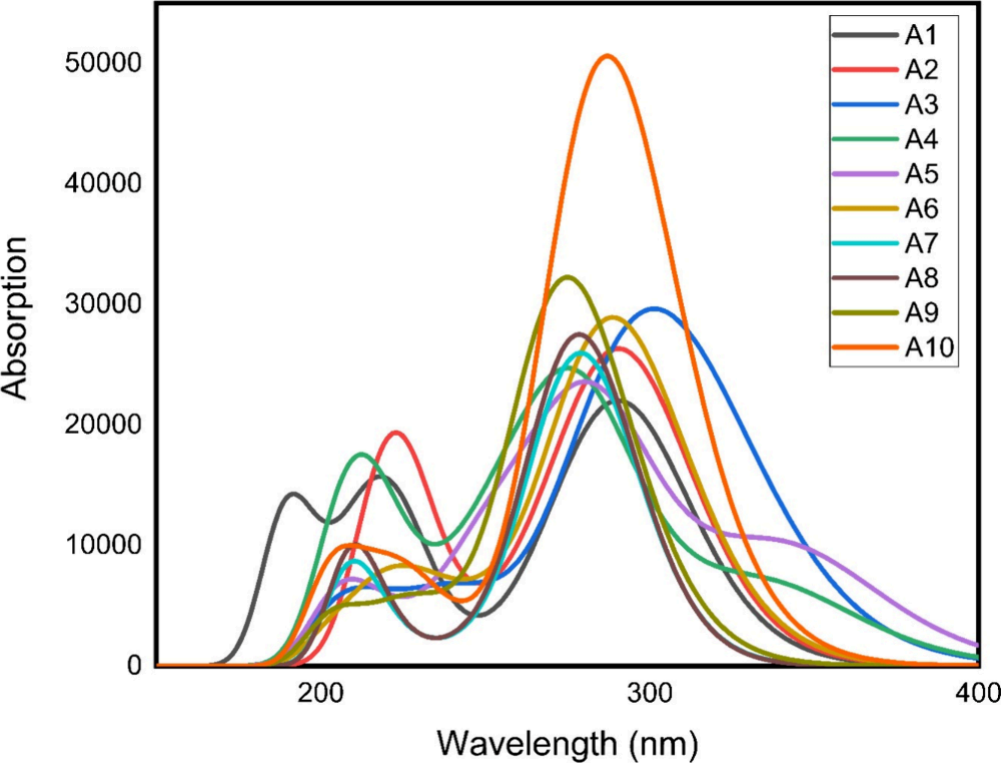
TD-DFT-calculated absorption spectra of all the synthesized candidate ligands **A1**–**A10**.

UV–vis spectroscopy is a useful method for studying the transition of electrons because it analyzes the charge transfer probability in a molecule and determines MOs to the transition. The correlation between UV–Visible spectral data and biological outcomes is pivotal in understanding the pharmacological potential of compounds under investigation.^58^ Understanding the electronic transitions of substances by UV–Visible spectroscopy is helpful in identifying their structural characteristics.^59^ The interactions of the compounds with biological targets are influenced by the absorption patterns found in the UV–Visible spectra, which frequently correspond to particular functional groups.^60^ It enables to forecast the behavior of screened compounds in biological systems by establishing a relationship between spectral properties and biological outcomes, thus improving compound screening efficacy and directing the design and optimization of compounds for specific and successful pharmacological uses in drug discovery.^61^ UV–Visible transitions were investigated using the time-dependent DFT (TD-DFT) performed at the B3LYP functional using the basis set 6-31G(d,p), as shown in Figure 5. Table S2 displays the absorbance maxima, oscillator strength (*f*), and molecular orbitals.^62^ The absorption of **A6** and **A10** molecules required the longest wavelength and was completely justified. In comparison, **A2**, **A4**, and **A5** had the highest excitation energy and the lowest absorption wavelength.^63^**A2** and **A4** had only one electronegative element and had comparable values of wavelength. There is a direct relation of conjugation with wavelength, and due to this, the molecules having a high degree of conjugation like **A10** and **A6** showed higher values of wavelength. These results of UV–Visible studies may be useful as fingerprints for the identification of these compounds in the process of dug development, drug administration in biological systems, and behavior of ligands before and after interacting with their biological targets.

##### 3.4.1.3 Vibrational Analysis

**Figure 6:**
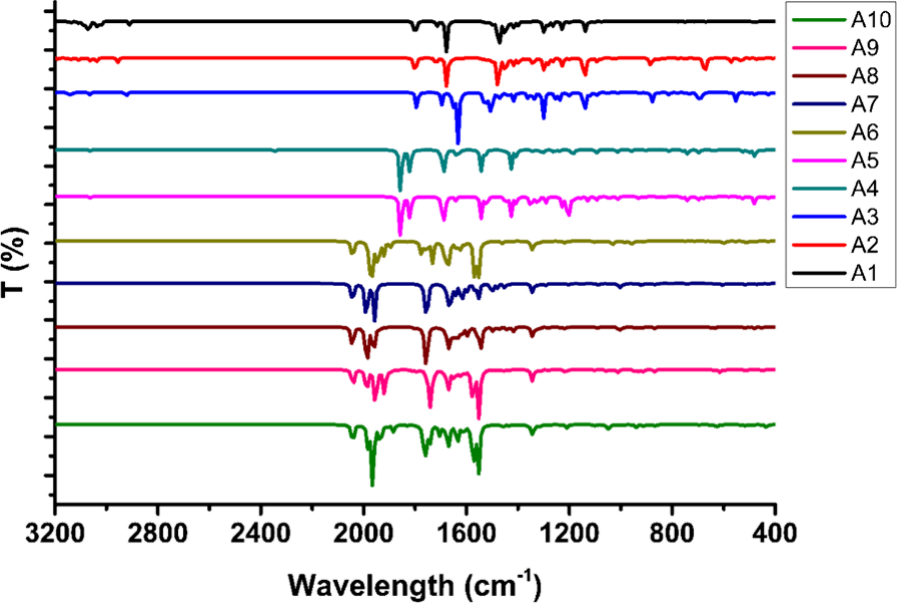
Vibration frequency charts of compounds **A1**–**A10**.

The chemical stability and characterization of a system are well described with the help of the IR spectrum. According to the literature, the chemically stable molecule and noncovalent interactions produce peaks in the near IR region. Due to this, the IR spectra of the molecules are simulated using harmonic approximation and the resulting spectra are displayed in Figure 6.^64^ The peaks for aromatic sp^2^ C–H are shown at 3030–3190 cm^–1^ for the **A1**–**A10** molecules.

##### 3.4.1.4 NMR Chemical Shifts

Following NMR data have been extracted from the computational studies of a test compound:

^1^H NMR (*d6*-DMSO, ppm): 7.74 (1H, br.s, −O***H***), 4.31–4.36 (4H, q, *J* 6.8, −C***H*_2_**–CH_3_), 4.28 (1H, s, −N=C***H***−), 2.93–2.99 (1H, m, −N–C***H***<), 1.86–1.88 (2H, m, 2-CH–C***H***–CH_2_), 1.67–1.73 (2H, m, 2-CH_2_–C***H*_2_**–CH_2_−), 1.56–1.59 (1H, m, −CH_2_–C***H*_2_**–CH_2_−), 1.18–1.25 (4H, m, C2 and C3 ring ***H***), 1.07–1.11 (7H, m, 2-CH_2_–C***H*_3_** and ring-CH_2_–C***H*_2_**-CH_2_−). A summary of the comparison of chemical shift values of both experimental and estimated values corresponding to peak assignments of compounds **A1**–**A10** is shown in Table S3 of the Supplementary Data.

DMSO-*d*_6_ was used as the solvent to record the ^1^H NMR spectra of these systems, while TMS served as the internal reference. The calculations were performed at the B3LYP functional using the GIAO technique and a basis set of 6-31G (d).^65^ The proton chemical shifts were converted to the TMS scale using δ= Σ_o_ – Σ, where δ denotes the chemical shift, Σ is for absolute shielding, and Σ_o_ is used for the absolute shielding of TMS, with a value of 31.88 at B3LYP/6-311+G(2d,p).

**Figure 7:**
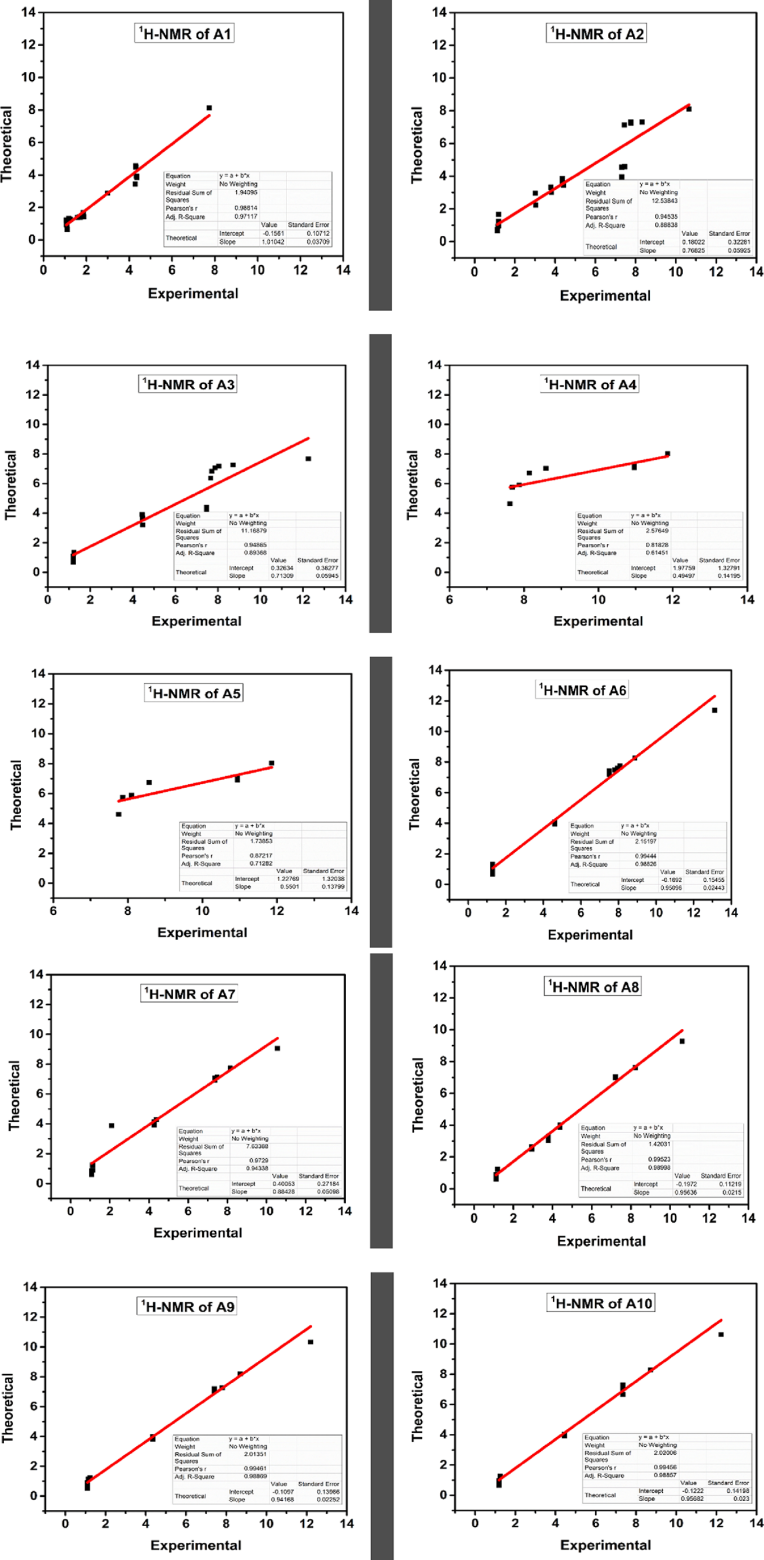
Correlation graphs of experimental and estimated proton chemical shifts of synthesized compounds **A1**–**A10** at the B3LYP level of theory.

The correlation graphs are presented in Figure 7, for comparison with the experimental values. The ^1^H NMR correlation coefficients for **A1**–**A5** are listed as 0.986, 0.9454, 0.9486, 0.8183, and 0.872, whereas for **A6**–**A10**, these values are around the range of 0.994–0.995 using the DFT-B3LYP/6-31G(d) method. An outstanding agreement can be observed between theoretically estimated and experimentally observed proton chemical shift values.

#### 3.4.2 Drug-Likeness and Drug Scores

**Figure 8:**
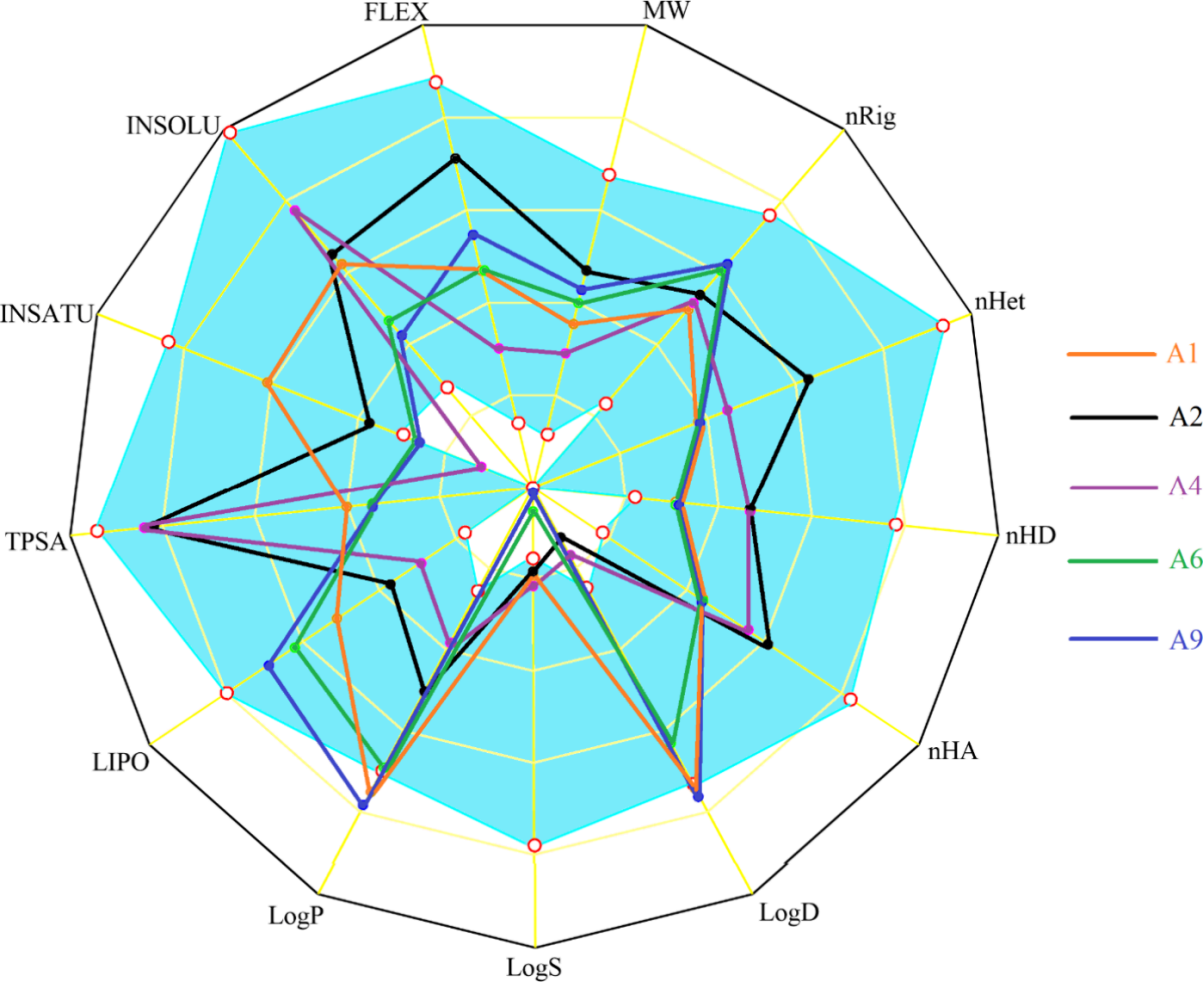
Schematic representation of physiochemical descriptors. The colored zone is the optimal physiochemical space for which molar mass: 100 < MW < 600, number of rigid bonds: 0 < *n*Rig <30, number of heteroatoms: 1 < *n*Het <15, number of hydrogen bond donors: 0 < *n*HD < 7, number of hydrogen bond acceptors: 0 < *n*HA < 12, LogP at physiological pH 7.4:1 < LogD < 3, Log of aqueous solubility: −4 < LogS < 0.5, Log of the octanol/water partition coefficient: 0 < LogP < 3, lipophilicity: −0.7 < LogP_o/w_ (XLOGP3 < 5.0, topological polar surface area: 0 < TPSA < 140, insaturation: 0.25 < INSATU (Fraction Csp3) < 1, insolubility: −6 < INSOLU (Log S (ESOL)) < 0, Flexibility: 0 < FLEX (Num. rotatable bonds) < 9.

Compounds **A1**, **A2**, **A4**, **A6**, and **A9** were selected for screening of their medicinal status using ADMETlab 2.0 and SwissADME, and the important results are represented in Figure 8 below and Table S4 in the Supplementary Data. It was important to notice that a number of molecular descriptors were lying in the predefined optimal ranges for all of the screened motifs while there were some variations in the rest of the properties such as logD values for **A1** and **A9** of 3.003 and 3.012, respectively, which are slightly higher. On the other hand, the values for **A2** and **A4** were estimated to be 0.532 and 0.693, respectively, which were somewhat lower than the optimal range; however, compound **A6** fulfilled these criteria with logD of 2.62. Similarly, compounds **A6** and **A9** deviated from ideality with the logS values of −4.841 and −4.965, respectively, while the estimated logP values for compounds **A1** and **A9** were 3.305 and 3.395, respectively, indicating minor deviations. From the results, it was noticed that each of the screened derivates fulfilled the criteria settled by the Lipinski rule and the golden triangle rule; however, **A1** and **A9** were those that deviated from the Pfizer rule due to its logP values of 3.305 and 3.395, respectively, which are somewhat greater than the optimal range. Assurance of the GSK rule by a molecule is an indicator for a good ADMET profile, and in the present studies, compounds **A1**, **A4**, **A6**, and **A9** fulfilled the criteria of the GSK rule except **A2**, which is digressing due to a slight increase of the molecular mass of 410 instead of 400 g/mol. In compounds **A1**, **A2**, and **A4**, none of the fragments of the structure has been shown to be responsible to targets other than the intended receptors and thus no chances of false-positive outcomes have been expected for these molecules; however, in **A6** and **A9**, the 1,3-pyrimidindione moiety along with an exocyclic double bond was found to be susceptible for false-positive results and this is only one alert of the structural fragment to make the molecule as PAINS. The outcomes of toxicophoric studies of the synthesized motifs were surprisingly interesting in which compounds **A1**, **A2**, and **A4** were entirely nontoxic except some alerts in the FAF-Drugs4 rule for toxicity due to the presence of mainly >C=S and >C=N– moieties in the structures of these compounds or the presence of the −CN functionality; however, various toxicity alerts for compounds **A6** and **A9** were estimated such as presence of an α,β-unsaturated amide functional group, naphthyl fragment, a C–N bond of aniline moiety in **A6** and **A9**, which were found to be susceptible to carcinogenicity or mutagenicity. Presence of one of the two tertiary nitrogen atoms may lead to nongenotoxic carcinogenicity. Presence of the C–N bond of the aniline fragment, α,β-unsaturated amide functional group, and thiourea fragment connected to another carbonyl was estimated to be responsible for skin irritation, and aquatic toxicity was connected with the presence of the α,β-unsaturated amide in compounds **A6** and **A9**.

In the presence screening, **A1**, **A4**, **A6**, and **A9** were lying in the white portion of the BOILED-EGG model as non-substrates of P-glycoprotein enabling them to be considered in the category of non-CNS drug candidates; however, although compounds **A1**, **A6**, and **A9** were in the white portion, they were present near the yellow portion and thus such compounds may be considered as CNS drug candidates. These high-throughput screenings with encouraging outcomes make the synthesized scaffolds potential candidates in the field of drug discovery and development.

#### 3.4.3 OSIRIS and DataWarrior Property Calculations

Data in Table 3 represent a comparison between the newly synthesized Schiff bases **A1**–**A5** and two standard drugs, i.e., penfluridol and amiodarone. The results reveal that Schiff bases are much better than standard drugs in many respects such as all of these Schiff bases that obey the Lipinski rule of five; however, penfluridol and amiodarone di not, at all. Drug-score values are also much better for Schiff bases than reference compounds as the drug score predicted for compound **A2** is 0.79, which is highest of all, and the same compound showed a drug-likeness value 6.75, which is much better than any of 4.07 and 4.26, predicted for penfluridol and amiodarone, respectively.

**Table 3:** Physicochemical, Drug-Likeness, Drug Scores, and Toxicity Risk Calculations Using OSIRIS Property Explorer and DataWarrior

**compounds**	**solubility**	**molweight**	**cLogP**	**cLogS**	**H-acceptors**	**H-donors**	**relative PSA**	**TPSA**	**drug-likeness**	**drug score**
**A1**	–3.66	309.0	1.81	–3.665	5	1	0.2970	88.23	–1.93	0.47
**A2**	–3.42	410.0	0.87	–3.425	8	2	0.3867	156.7	6.75	0.79
**A3**	–3.15	382.0	0.33	–3.152	8	2	0.4256	156.7	0.55	0.41
**A4**	–4.07	256.0	–0.27	–4.066	7	3	0.4495	114.5	–0.13	0.29
**A5**	–4.81	333.0	1.34	–4.807	6	3	0.3391	90.79	–3.67	0.18
penfluridol	–6.76	523.0	7.67	–6.76	2	1	0.0446	23.47	4.07	0.13
amiodarone	–8.02	645.0	6.28	–8.012	4	2	0.1068	42.68	4.26	0.11

**toxicity risks**				
compounds	mutagenicity	tumorigenicity	irritant effects	reproductive effectiveness
**A1**	no risk	no risk	no risk	no risk
**A2**	no risk	no risk	no risk	no risk
**A3**	no risk	no risk	high risk	no risk
**A4**	medium risk	no risk	high risk	no risk
**A5**	medium risk	no risk	high risk	no risk
penfluridol	no risk	no risk	no risk	high risk
amiodarone	no risk	no risk	high risk	no risk

#### 3.4.4 Metabolic Transformations

**Figure 9:**
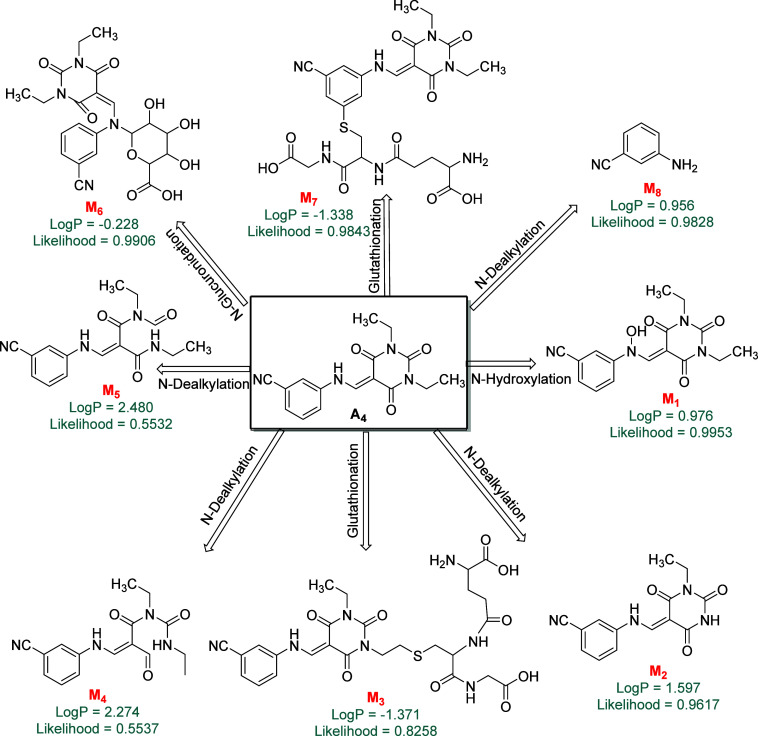
Important metabolites with Pa > Pi of compound **A4** estimated to be produced in phase I and phase II metabolism with calculated LogP and metabolite likelihood scores.

We examined the similarity of metabolites for all newly synthesized compounds using MetaTox, and the outcomes of the investigation of compound **A4** are represented in Figure 9. Among metabolites M_1_, M_2_, M_3_, M_4_, M_5_, M_6_, M_7_, and M_8_, their likeness probabilities are 0.9953, 0.9617, 0.8258, 0.5537, 0.5532, 0.9906, 0.9843, and 0.9828, respectively. This analysis indicated that none of the metabolites have a similarity probability below 0.5. Thus, it can be concluded that if a medication closely resembles its nearest metabolite, there is a higher likelihood that they will both naturally interact with the same target(s). Upon utilizing ProTox-II to assess the potential toxicity of compounds **A1**–**A10** and their metabolites, it has been determined that the overall toxicity risks are moderate to low. Notably, compound **A4** and its metabolites were evaluated for hepatotoxicity, carcinogenicity, immunogenicity, and mutagenicity. The results indicate that all metabolites, except M_2_ that showed a 0.57 probability of carcinogenicity and M_8_ that showed a 0.63 probability of mutagenicity, were determined to be toxically inactive.

In this case, the predicted LD50 for **A4** was 1000 mg/kg. Based on the LD50 values, compound A4 falls within Class IV, which signifies that it is harmful if swallowed. Among the metabolites, M_1_, M_2_, M_5_, and M_8_ also fall within Class IV. M_3_ and M_7_ are classified under Class V (may be harmful if swallowed), M_4_ falls under Class III (toxic if swallowed), and M_6_ is classified as nontoxic (Class VI) based on the LD50 predictions.

### 3.5 Molecular Docking and Molecular Dynamics Simulations

#### 3.5.1 Molecular Docking

The synthesized pyrimidinone-based ligands **A1**–**A10** were docked against the three target substrates HINOS PDB ID: 4NOS, SARS-CoV-2 protease PDB ID: 6Y84, and COVID-19 main protease M^Pro^ PDB ID: 6LU7. The results of the test and reference compounds are mentioned in Table 4.

**Table 4:** AutoDockTools 1.5.6-Based Outcomes of Molecular Docking and Binding Power (Expressed in kcal/mol) of Ligands with the Target Substrates

	**binding energy**	**ligand efficiency**	**inhibition constant (μM)**	**binding energy**	**ligand efficiency**	**inhibition constant (μM)**	**binding energy**	**ligand efficiency**	**inhibition constant (μM)**
**compound**	**4NOS**			**6Y84**			**6LU7**		
**A1**	–7.37	–0.35	3.94	–9.29	–0.44	0.16	–7.45	–0.35	3.44
**A2**	–9.30	–0.34	0.153	–9.76	–0.36	0.07	–7.01	–0.26	7.27
**A3**	–9.21	–0.37	0.177	–10.15	–0.41	0.04	–7.30	–0.29	4.45
**A4**	–7.23	–0.38	5.02	–8.73	–0.46	0.40	–8.36	–0.44	0.75
**A5**	–7.08	–0.32	6.43	–8.33	–0.38	0.78	–8.25	–0.38	0.90
**A6**	–9.47	–0.38	0.114						
**A7**	–8.25	–0.38	0.900						
**A8**	–8.49	–0.37	0.597						
**A9**	–10.23	–0.38	0.032						
**A10**	–10.33	–0.38	0.027						
**ronoptrin**	–5.42	–0.32	107.3						
**inhibitor N3**				–10.9	–0.22	0.01	–6.31	–0.13	23.9
**remdesivir**				–9.85	–0.23	0.06	–6.33	–0.15	22.8
**hydroxychloroquine**				–8.61	–0.37	0.49	–6.50	–0.28	17.3
**chloroquine**				–8.72	–0.40	0.41	–6.78	–0.31	10.8

**Figure 10:**
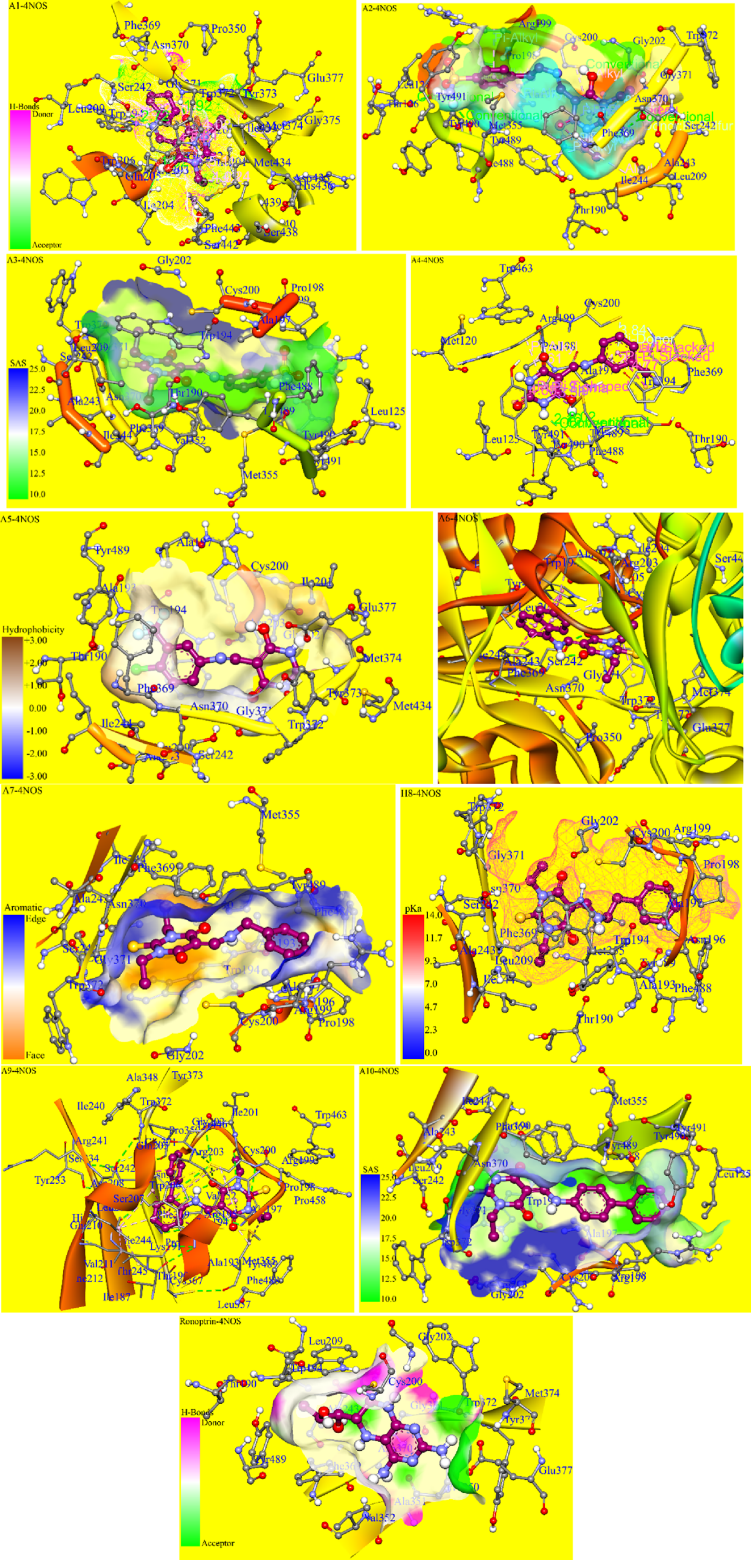
Three dimensional representations of binding analysis, the binding pocket of macromolecule, and best docked conformations of compounds **A1**–**A10**, and the standard inhibitor ronoptrin against chain A of the HINOS enzyme PDB ID: 4NOS.

A careful investigation of the ligand–protein complexes of compounds **A1**–**A10** against chain A of the HINOS enzyme shown in Figure 10 revealed that Cys.200 and Phe.369 amino acids are involved in creating interactions with the pyrimidine ring or the other ring moieties of each of the complexes including Cys.200 involved in a weak alkyl–sulfur interaction (5.07 Å) with the cyclohexyl ring of **A1**; a conventional hydrogen bond (3.02 Å) existed between sulfur and OH functionality on pyrimidinone nucleus; a π–sulfur interaction (5.29 Å) existed between sulfur and π electrons of pyrimidinone of **A2**, **A3**, and **A5**; a π-donor type force (3.84 Å) existed between sulfur atom and aromatic ring of **A4**; a π–alkyl interaction (4.64 Å) between the C–S bond and the A ring of naphthylamine of **A6**; a conventional hydrogen bond (3.54 Å) existed between −SH of Cys.200 and carbonyl of the pyrimidinone nucleus; a π–alkyl interaction (5.29 Å) between the C–S bond and π-electrons of the pyrimidinone ring of **A7**, **A8**, and **A9**; and in compound **A10**, a conventional hydrogen bond (3.08 Å) existed between oxygen of C=O and −SH of Cys.200. A π–alkyl interaction (5.38 Å) between the C–S bond and pyrimidinone nucleus, an alkyl–sulfur interaction (5.16 Å) between the ethyl moiety and the C–S bond, and a π–alkyl force (5.41 Å) between the aromatic ring of biphenyl attached to the NH group and the C–S bond were present. It was worth noting that Cys.200 finds a central position in a cluster of other amino acid residues by creating forces of interaction with them along with the ligand molecule including a conventional H-bond (3.38 Å) with Gly.202, two π–sulfur forces (4.47 and 5.93 Å) with both aromatic rings of Trp.194, two conventional hydrogen bonds (3.76 and 2.92 Å) and an alkyl–sulfur force (4.14 Å) with Arg.203, and a conventional H-bond (3.08 Å) and an alkyl–sulfur interaction (4.50 Å) with the Ala.197 amino acid. Similarly, the aromatic π-electronic cloud of Phe.369 also exerts a major influence in creating the forces with the ligand molecule in which no force exists with compound **A1** because there are no aromatic clouds that could be involved in π–π interactions; however, in some of the compounds especially where the aromatic system was one or two carbons apart from the amino moiety such as **A2**, **A3**, **A7**, **A8**, and **A10**, there existed π–π-stacked and π–alkyl forces with the pyrimidinone nucleus and ethyl moiety, respectively, while in the rest of the compounds **A4**, **A5**, **A6**, and **A9**, π–π-stacked forces of attraction were observed with the aromatic ring systems of the ligand molecules. In the case of the ligand–protein complex of compound **A5**, as a chlorine atom and a −CF_3_ moiety were present, π-lone pair (2.61 Å) interaction was thus observed between the aromatic ring of Phe.369 and one of the three fluorine atoms and a π–alkyl force (3.66 Å) was witnessed between the carbon atom of the trifluoromethyl substituent and the phenyl ring of the Phe.369 amino acid residue. Along with these residues of the macromolecule, Thr.190, Trp.194, Ala.197, Pro.198, Arg.199, Gly.202, Arg.203, Leu.209, Ser.242, Ala.243, Ile.244, Met.355, Asn.370, Gly.371, Trp.372, Phe.488, Tyr.489, Tyr.490, and Tyr.491 amino acid residues were frequently observed in the binding pocket of the target substrate when it complexed to the ligand molecules. The two-dimensional representations of binding pockets and interaction of ligands with the amino acid residues are shown in Figures S40–S49.

Furthermore, by comparing the binding energies of the ligand molecules with target substrates, it was revealed that the binding energy of any of the synthesized ligand molecules (−7.08 to −10.33 kcal/mol) was much higher as compared to the binding energy of the ronoptrin (−5.42 kcal/mol), indicating that the newly synthesized scaffolds could bind more strongly than ronoptrin with the target macromolecule proving themselves to be the competitive inhibitors with the naturally bound ligand. Similarly, the ligand efficiency values (−0.32 to −0.38 kcal/mol) of the screened compounds proved these scaffolds to be better inhibitors of the NO synthase enzyme than ronoptrin (−0.32 kcal/mol).

The probe of the ligand–protein complexes of compounds **A1**–**A5** with the SARS-CoV main protease enzyme revealed the involvement of Phe.3, Arg.4, Lys.5, Met.6, Ala.7, Val.125, Tyr.126, Gln.127, Cys.128, Lys.137, Gln.138, Ser.139, Trp.207, Leu.282, Ser.284, Glu.288, Glu.290, and Phe.291, in the binding cavity of the substrate, responsible for creating interactions with the ligand molecules. The interacting amino acids with the ligand molecule A1 included Lys.5 through a conventional hydrogen bond between the C=O bond of the pyrimidinone moiety and the −NH_3_^+^ of the lysine residue with a bond distance of 1.86 Å; the −CH_3_ group of Ala.7 formed a non-polar alkyl–alkyl type long-range interaction with the cyclohexyl moiety with a distance of 4.53; the aromatic ring of Tyr.126 interacted with one of the ethyl substituents at nitrogen atoms via π–sigma and π–alkyl forces with bond distances 3.84 and 4.50 Å; Gln.127 played an important role in binding the protein with the ligand molecule via conventional hydrogen bonds of the NH moiety of amino acid with the −OH group at pyrimidinone nucleus with a 2.74 Å distance and with the nitrogen atom of the Schiff base moiety by the bond distance of 2.06 Å; the C–S bond of Cys.128 interacted via a non-polar force with the ethyl substituent being interlinked with Tyr.126 with a distance of 4.46 Å; and the oxygen atom of the carboxylate functionality for π-anion type interaction with the pyrimidinone ring with the bond distance of 4.89 Å. The greater size and more functionalities of compound **A2** cause the key variations in the amino acids and their interactions with the ligand molecule despite occupying the same binding pocket as **A1**; for instance, Phe.3 and Arg.4 replaced the Met.6 and Ala7 present in the case of **A1**, in which Arg.4 is involved in creating various kinds of interactions including π–cation (3.92 Å) between the NH group of amide of the peptide bond with Lys.5 and π electrons of the pyrimidinone ring, a conventional hydrogen bond (1.96 Å) between one NH of the guanidino moiety and OH group present at the pyrimidinone cycle, and a van der Waals interaction (2.71 Å) between the carbon atom alpha to the guanidino moiety and the OH group. The long-range π–π T-shaped force (5.55 Å) between the π electronic cloud of the aromatic ring containing the sulfonamide moiety and π electrons of the pyrimidinone ring and two other π-alkyl attractive forces (4.18 and 4.51 Å) of the alkyl chain of Lys.5 with electronic clouds of both rings of the ligand molecule wields the twisting of the ligand structure in which the flexibility of the ligand structure due to rotatable bonds of the ethylene fragment plays their decisive role and this puckering facilitates the terminal −NH_3_^+^ moiety of Lys.5 to create strong conventional hydrogen bonds (2.27, 2.29, and 3.09 Å) with the oxygen atoms of the sulfonamide moiety on one side and a weak van der Waals force (3.28 Å) with the ethyl substituent at the nitrogen atom of the pyrimidinone ring. Another important variation is the presence of Ser.284 and Phe.291, which were creating a conventional hydrogen bond (2.85 Å) through the OH group and a π–sulfur force (5.90 Å) through the aromatic electronic cloud, respectively, with the sulfur atom present at the pyrimidinone fragment.

**Figure 11:**
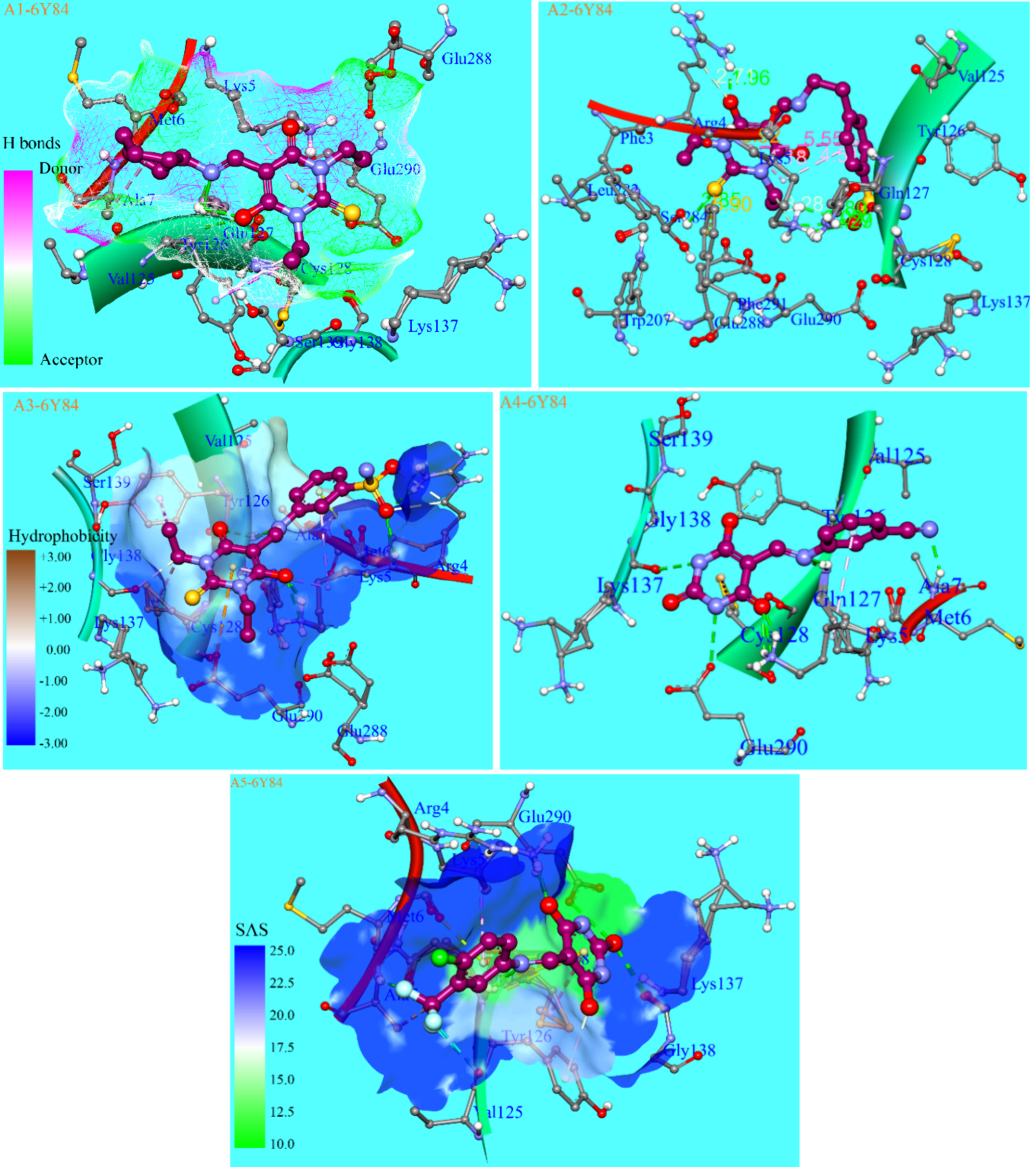
Three-dimensional representations of binding analysis, the binding pocket of macromolecule, and best docked conformations of compounds **A1**–**A5** against the SARS-CoV-2 main protease enzyme PDB ID: 6Y84.

As the synthesized motifs bear the presence of multiple functional parts of different nature, which offers an important role in the creation of binding forces with the amino acid residues in the binding pocket of protein such as compound **A3** affording a sulfonamide moiety at the aromatic ring meta to the amine functionality and thus making it polydentate for Lys.5 as the NH forming the peptide linkage with Arg.4 of this residue forms an HB (2.40 Å) with the oxygen atom of the sulfonamide moiety, its alkyl chain is responsible for two nonpolar π–alkyl type forces with the electronic cloud of the phenyl moiety (4.86 Å) and π electrons of the pyrimidinone ring (5.45 Å), and two conventional hydrogen bonds (1.79 and 2.67 Å) by the ammonium ion of this amino acid with the −OH functionality located at the pyrimidinone fragment of the ligand molecule. The guanidine part of the neighboring Arg.4 also formed an HB (2.24 Å) with the oxygen atoms of the sulfonamide functional group. Gln.127 also played its role in slightly twisting the ligand molecule by creating two hydrogen bonds with the oxygen atom of C(O)*N* < functionality (3.08 Å) and the =NH– of the amino methylene linkage (2.36 Å), whereas another unique and prominent π–anion force (4.66 Å) existed between the COO– of the Glu.290 and the π-electrons present in the pyrimidinone ring. The rest of the analyzed molecules against the same enzyme resulted in similar types of binding interactions in the binding pocket of macromolecules, which are also shown in Figure 11 and Figures S50–S59.

## 4 Conclusions

The target compounds were constructed via a three-component single-step reaction in good yields, and their structures were elucidated using various techniques including 1D and 2D NMR experiments, HRMS, IR, and UV/vis. The DFT studies focusing on the structural properties offered results of spectroscopic analysis, which were in good agreement with the experimental ones. The DPPH assays resulted in excellent potency of the screened compounds **A6**–**A10** as antioxidants with IC_50_ values of 0.83 ± 0.125, 0.90 ± 0.77, 0.36 ± 0.063, 1.4 ± 0.07, and 1.18 ± 0.06, which were much better than that of the reference ascorbic acid of 1.79 ± 0.045. All the compounds exhibited better antibacterial potency against *Klebsiella* with IC_50_ values of 2 ± 7, 1.32 ± 8.9, 1.19 ± 11, 1.1 ± 12, and 1.16 ± 11 for **A6**–**A10**. The synthesized compounds were scrutinized by molecular docking analyses against HINOS, and the results were compared with the reference compounds against two target substrates, namely, SARS-CoV-2 protease and COVID-19 main protease M^Pro^, while ronoptrin against the HINOS enzyme. The binding energy values (−9.29, −9.76, −10.15, −8.73, −8.33 kcal/mol) for **A1**–**A5** are comparable to any of the standard drugs (−9.85, −8.61, −8.72 kcal/mol) respectively for remdesivir, hydroxychloroquine, and chloroquine against the target substrate SARS-CoV-2 protease. Similarly, compounds **A1**–**A5** showed binding affinities −7.45, −7.01, −7.30, −8.36, and −8.25 kcal/mol with ligand efficiencies of −0.35, −0.26, −0.29, −0.44, and −0.38 kcal/mol, respectively, against the second target substrate COVID-19 main protease M^Pro^, which are promising as compared to reference compounds (−6.33, −6.50, −6.78 kcal/mol) for remdesivir, hydroxychloroquine, and chloroquine with ligand efficiency values −0.15, −0.28, and −0.31 kcal/mol, respectively. Furthermore, the binding affinities of compounds **A6**–**A10** with HINOS were found to be −9.47, −8.25, −8.49, −10.23, and −10.33 kcal/mol and the corresponding ligand efficacies of −0.38, −0.38, −0.37, −0.38, and −0.38 kcal/mol as compared to the binding energy of −5.42 and ligand efficiency value −0.32 kcal/mol for ronoptrin, therefore proving the target compounds to be more potent having excellent potential to be used as drugs.

## Supporting Information Available

Proteins structures, DFT data, MD simulations plots, physiochemical characteristics, spectral data, and 2D representations of ligand–protein bindings of the newly synthesized compounds (PDF)
