# Supplementary material for: New Pyrimidinone Bearing Aminomethylenes and Schiff Bases as Potent Antioxidant, Antibacterial, SARS-CoV-2, and COVID-19 Main Protease MPro Inhibitors: Design, Synthesis, Bioactivities, and Computational Studies
Source: ACS Omega. 2024 Jun 4;9(24):25730–47. doi: 10.1021/acsomega.3c09393 (PMC11191110; doi:10.1021/acsomega.3c09393)
Supplement: Supplementary file 1 — ao3c09393_si_001.pdf [file ao3c09393_si_001.pdf]

# New pyrimidinone bearing aminomethylenes and Schiff bases as potent antioxidant, antibacterial, SARS-CoV-2 and COVID-19 main protease $M^{Pro}$ inhibitors: Design, synthesis, bioactivities and computational studies

Muhammad Sarfraz[a], Muhammad Ayyaz[a], Abdul Rauf[a*] Asma Yaqoob[b], Tooba Shafa[b], Muhammad Arif Ali[a], Sabir Ali Siddique[a], Ashfaq Mahmood Qureshi[c], Muhammad Hassan Sarfraz[d]*, Reem M. Aljowaie[e], Saeedah Musaed Almutairi[e], Muhammad Arshad[a]*

[a] *Institute of Chemistry, The Islamia University of Bahawalpur-63100, Pakistan*

[b] *Institute of Biochemistry, Biotechnology, and Bioinformatics. Department of Biochemistry, The Islamia University of Bahawalpur-63100, Pakistan*

[c] *Department of Chemistry, Govt. Sadiq College Women University, Bahawalpur, Pakistan*

[d] *Botnar Institute of Musculoskeletal Sciences, Nuffield Department of Orthopaedics, Rheumatology and Musculoskeletal Sciences, University of Oxford, OX3 7LD, UK*

[e] *Department of Botany and Microbiology, College of Science, King Saud University, P.O. 2455, Riyadh 11451, Saudi Arabia*

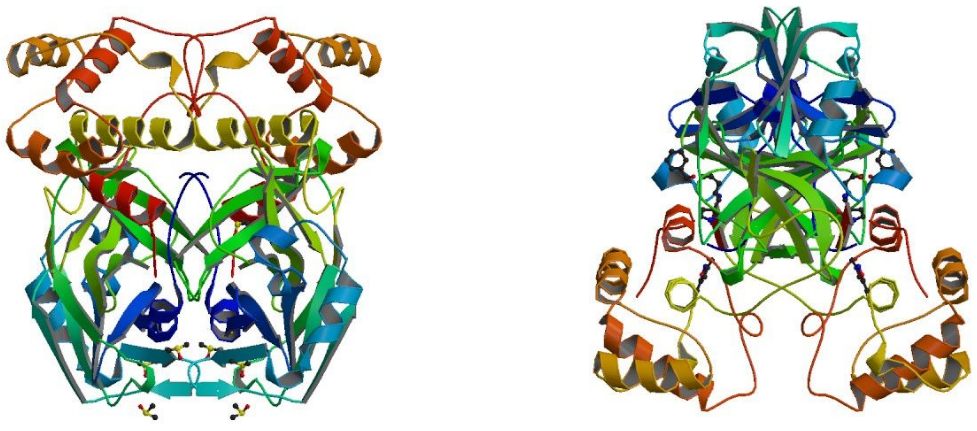

**Figure S1:** Crystal structure of SARS-CoV-2 main protease enzyme with unliganded active site PDB ID: 6Y84 (left) and crystal structure of COVID-19 main protease in complex with an inhibitor N3 PDB ID: 6LU7 (right)

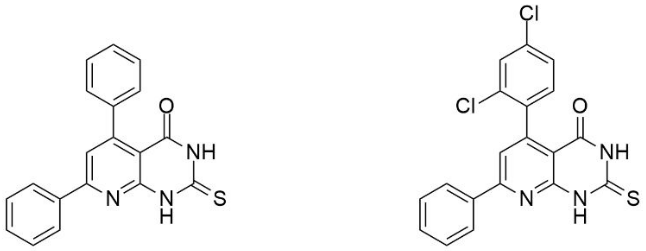


A B

**Figure S2:** Pyrimidinone bearing compounds **(A)**: 5,7-Diphenyl-2-thioxo-2,3-dihydropyrido[2,3-*d*]pyrimidin-4(1*H*)-one and **(B)**: 5-(2,4-dichlorophenyl)-7-phenyl-2-thioxo-2,3-dihydropyrido[2,3-*d*]pyrimidin-4(1*H*)-one as DPPH free radical scavengers

Initially, the geometry optimization of compounds **A1-A5** was accomplished at the B3LYP/6-31G(d) level of theory. Frequency calculations were also carried out at the same level to confirm the absence of imaginary frequencies and extract the theoretical vibrational spectra. Furthermore, the analysis of frontier molecular orbitals (FMO) for compounds **A1-A10** was conducted. For the calculation of electronic excitations, time-dependent density functional theory (TD-DFT) calculations were performed at the B3LYP/6-31G(d) level of theory in the gas phase. To account for solvent effects, the IEF-PCM/B3LYP/6-31G(d) method based on the polarizable continuum model (PCM) was applied.

**Table S1:** The $E_{HOMO}$, $E_{LUMO}$ and $E_g$ of molecules in eV.

| Molecule | HOMO (eV) | LUMO (eV) | $E_g$ (eV) |
|---|---|---|---|
| **A1** | -5.975 | -0.808 | 5.167 |
| **A2** | -6.120 | -1.623 | 4.497 |
| **A3** | -6.114 | -2.097 | 4.017 |
| **A4** | -6.221 | -1.829 | 4.392 |

| | | | |
|---|---|---|---|
| **A5** | -6.106 | -1.848 | 4.259 |
| **A6** | -5.797 | -2.201 | 3.595 |
| **A7** | -5.681 | -1.787 | 3.894 |
| **A8** | -5.689 | -1.785 | 3.904 |
| **A9** | -5.737 | -2.083 | 3.654 |
| **A10** | -5.801 | -2.211 | 3.590 |

**Table S2:** Absorption values ($\lambda_{max}$), oscillator strength (*f*) and molecular assignments are obtained at TD-DFT/ B3LYP/ 6-31G(d,p) level of DFT.

| System | $\lambda_{max}$ (nm) | (*f*) | Molecular Orbital |
|---|---|---|---|
| **A1** | 296.88 | 0.65 | H-1->LUMO (18%), HOMO>LUMO (72%) H-2->LUMO (6%) |
| **A2** | 221.75 | 0.23 | H-6->L+2 (10%), H-3->L+1 (44%) H-5->L+3 (3%), H-4->L+1 (9%), H-3->L+2 (4%), H-2->L+2 (3%) |
| **A3** | 345.04 | 0.67 | H-2->LUMO (16%), H-1->LUMO (18%), HOMO->LUMO (61%) |
| **A4** | 279.28 | 0.53 | H-1->LUMO (68%), HOMO->LUMO (14%), HOMO->L+1 (10%) H-2->LUMO (3%) |
| **A5** | 283.91 | 0.58 | H-1->LUMO (74%), HOMO->LUMO (16%) H-2->LUMO (3%), HOMO->L+1 (4%) |
| **A6** | 392.37 | 0.71 | HOMO->LUMO (98%) |
| **A7** | 310.36 | 0.77 | H-1->LUMO (97%) |
| **A8** | 310.13 | 0.80 | H-1->LUMO (97%) |
| **A9** | 365.12 | 0.83 | HOMO->LUMO (90%) H-1->LUMO (9%) |
| **A10** | 387.02 | 1.29 | HOMO->LUMO (99%) |

**Table S3:** Summary of the comparison of chemical shift values of both experimental and estimated values corresponding to peak assignments of the **A1-A10** synthesized compounds.

| Atom | A1 | | A2 | | A3 | | A4 | | A5 | | A6 | | A7 | | A8 | | A9 | | A10 | |
|---|---|---|---|---|---|---|---|---|---|---|---|---|---|---|---|---|---|---|---|---|
| | Exp (ppm) | Calc (ppm) | Exp (ppm) | Calc (ppm) | Exp (ppm) | Calc (ppm) | Exp (ppm) | Calc (ppm) | Exp (ppm) | Calc (ppm) | Exp (ppm) | Calc (ppm) | Exp (ppm) | Calc (ppm) | Exp (ppm) | Calc (ppm) | Exp (ppm) | Calc (ppm) | Exp (ppm) | Calc (ppm) |
| 28-H | 7.74 | 8.13 | 10.6 | 8.10 | 12.2 | 7.68 | 11.8 | 8.02 | 11.8 | 8.02 | 13.1 | 11.3 | 10.5 | 9.06 | 10.6 | 9.27 | 12.2 | 10.3 | 12.2 | 10.6 |
| 27-H | 4.31 | 4.56 | 5 | 7.31 | 5 | 7.25 | 6 | 7.18 | 6 | 7.01 | 2 | 8 | 6 | 7.74 | 2 | 7.62 | 0 | 3 | 4 | 4 |
| 25-H | 4.32 | 4.48 | 8.32 | 7.29 | 8.71 | 7.17 | 10.9 | 7.07 | 10.9 | 6.90 | 8.87 | 8.26 | 8.15 | 7.13 | 8.23 | 7.04 | 8.70 | 8.19 | 8.73 | 8.29 |
| 26-H | 4.35 | 3.92 | 7.76 | 7.23 | 8.04 | 7.06 | 6 | 7.03 | 4 | 6.74 | 8.08 | 7.75 | 7.48 | 7.07 | 7.22 | 7.04 | 7.80 | 7.27 | 7.35 | 7.31 |
| 24-H | 4.36 | 3.84 | 7.77 | 7.13 | 7.86 | 6.83 | 10.9 | 6.72 | 10.9 | 5.89 | 7.94 | 7.60 | 7.36 | 7.07 | 7.22 | 7.00 | 7.41 | 7.21 | 7.35 | 7.22 |
| 23-H | 4.28 | 3.43 | 7.44 | 4.58 | 7.72 | 6.37 | 6 | 5.90 | 4 | 5.74 | 7.80 | 7.48 | 7.36 | 7.06 | 7.22 | 6.99 | 7.41 | 7.18 | 7.35 | 7.20 |
| 29-H | 2.99 | 2.88 | 7.46 | 4.55 | 7.66 | 4.39 | 8.59 | 5.75 | 8.57 | 4.61 | 7.51 | 7.44 | 7.36 | 6.92 | 7.22 | 6.97 | 7.41 | 7.15 | 7.35 | 7.18 |
| 34-H | 1.86 | 1.67 | 7.31 | 3.95 | 7.47 | 4.11 | 8.14 | 4.63 | 8.10 | - | 7.51 | 7.39 | 7.36 | 4.28 | 7.22 | 3.99 | 7.41 | 7.08 | 7.35 | 7.17 |
| 35-H | 1.88 | 1.44 | 7.31 | 3.86 | 7.47 | 3.91 | 7.87 | | 7.86 | | 7.51 | 7.32 | 4.37 | 4.14 | 4.38 | 3.96 | 7.41 | 7.07 | 7.35 | 7.17 |
| 30-H | 1.67 | 1.43 | 4.36 | 3.71 | 4.43 | 3.87 | 7.69 | | 7.75 | | 7.51 | 7.19 | 4.27 | 3.99 | 4.38 | 3.93 | 7.41 | 7.06 | 7.35 | 7.16 |
| 31-H | 1.73 | 1.40 | 4.36 | 3.58 | 4.45 | 3.67 | 7.62 | | - | | 4.61 | 4.09 | 4.27 | 3.90 | 4.38 | 3.86 | 7.41 | 7.06 | 7.35 | 7.07 |
| 36-H | 1.59 | 1.39 | 4.36 | 3.44 | 4.46 | 3.19 | | | | | 4.61 | 4.05 | 4.27 | 3.88 | 4.38 | 3.29 | 7.41 | 7.02 | 7.35 | 6.67 |
| 37-H | 1.18 | 1.32 | 4.42 | 3.32 | 4.47 | 1.36 | | | | | 4.61 | 4.03 | 2.09 | 3.87 | 3.78 | 3.04 | 4.35 | 4.00 | 4.45 | 4.04 |
| 33-H | 1.19 | 1.29 | 3.79 | 3.01 | 1.23 | 1.28 | | | | | 4.61 | 3.93 | 2.09 | 1.24 | 3.78 | 2.64 | 4.35 | 3.89 | 4.45 | 4.03 |
| 32-H | 1.24 | 1.28 | 3.82 | 2.96 | 1.23 | 1.09 | | | | | 1.30 | 1.32 | 1.12 | 1.22 | 2.94 | 2.49 | 4.35 | 3.86 | 4.45 | 3.97 |
| 38-H | 1.25 | 1.26 | 3.01 | 2.23 | 1.20 | 0.93 | | | | | 1.30 | 1.27 | 1.12 | 0.88 | 2.94 | 1.24 | 4.35 | 3.81 | 4.45 | 3.91 |
| 39-H | 1.07 | 1.22 | 3.04 | 1.67 | 1.20 | 0.80 | | | | | 1.30 | 1.06 | 1.12 | 0.85 | 1.20 | 1.23 | 1.23 | 1.24 | 1.25 | 1.27 |
| 40-H | 1.07 | 1.21 | 1.19 | 1.24 | 1.20 | 0.68 | | | | | 1.30 | 0.91 | 1.07 | 0.62 | 1.20 | 0.90 | 1.14 | 1.14 | 1.25 | 1.26 |
| 41-H | 1.08 | 1.14 | 1.19 | 0.96 | 1.20 | - | | | | | 1.30 | 0.73 | 1.07 | 0.59 | 1.12 | 0.86 | 1.10 | 0.90 | 1.19 | 0.96 |
| 42-H | 1.08 | 0.96 | 1.19 | 0.93 | - | - | | | | | 1.30 | 0.66 | 1.07 | | 1.12 | 0.64 | 1.10 | 0.70 | 1.19 | 0.92 |
| 43-H | 1.09 | 0.90 | 1.16 | 0.72 | - | - | | | | | - | - | | - | 1.12 | 0.61 | 1.10 | 0.64 | 1.19 | 0.68 |
| 44-H | 1.10 | 0.71 | 1.13 | 0.67 | - | - | | | | | - | - | - | - | 1.12 | - | 1.10 | 0.52 | 1.19 | 0.66 |
| 45-H | 1.11 | 0.65 | 1.13 | - | - | - | | | | | - | - | - | - | - | - | - | - | - | - |

| | | | | | | | | | | | | | | | | | | | | |
|---|---|---|---|---|---|---|---|---|---|---|---|---|---|---|---|---|---|---|---|---|
| | | | - | | - | | | | | | - | - | - | | - | | - | - | - | - |

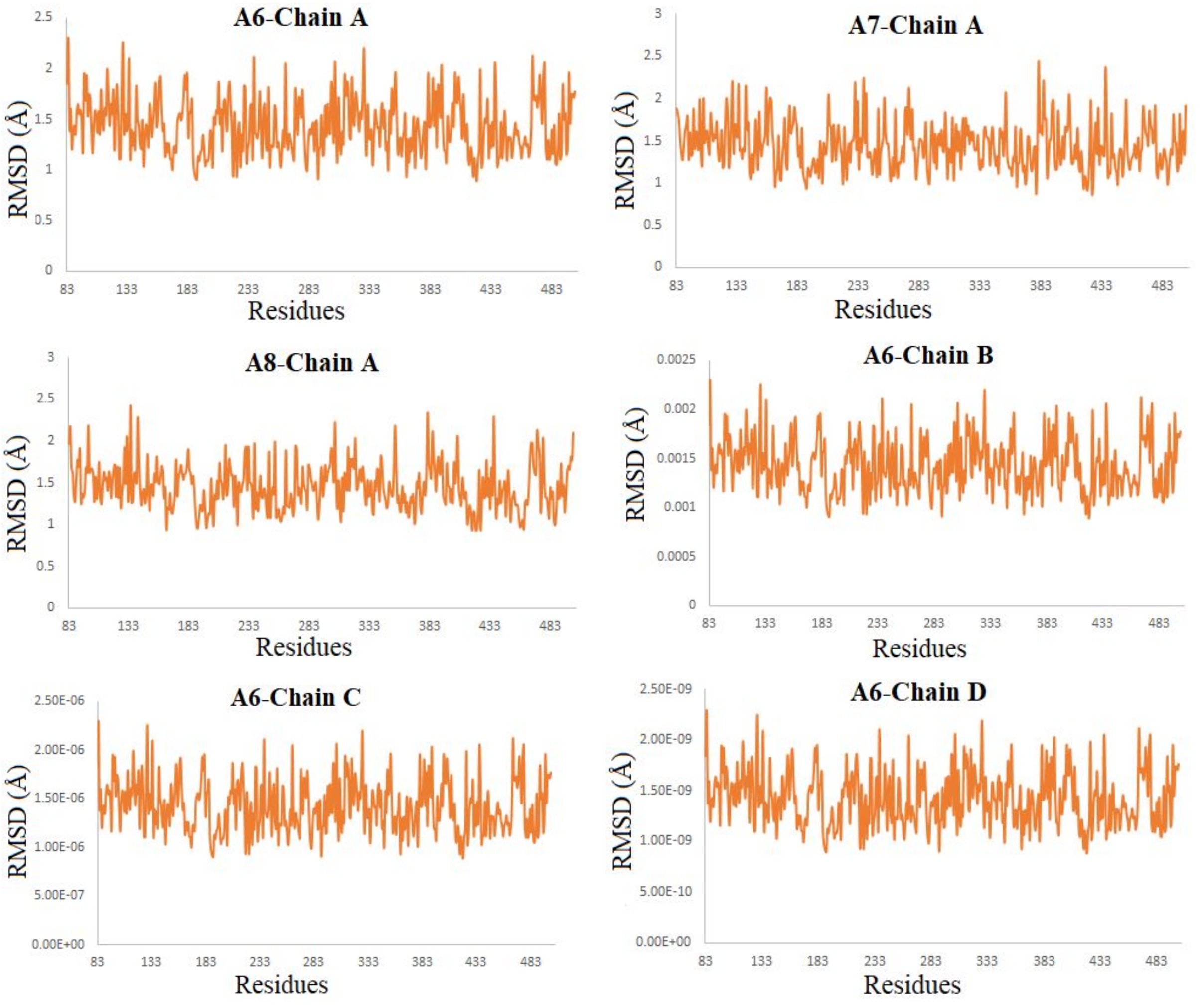


**Figure S3:** RMSD plots amino acid residues of ligand protein complexed formed by compounds **A6** (Chain A, B, C, and D), **A7** (Chain A) and **A8** (Chain A)

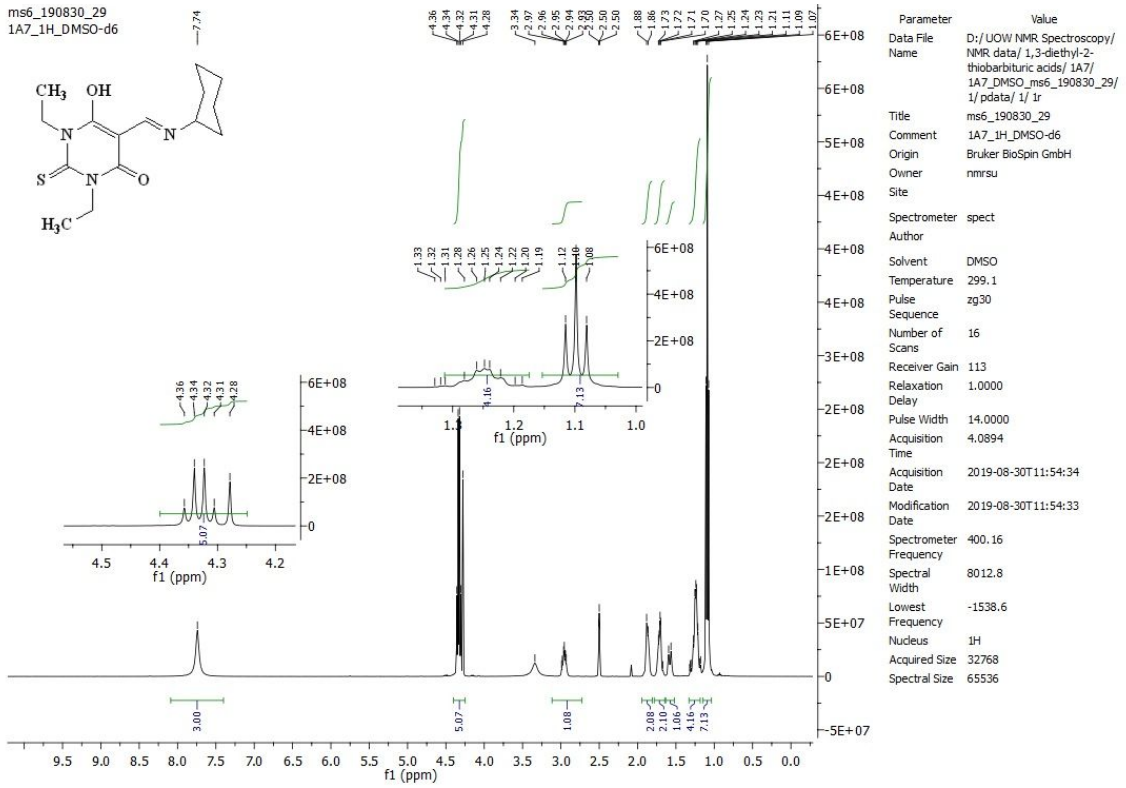


**Figure S4: $^{1}$H NMR Spectrum of compound A1**

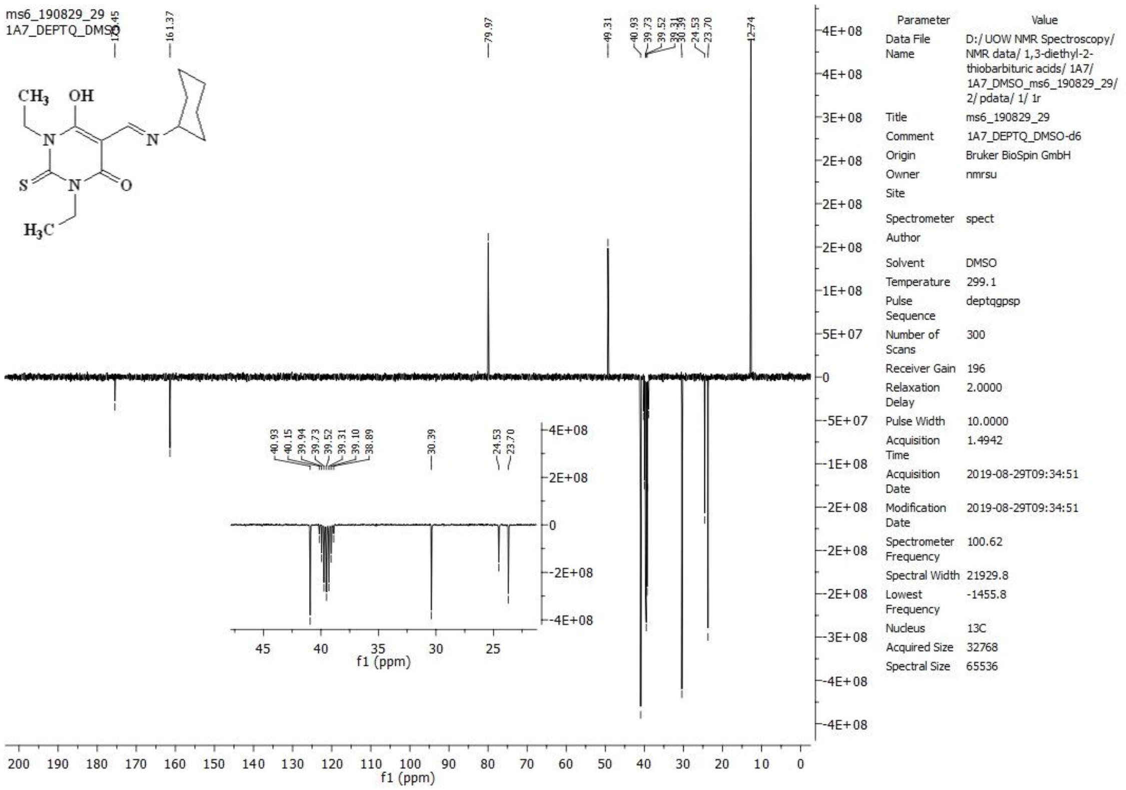


**Figure S5: $^{13}$C NMR (DEPTQ) Spectrum of compound A1**

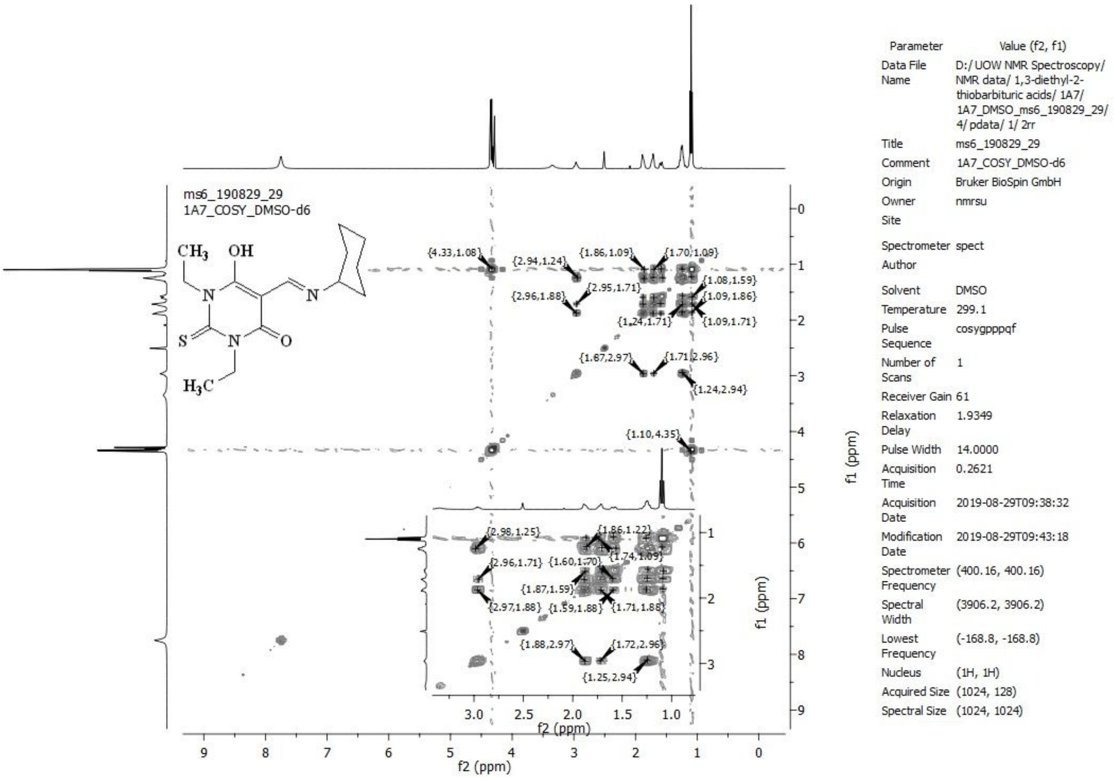


**Figure S6: COSY Spectrum of compound A1**

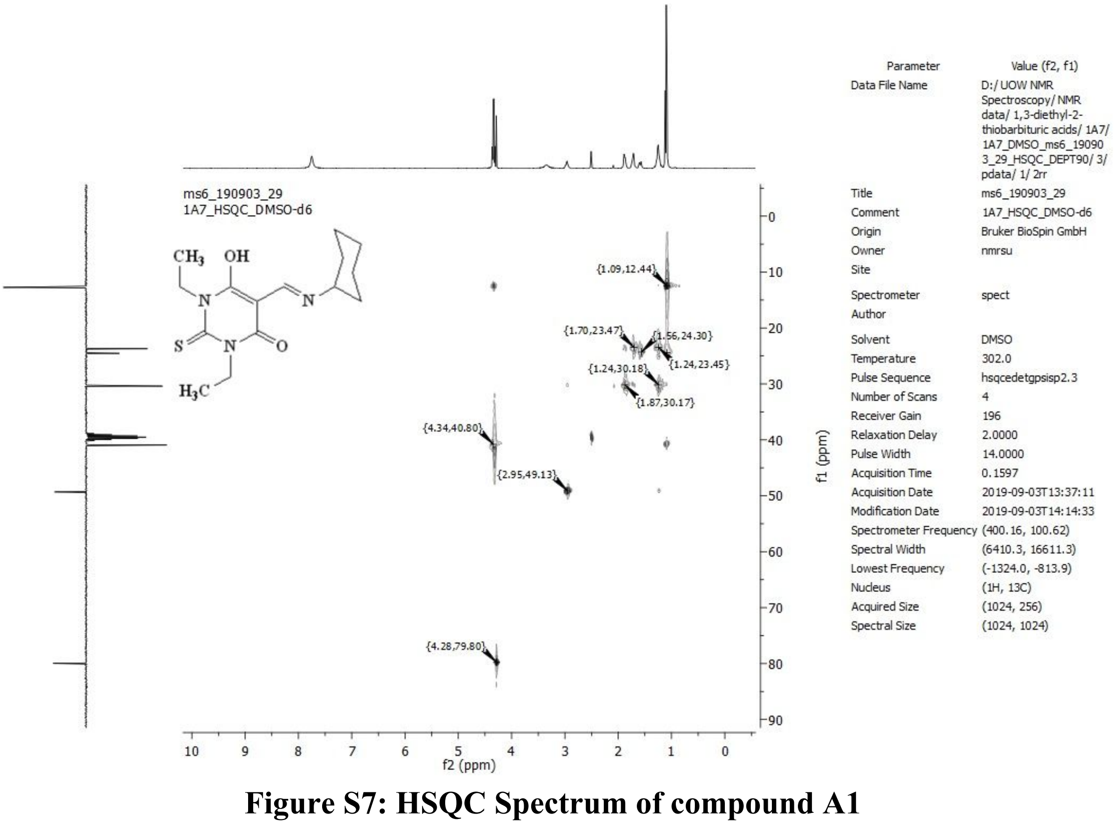


**Figure S7: HSQC Spectrum of compound A1**

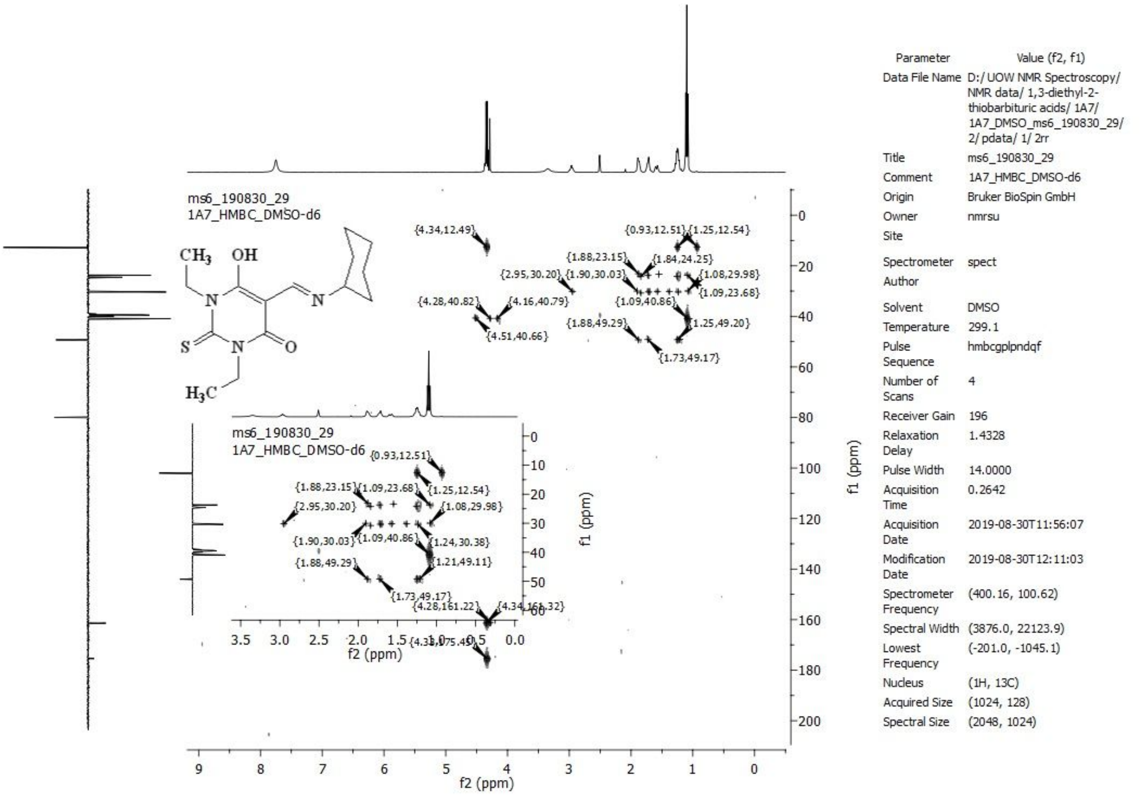

**Figure S8: HMBC Spectrum of compound A1**

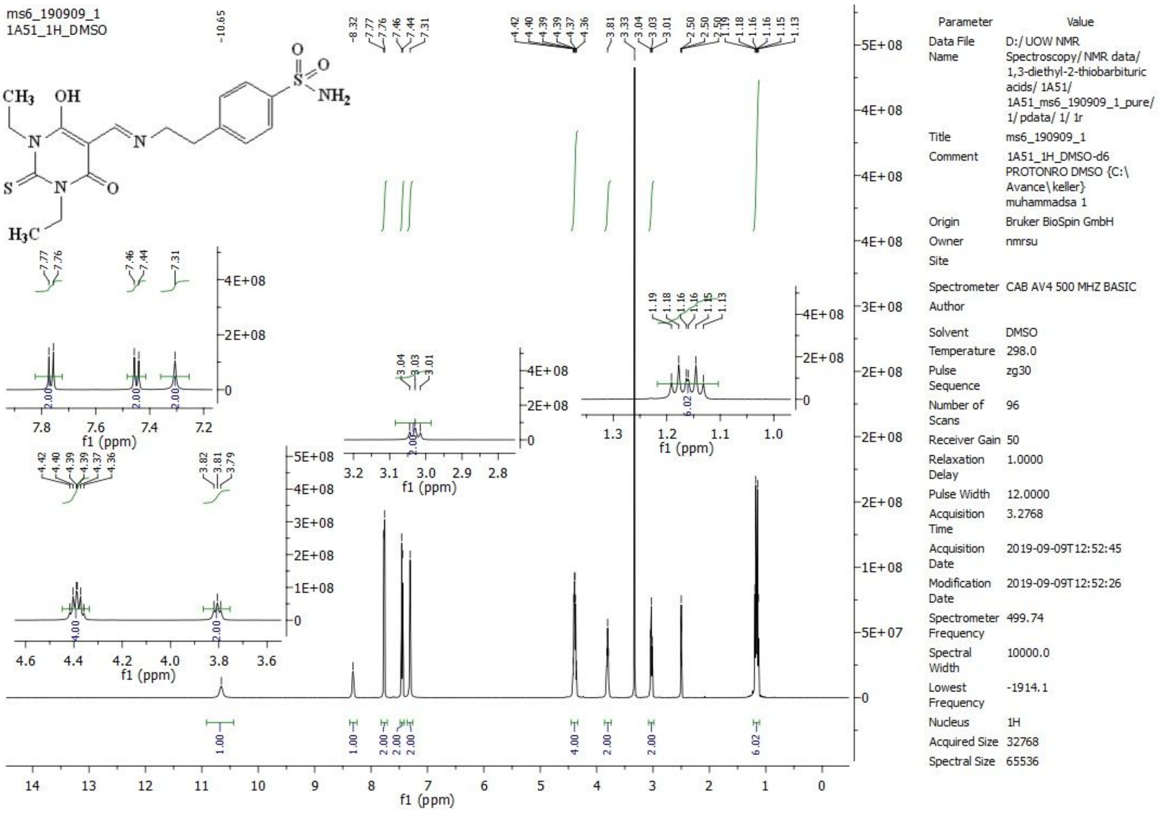

**Figure S9: $^{1}$H NMR Spectrum of compound A2**

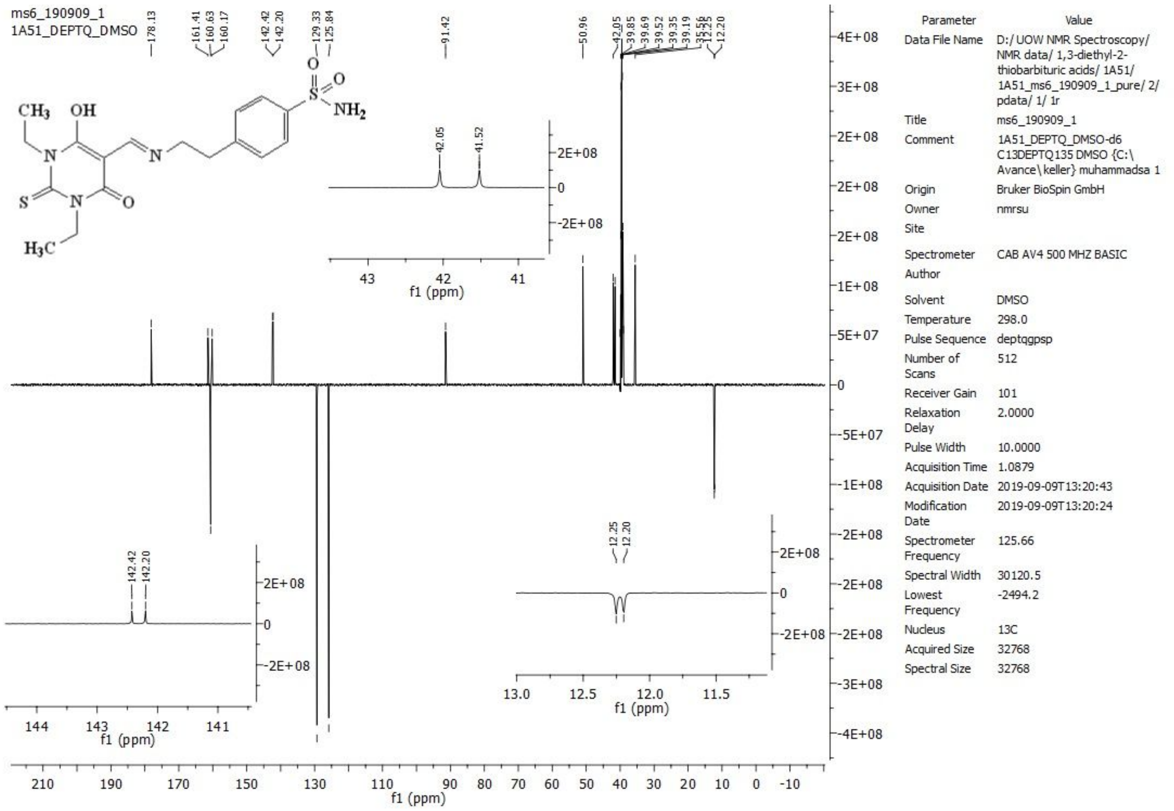

**Figure S10: $^{13}$C NMR (DEPTQ) Spectrum of compound A2**

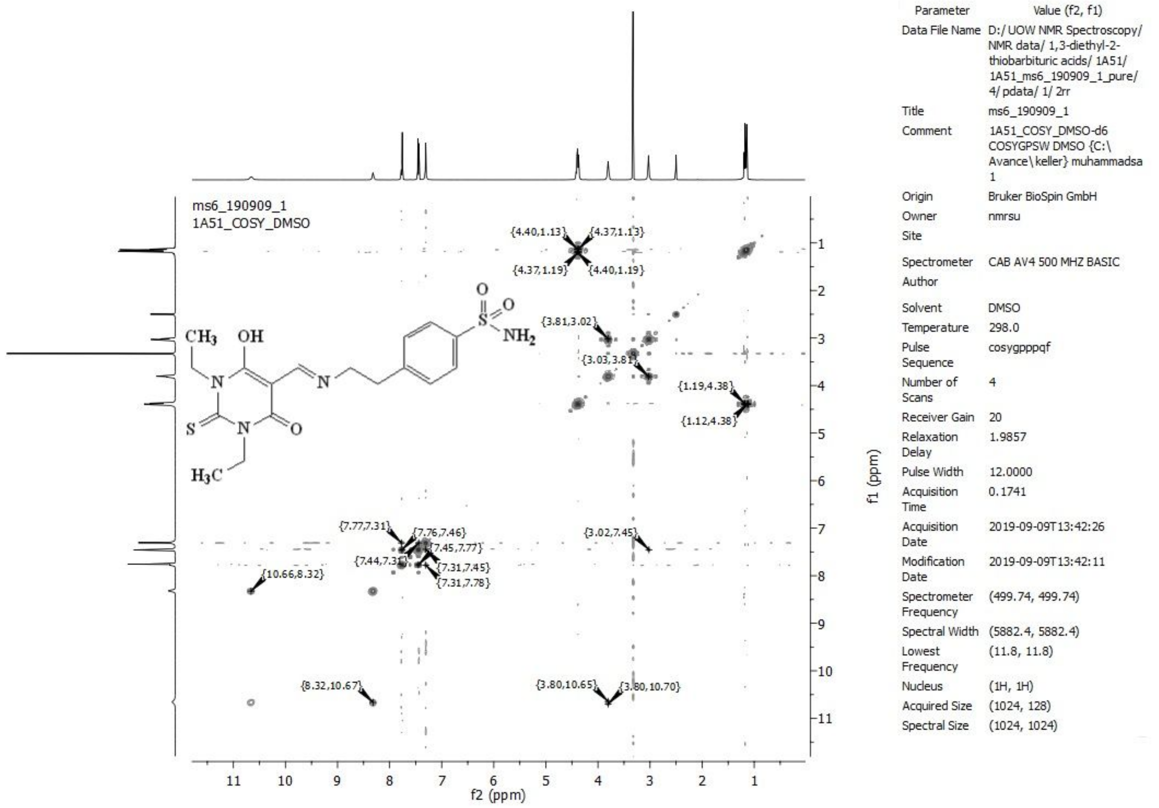

**Figure S11: COSY Spectrum of compound A2**

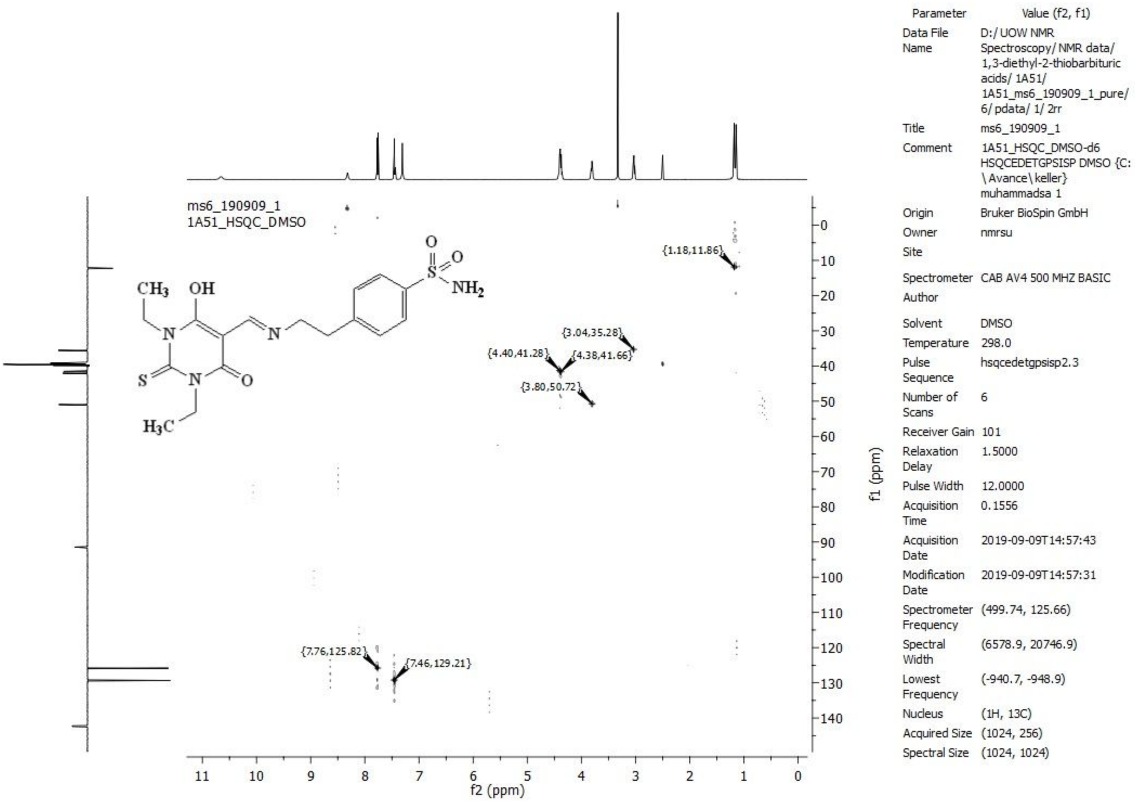


**Figure S12: HSQC Spectrum of compound A2**

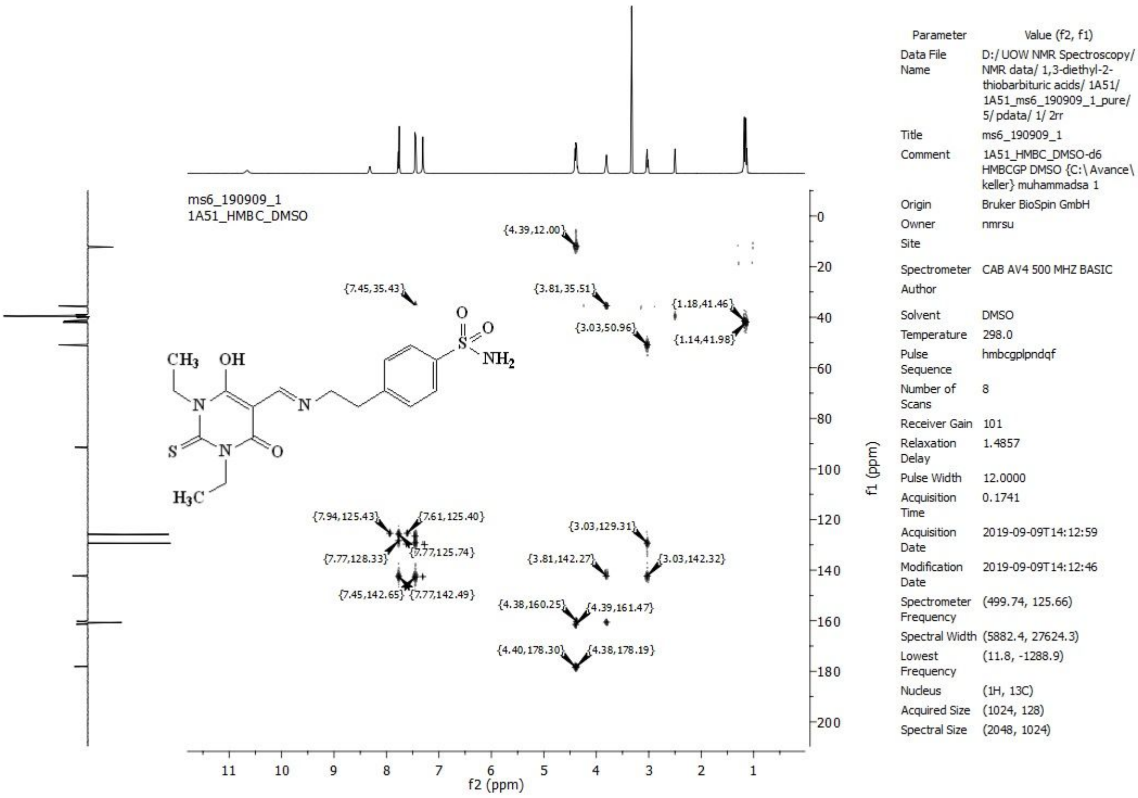


**Figure S13: HMBC Spectrum of compound A2**

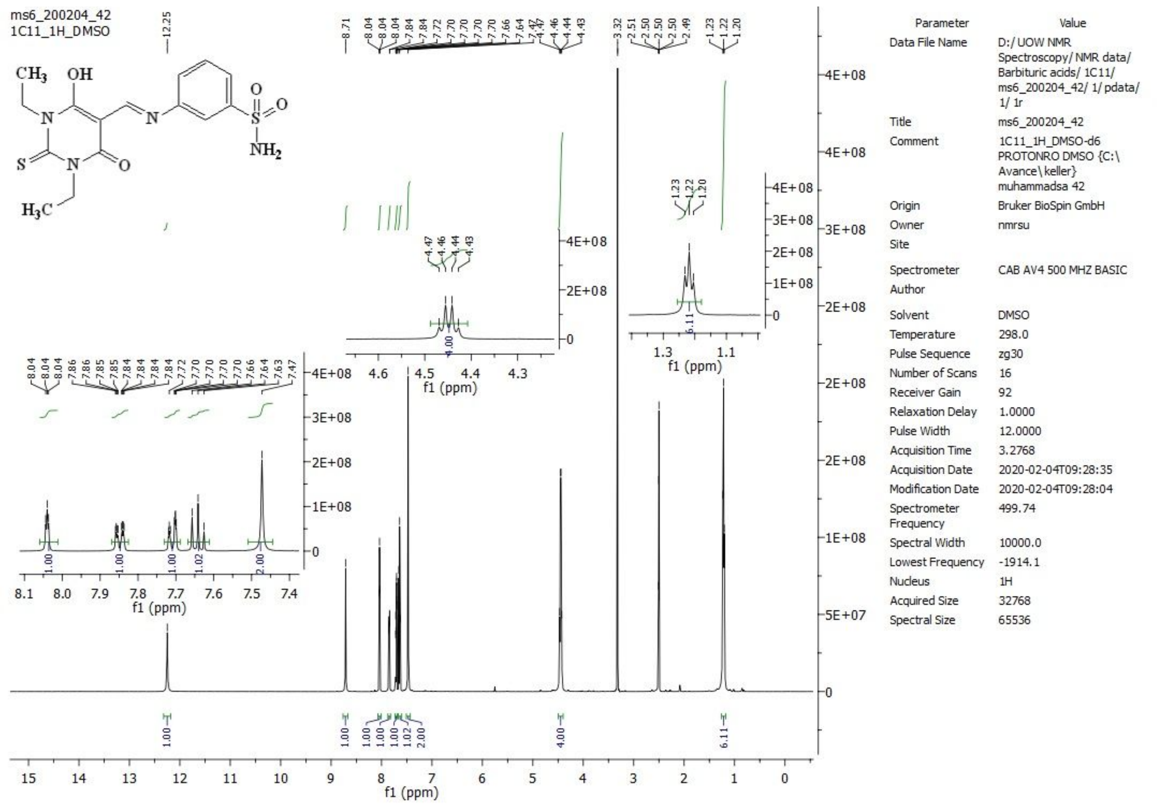

**Figure S14: $^{1}$H NMR Spectrum of compound A3**

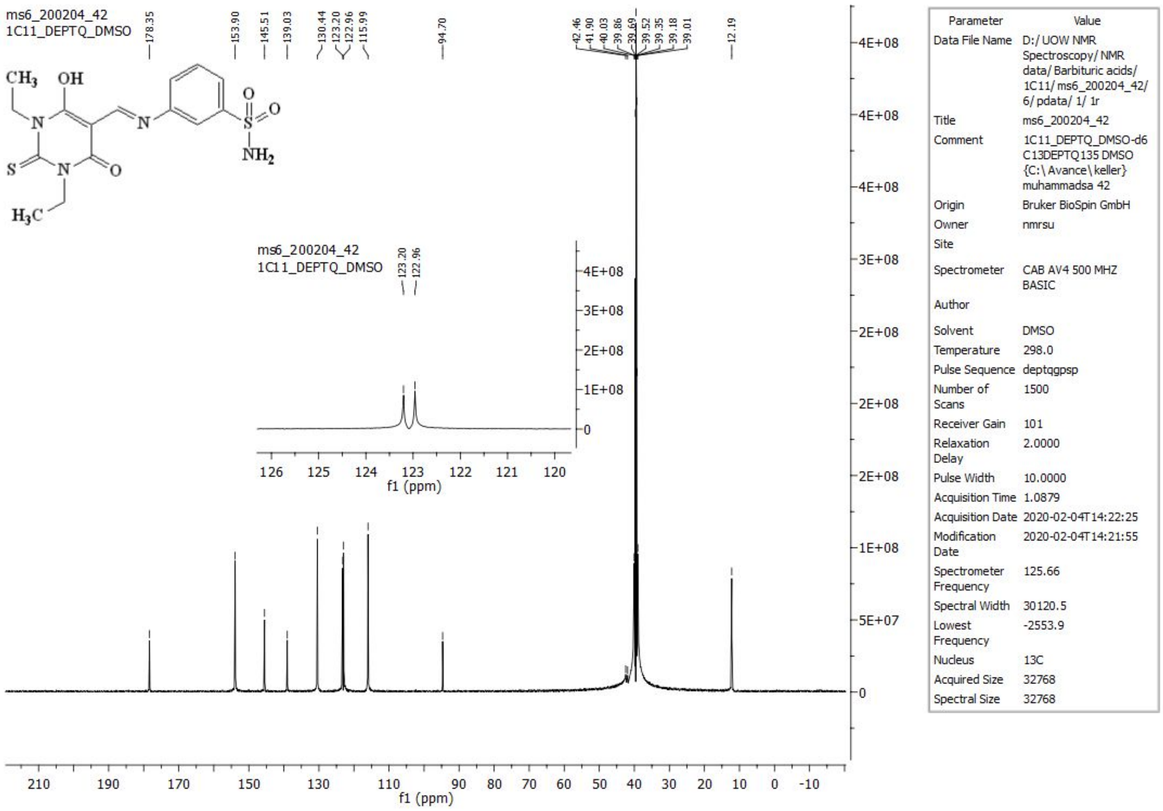

**Figure S15: $^{13}$C NMR Spectrum of compound A3**

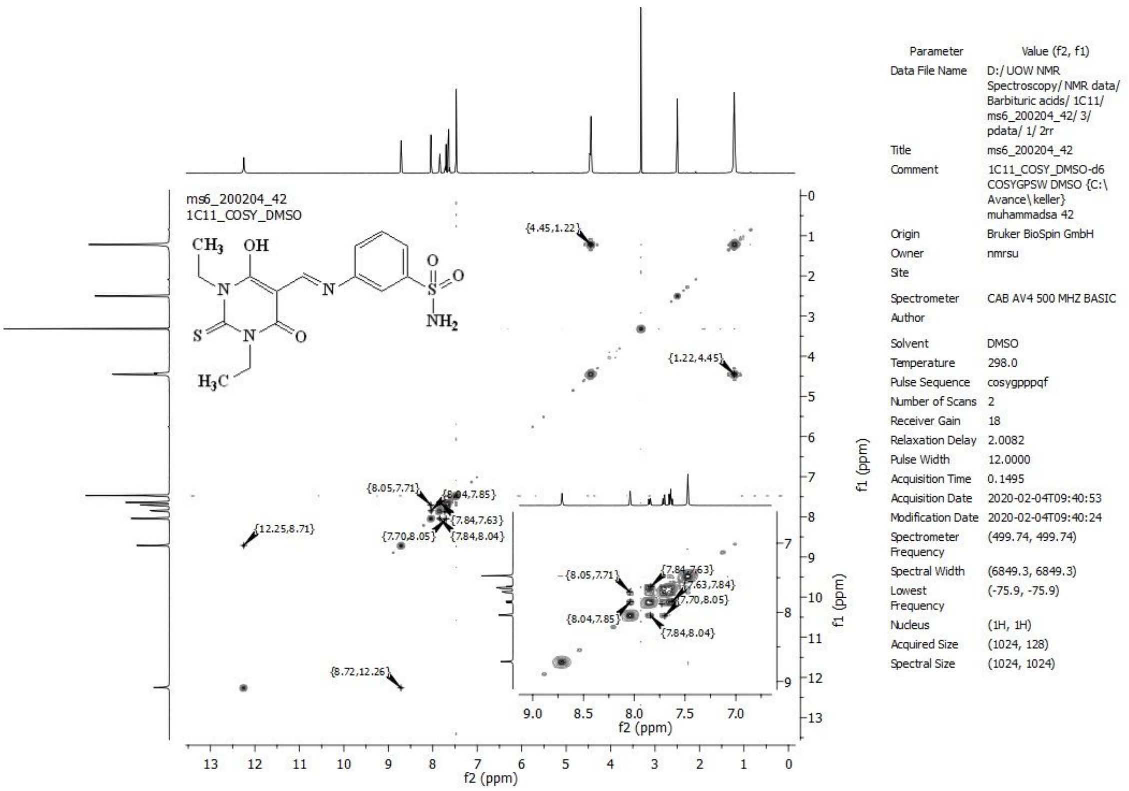


**Figure S16: COSY Spectrum of compound A3**

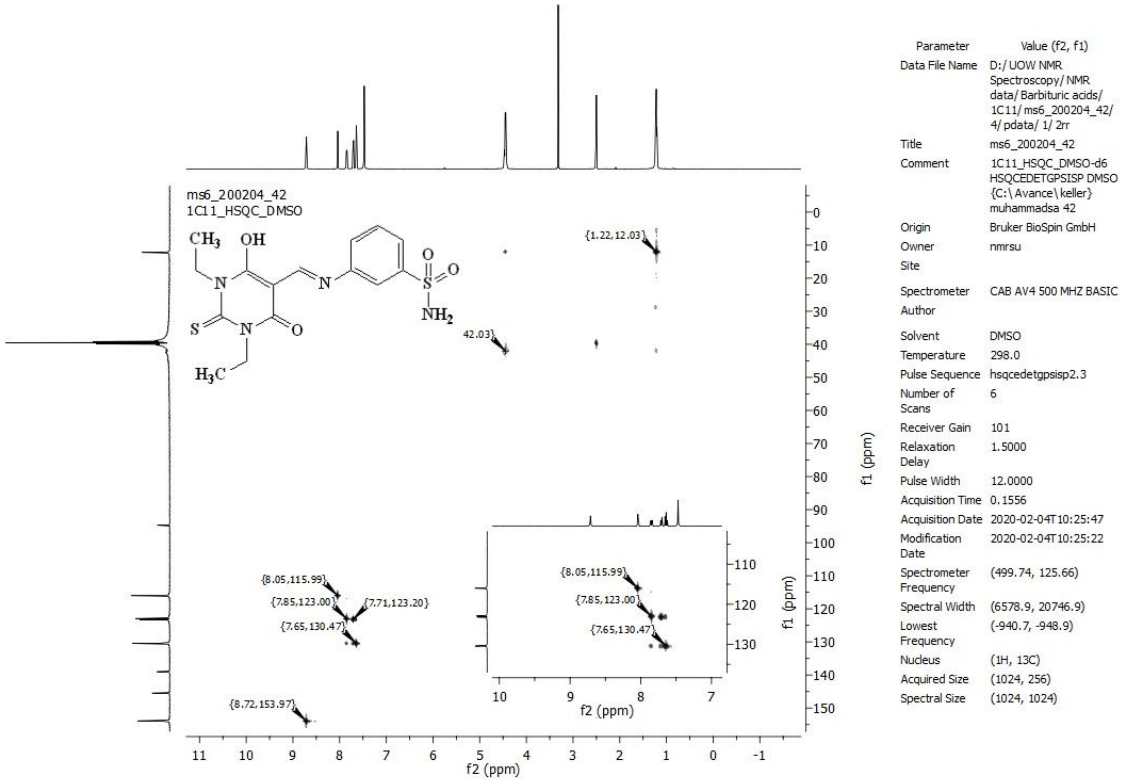


**Figure S17: HSQC Spectrum of compound A3**

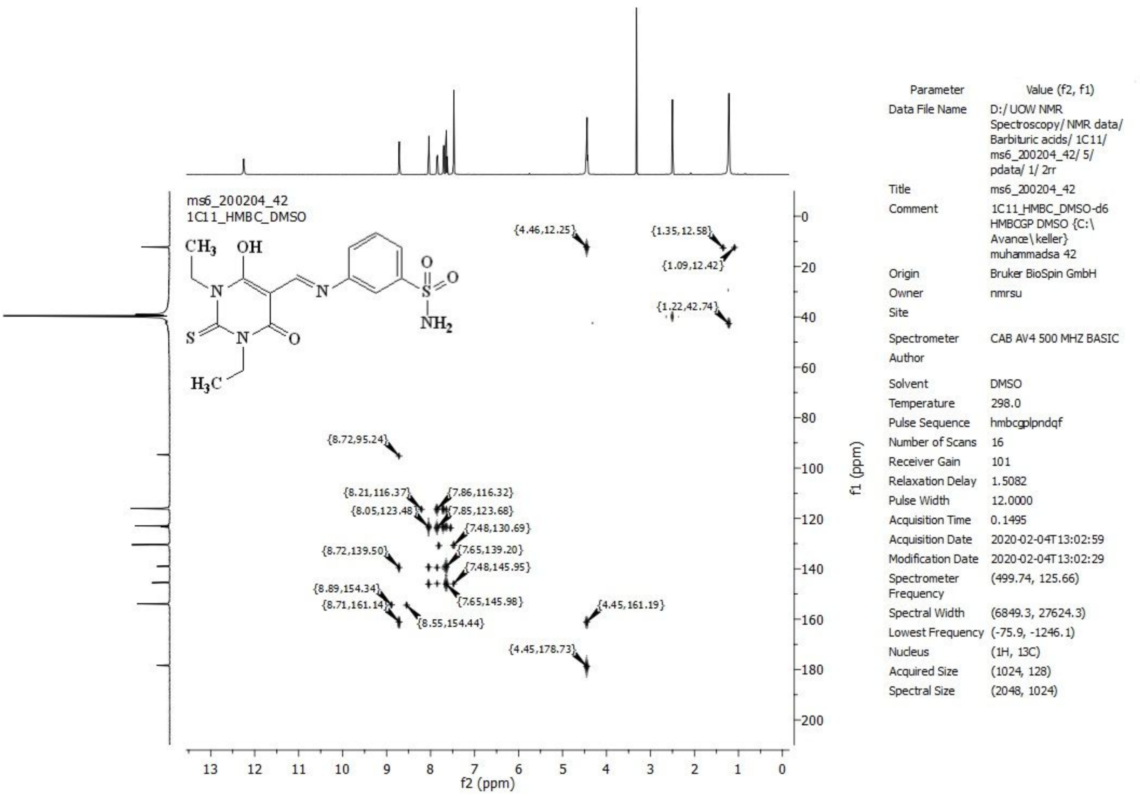


**Figure S18: HMBC Spectrum of compound A3**

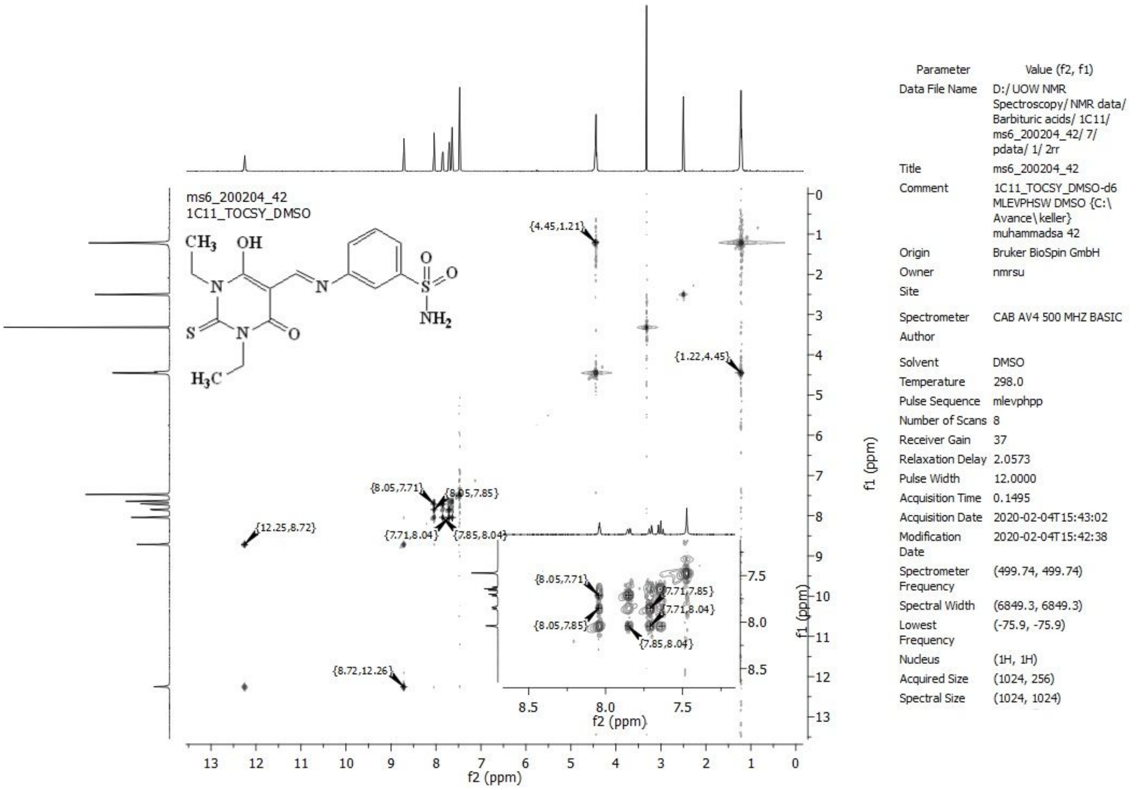


**Figure S19: TOCSY Spectrum of compound A3**

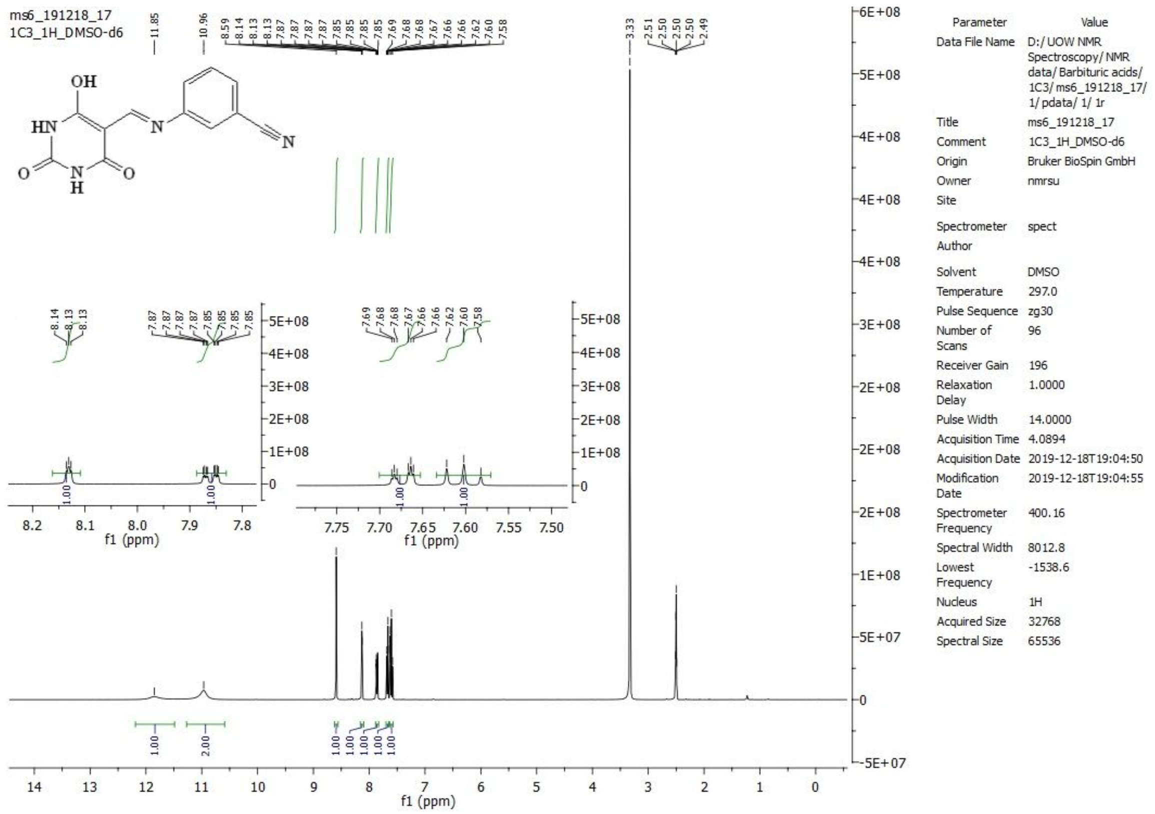


**Figure S20: $^{1}$H NMR Spectrum of compound A4**

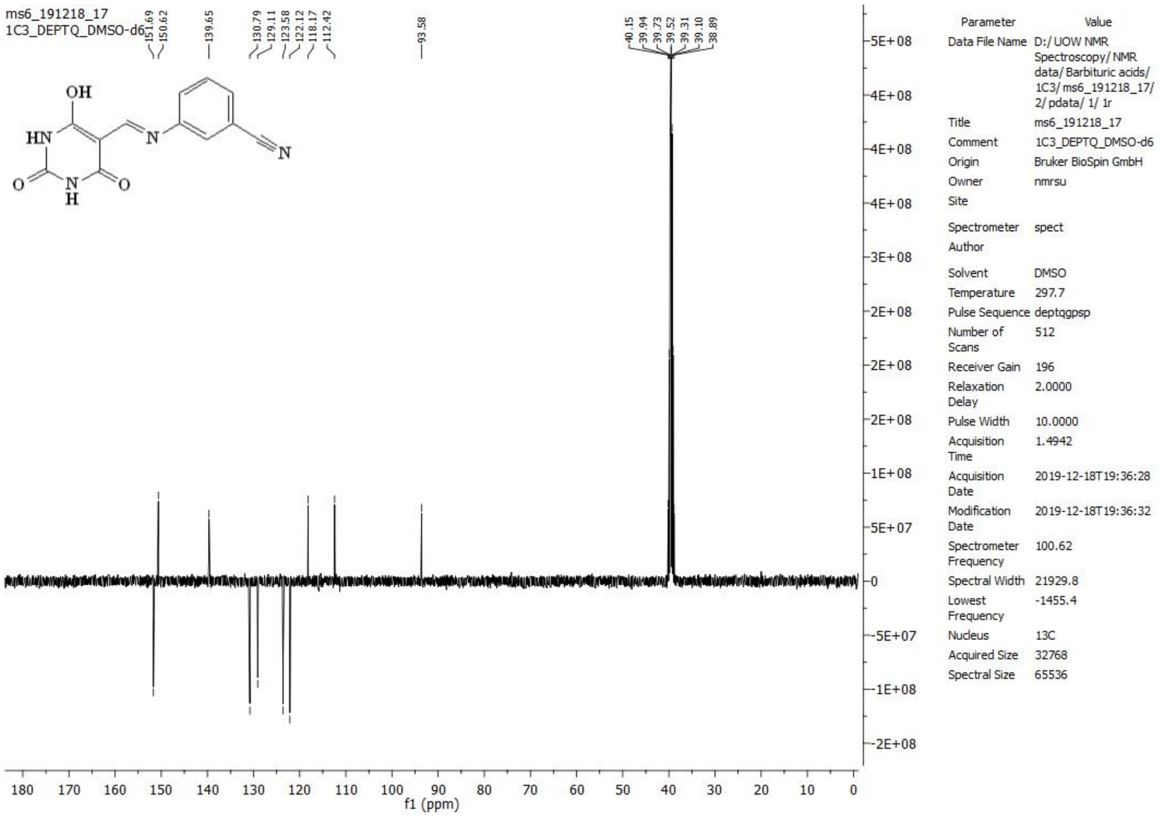


**Figure S21: $^{13}$C NMR (DEPTQ) Spectrum of compound A4**

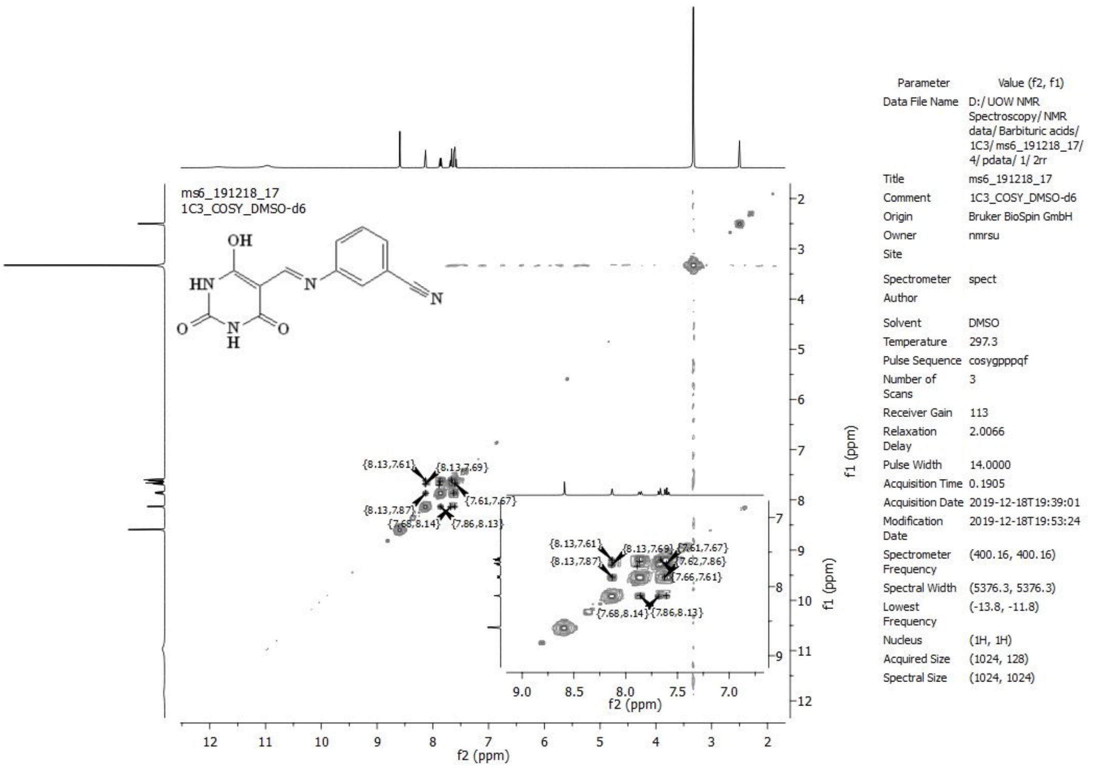


**Figure S22: COSY Spectrum of compound A4**

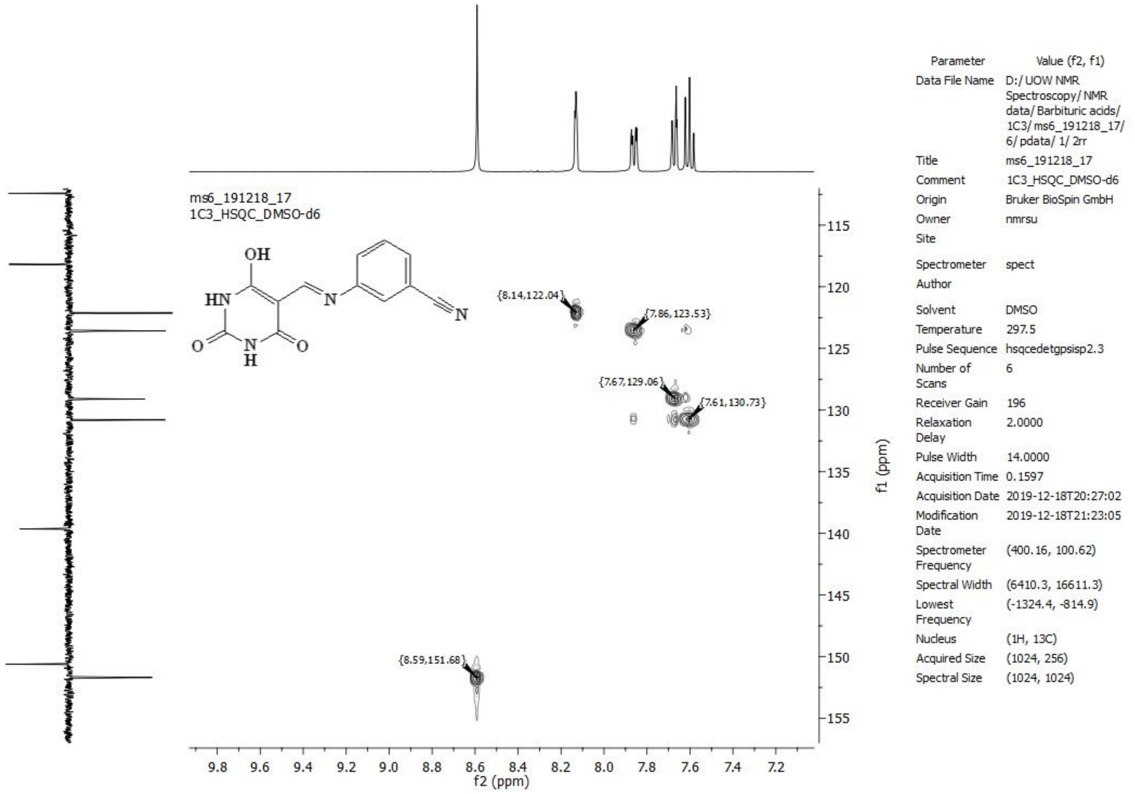


**Figure S23: HSQC Spectrum of compound A4**

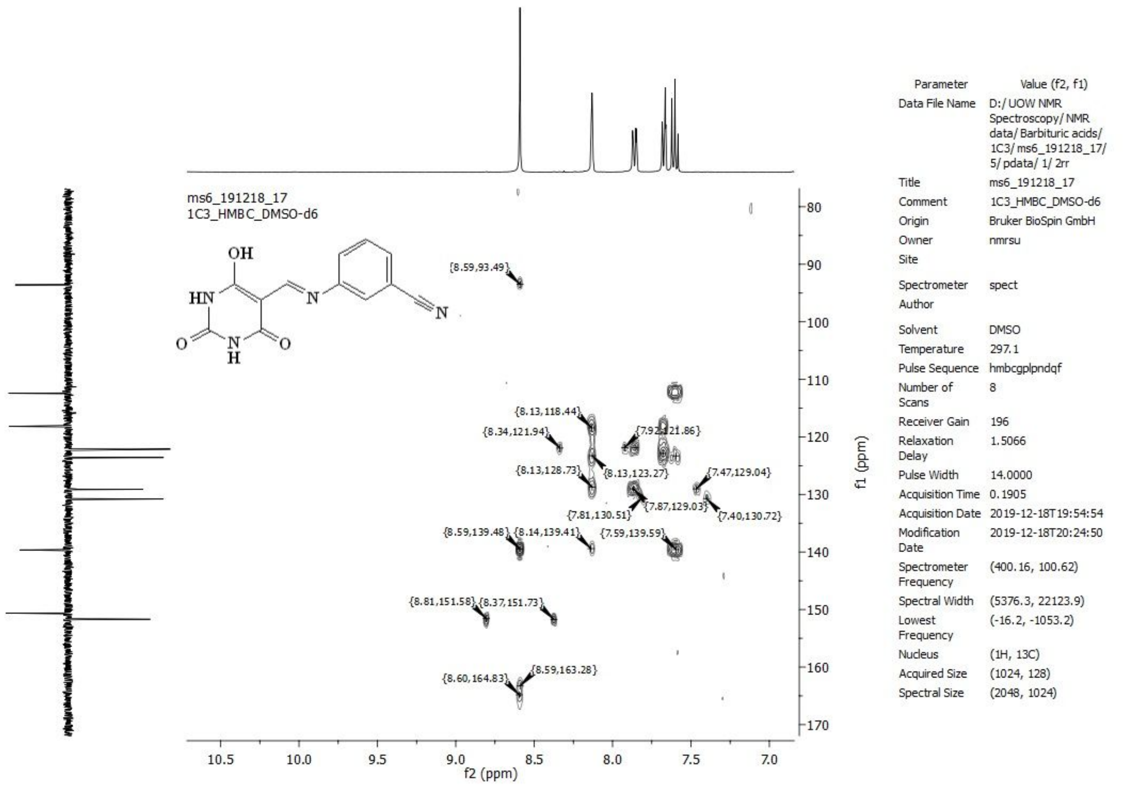


**Figure S24: HMBC Spectrum of compound A4**

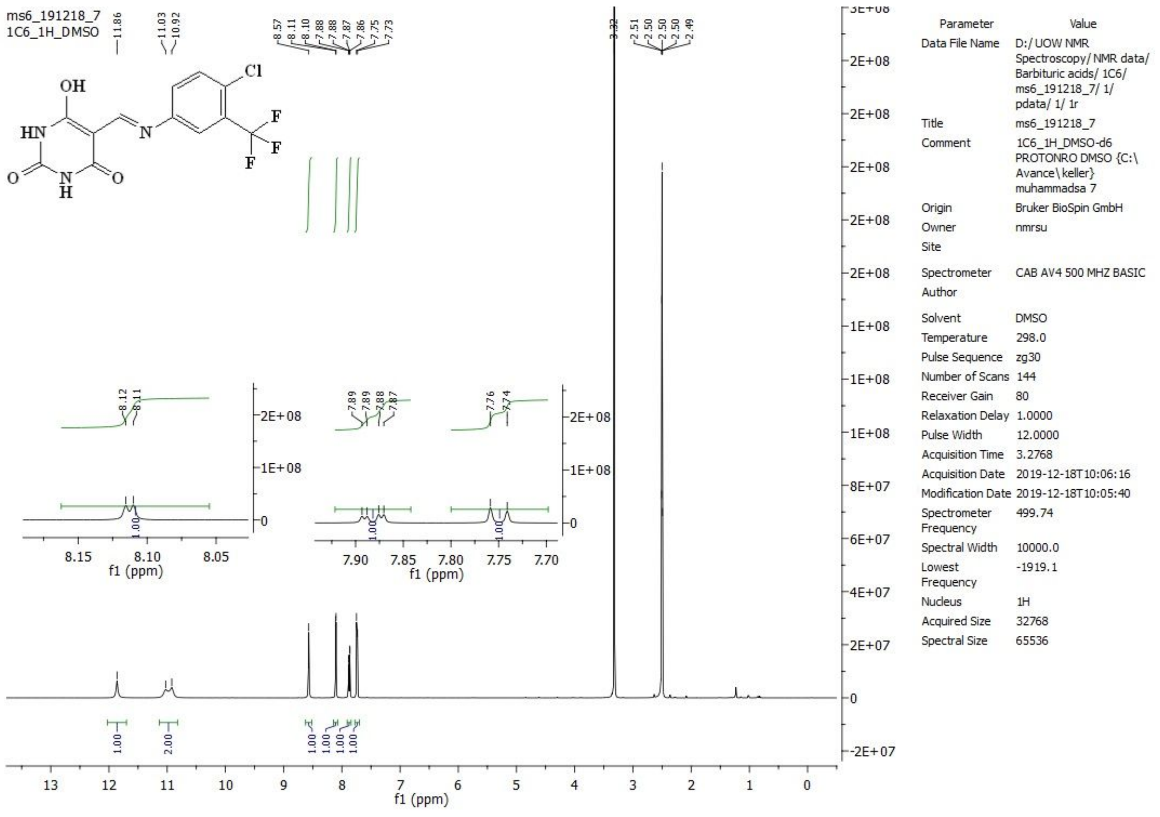


**Figure S25: $^1$H NMR Spectrum of compound A5**

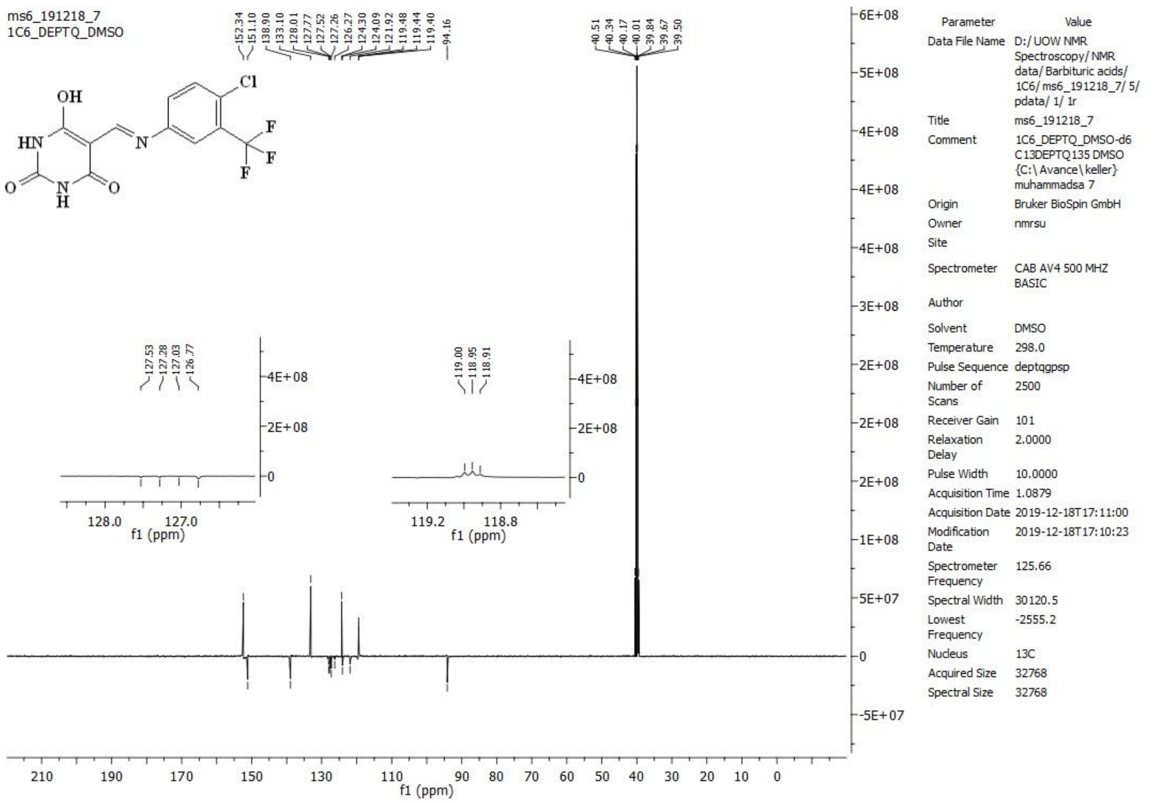


**Figure S26: $^{13}$C NMR (DEPTQ) Spectrum of compound A5**

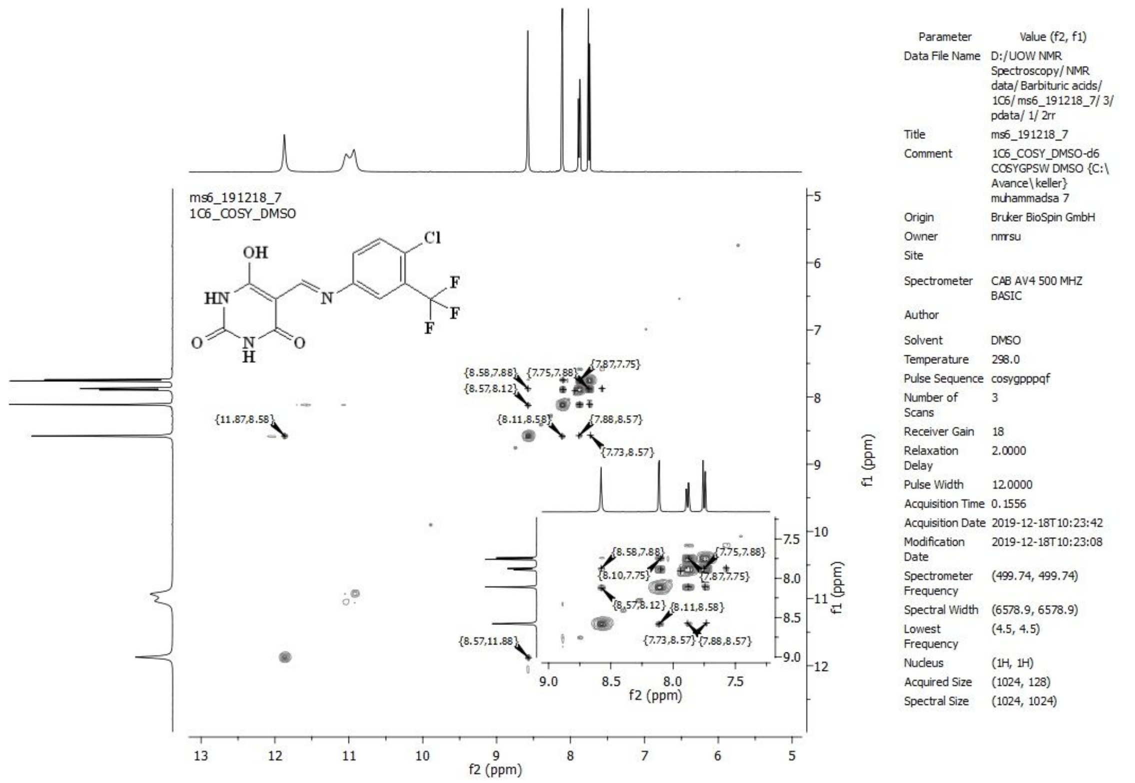


**Figure S27: COSY Spectrum of compound A5**

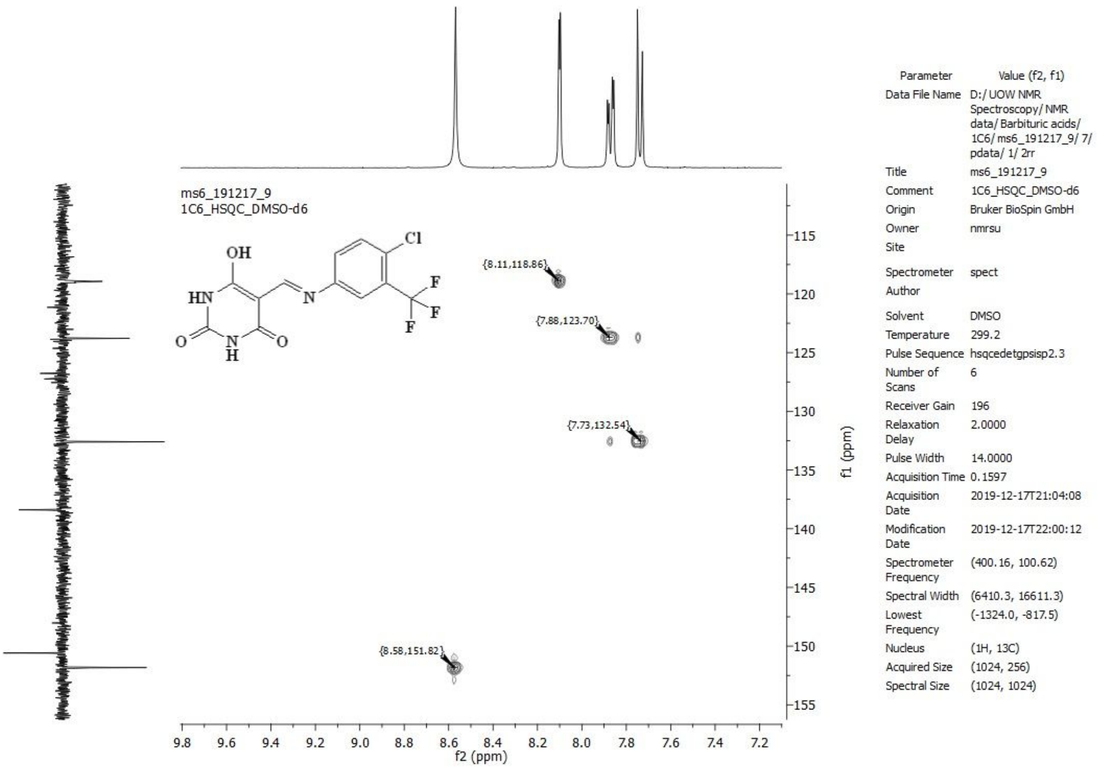


**Figure S28: HSQC Spectrum of compound A5**

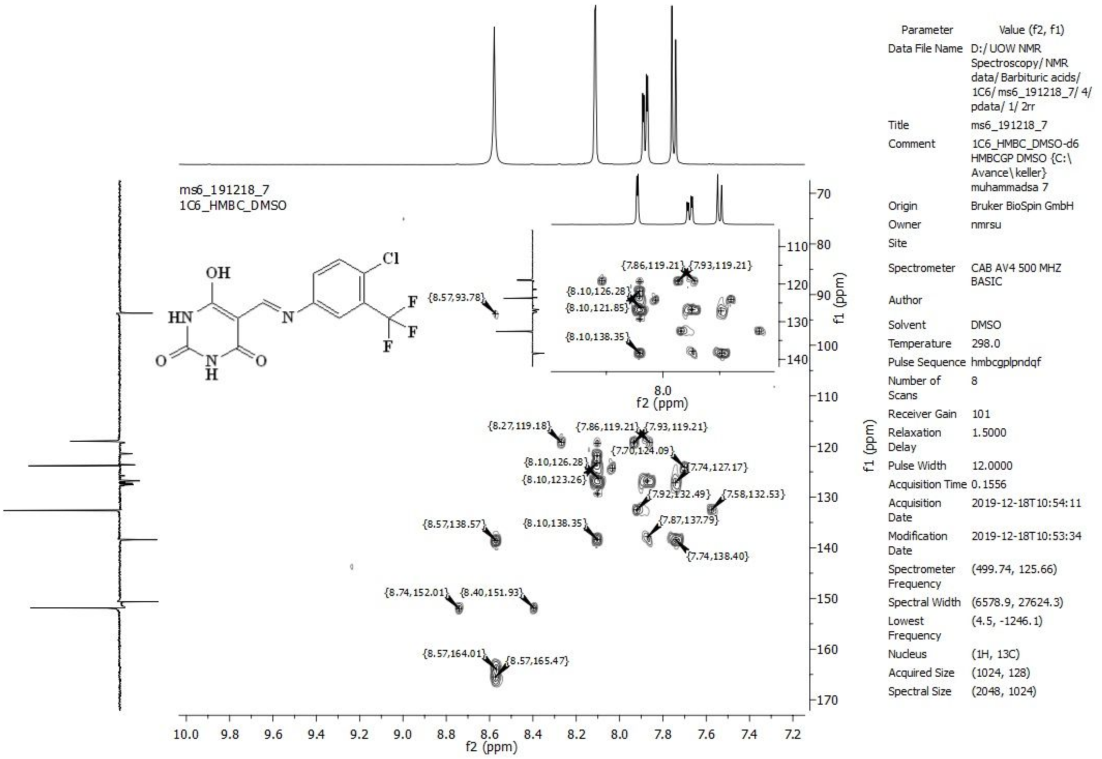


**Figure S29: HMBC Spectrum of compound A5**

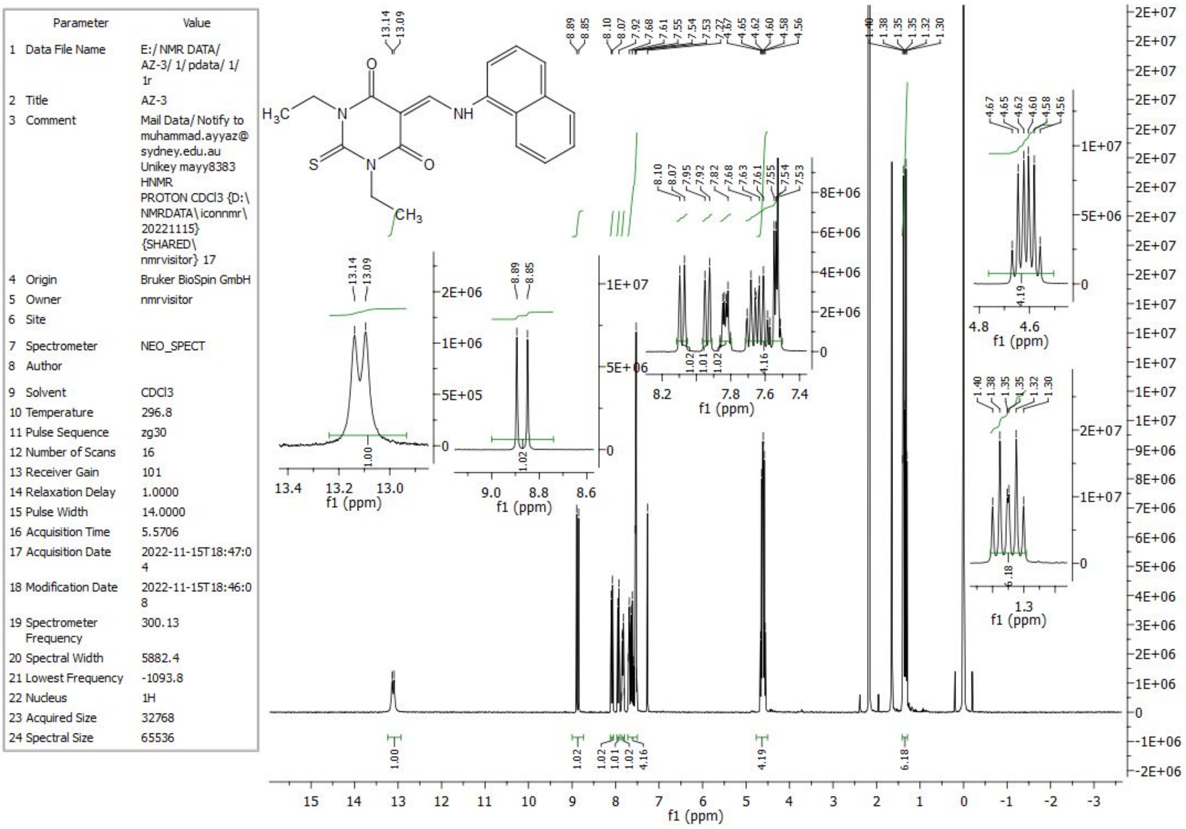


**Figure S30: ¹H NMR Spectrum of compound A6**

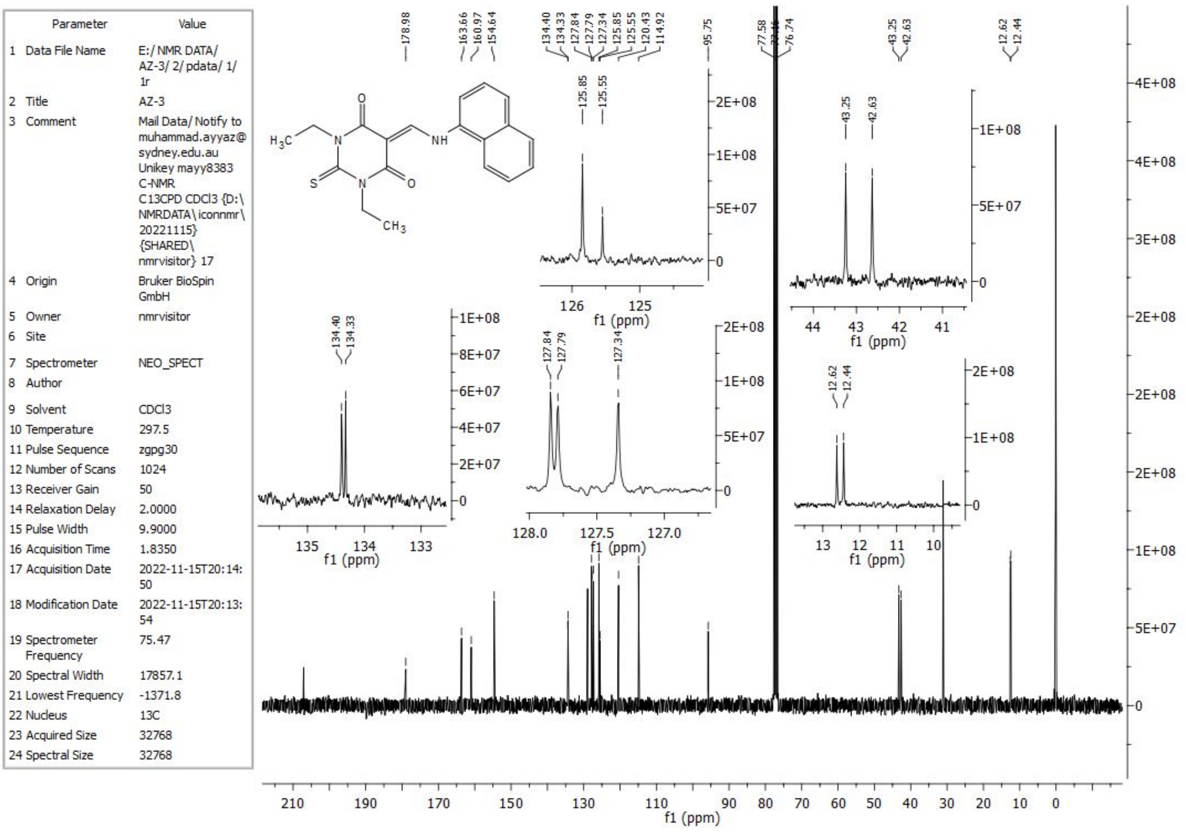


**Figure S31: ¹³C NMR Spectrum of compound A6**

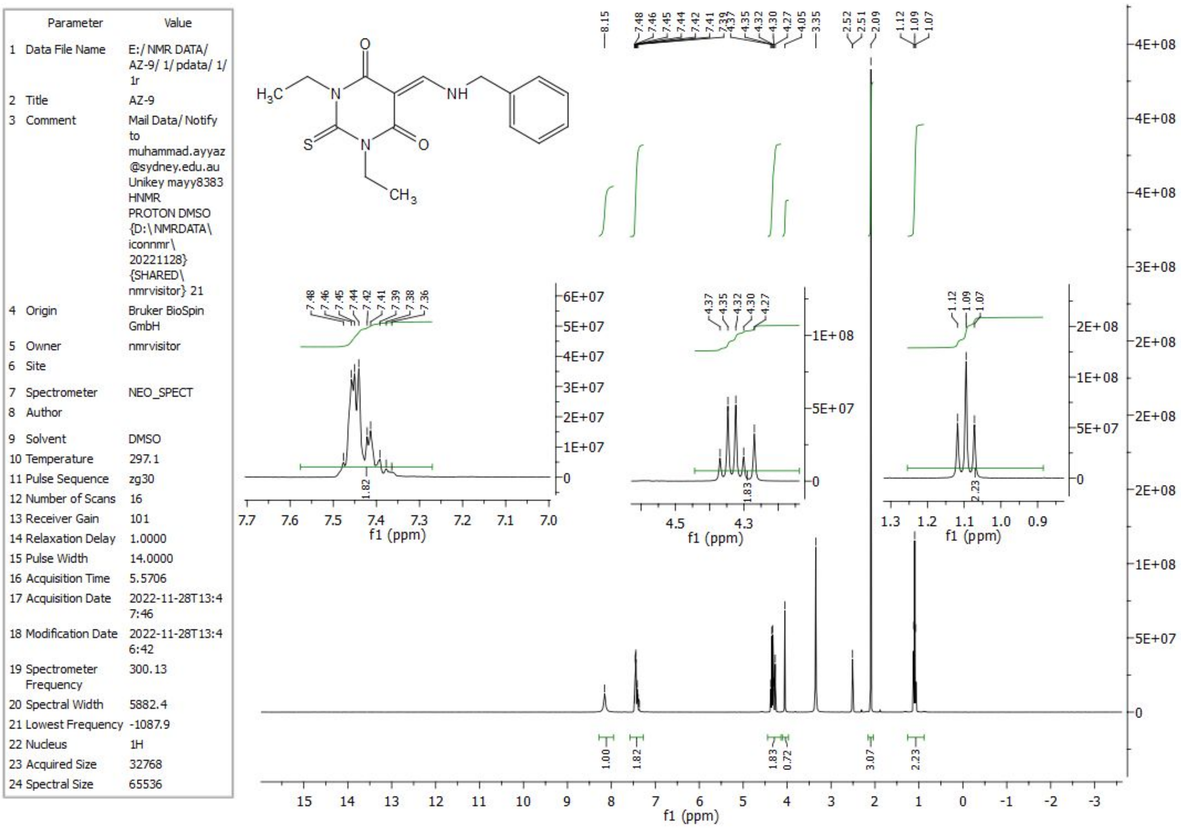


**Figure S32: $^{1}$H NMR Spectrum of compound A7**

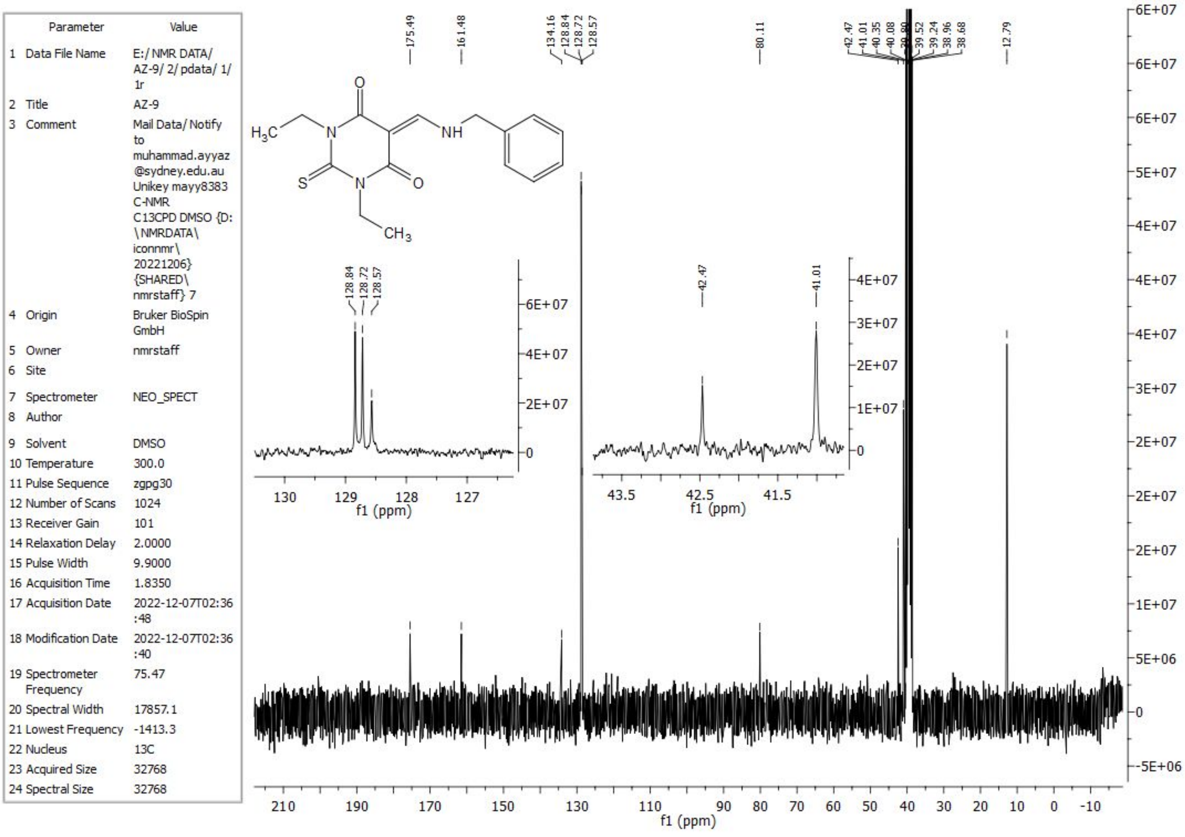


**Figure S33: $^{13}$C NMR Spectrum of compound A7**

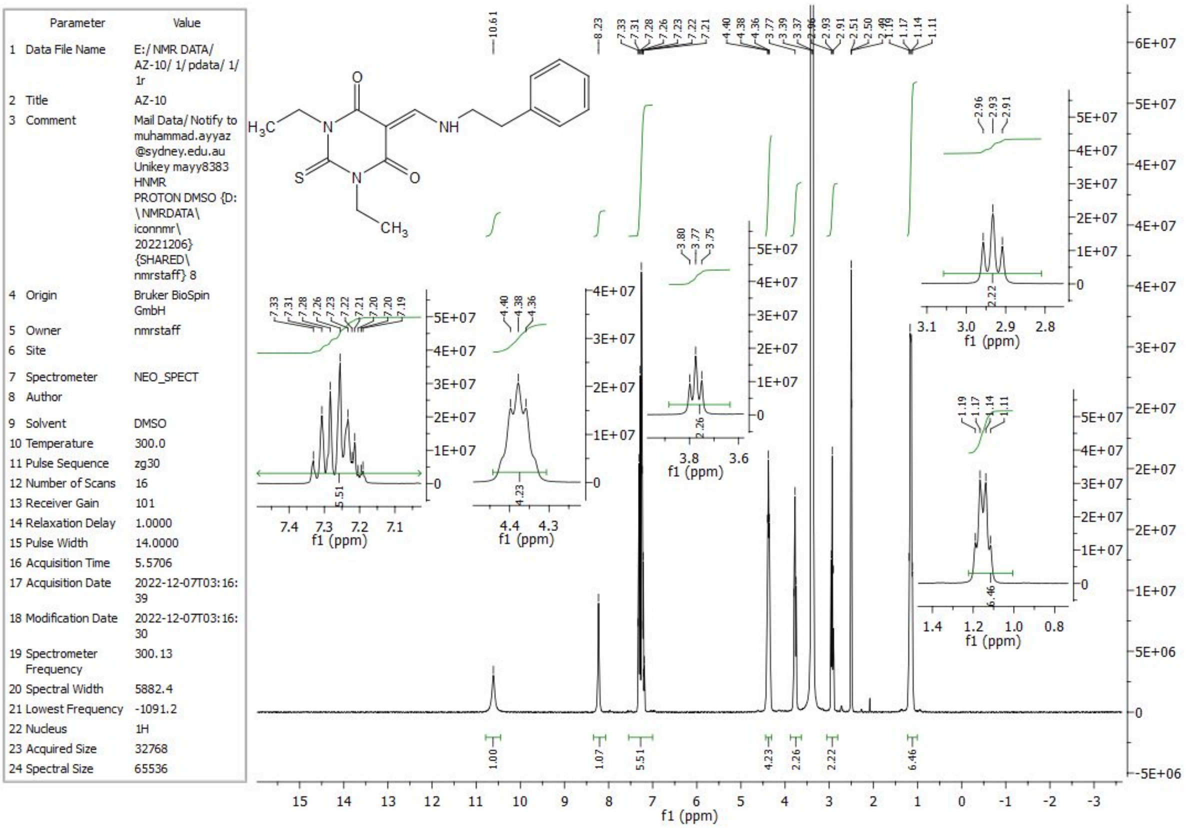


Figure S34: ¹H NMR Spectrum of compound A8

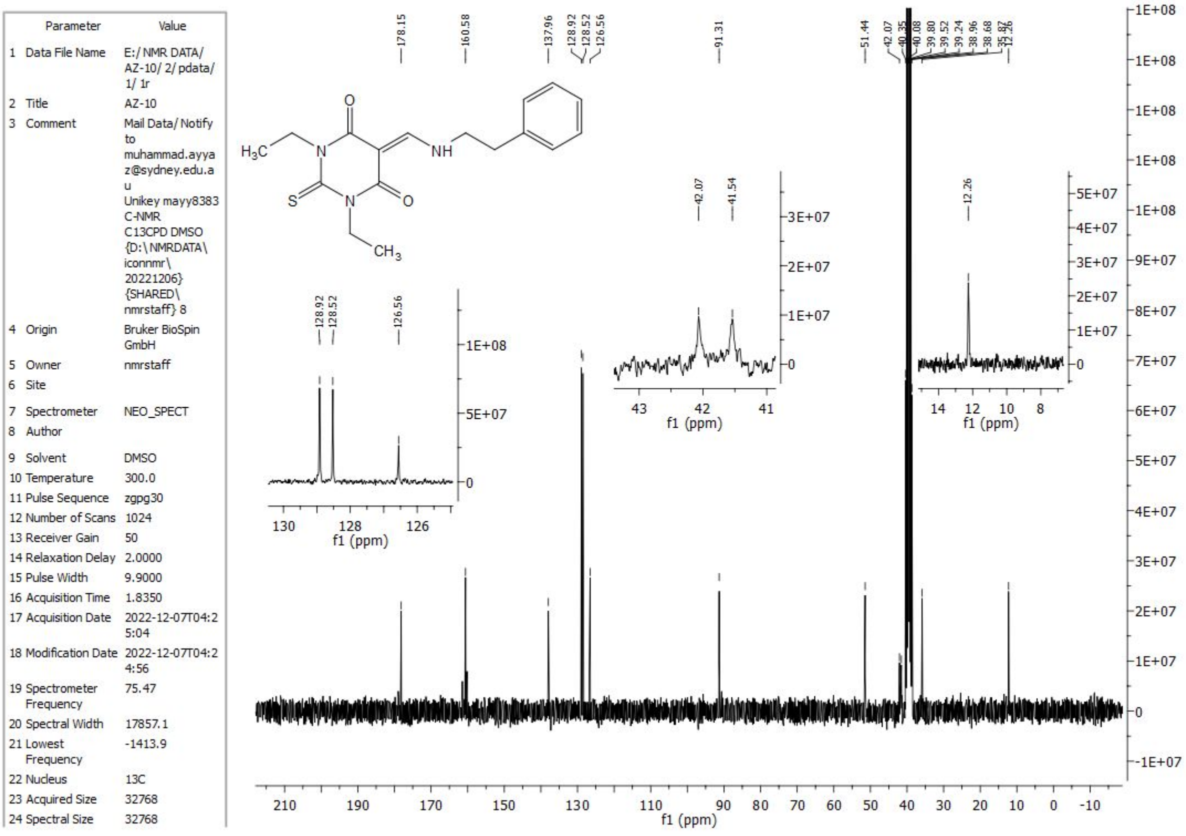


Figure S35: ¹³C NMR Spectrum of compound A8

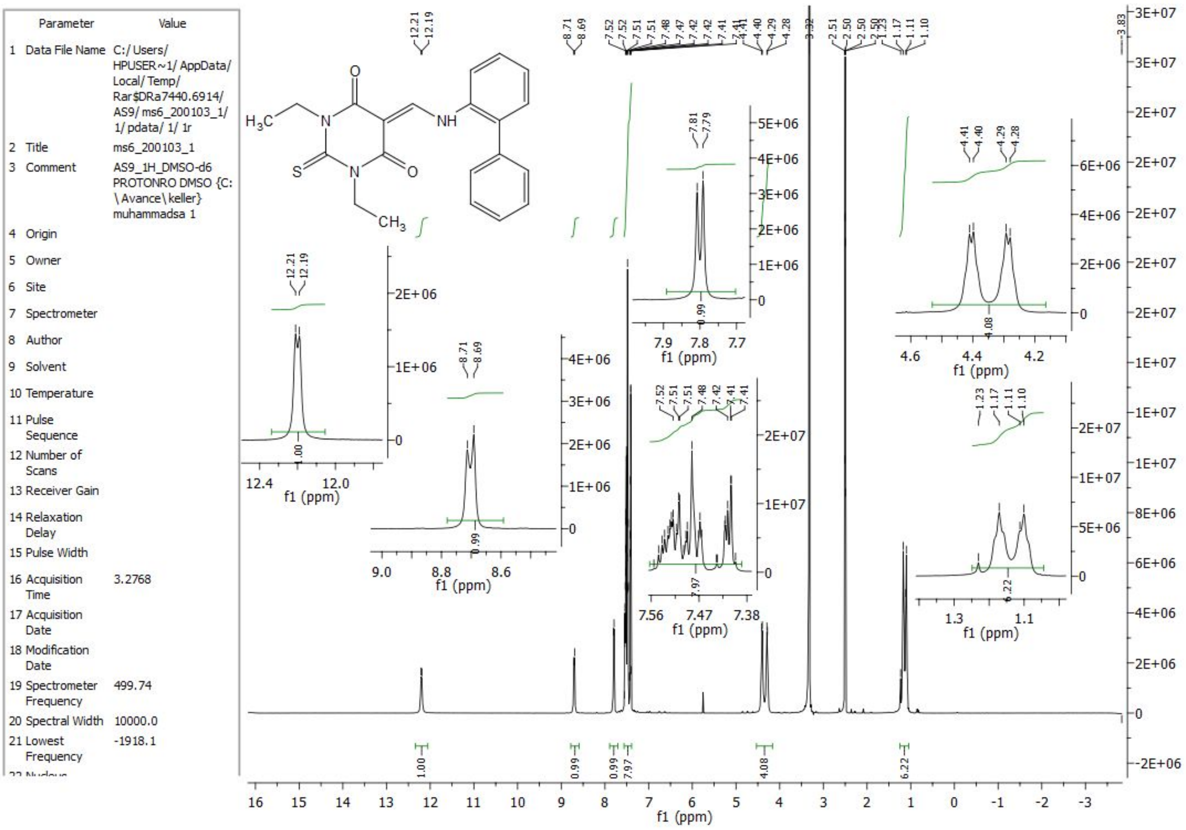

**Figure S36: $^{1}$H NMR Spectrum of compound A9**

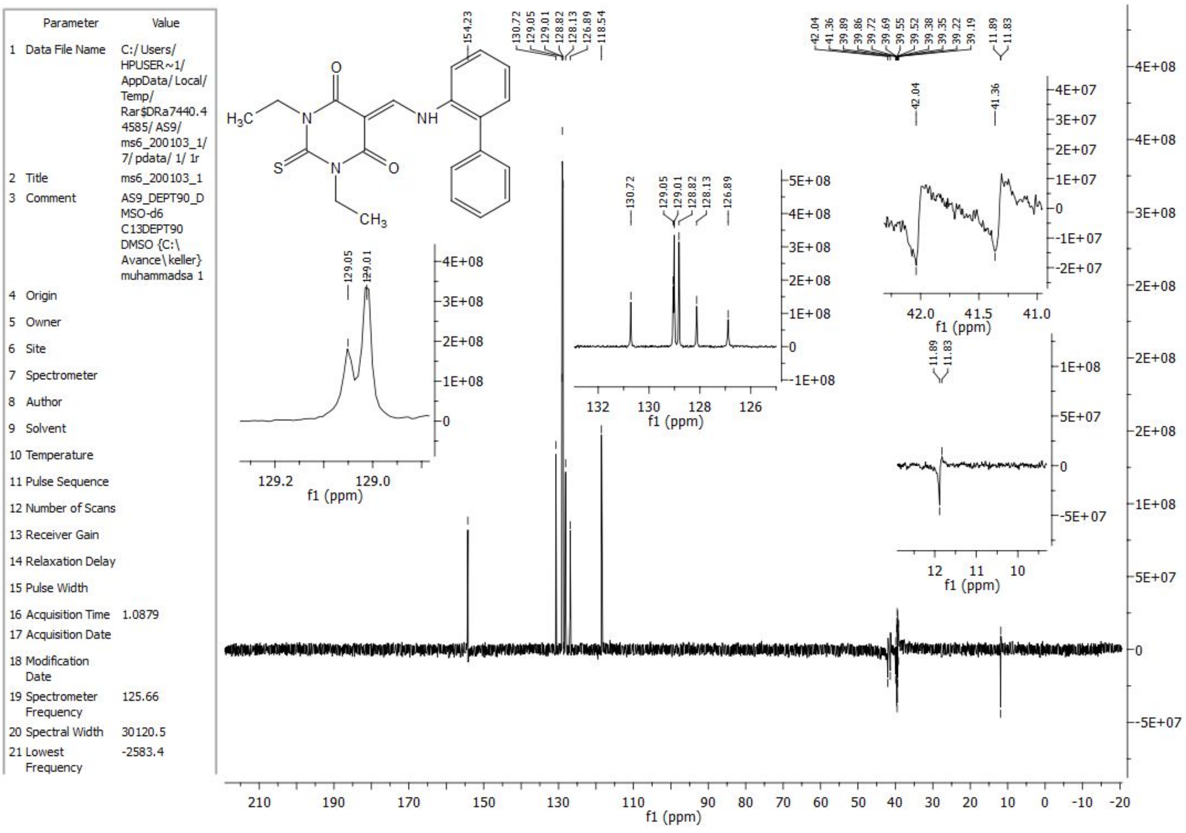

**Figure S37: $^{13}$C NMR Spectrum of compound A9**

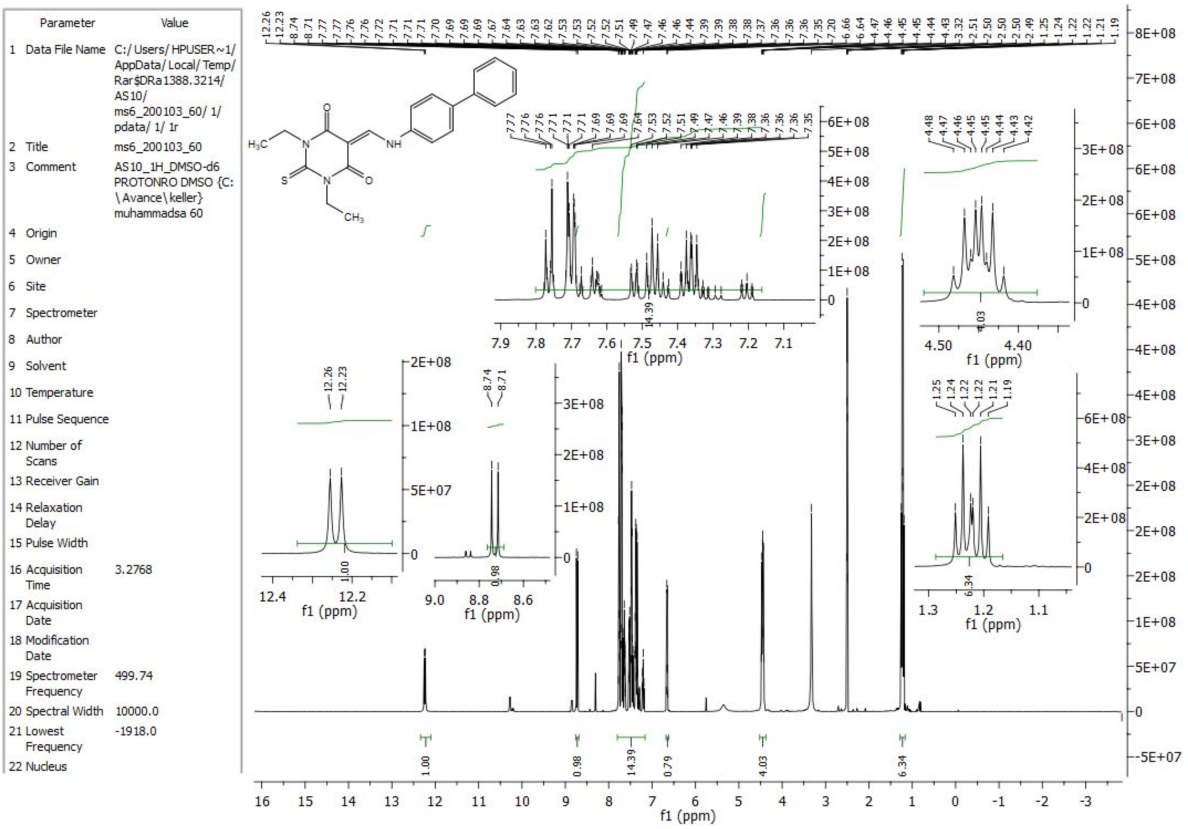


**Figure S38: $^{1}$H NMR Spectrum of compound A10**

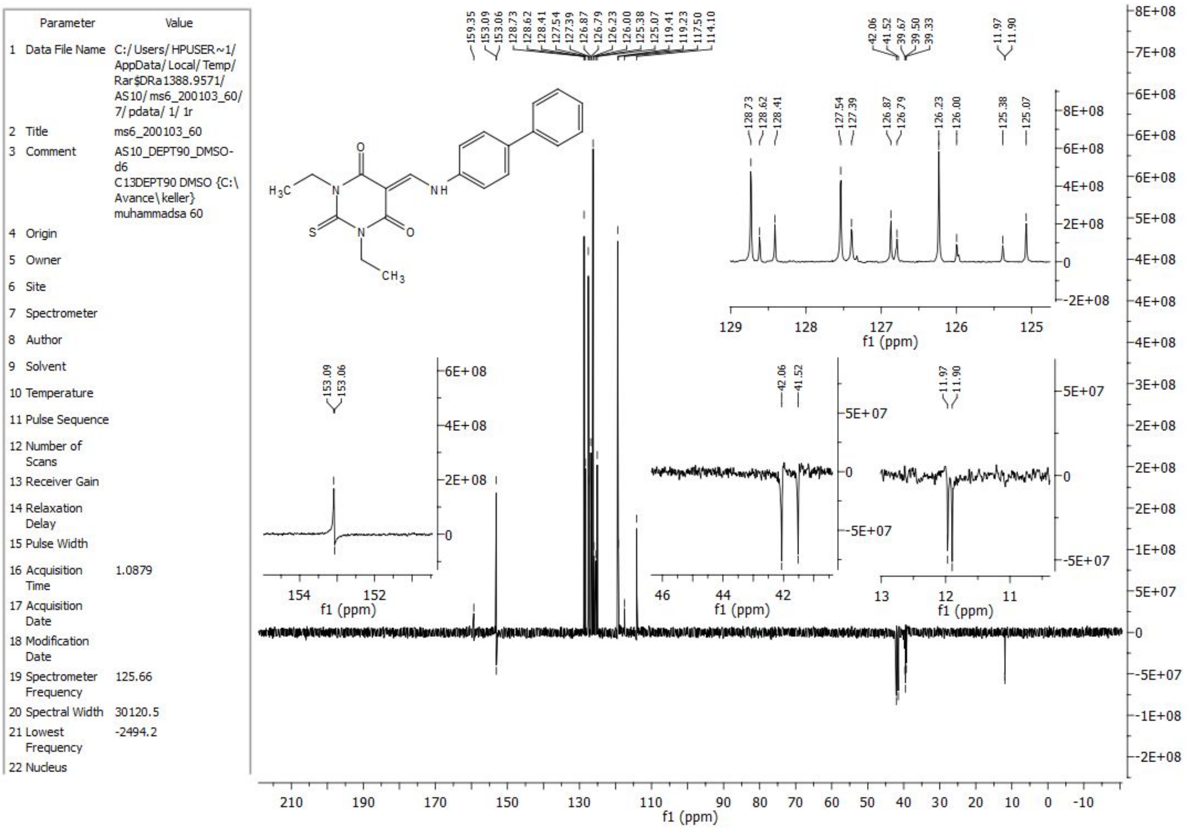


**Figure S39: $^{13}$C NMR Spectrum of compound A10**

**Table S4:** Calculations of physicochemical and physicokinetic properties as well as drug likeness score of compounds **A1–A5** using ACD chemsketch, ChemDraw and molsoft programs.

| Structure | 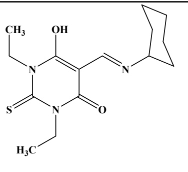 | 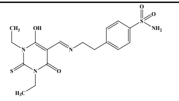 | 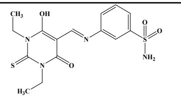 | 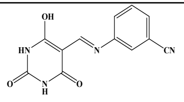 | 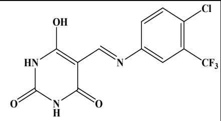 |
|---|---|---|---|---|---|
| Formula | $C_{15}H_{23}N_3O_2S$ | $C_{17}H_{22}N_4O_4S_2$ | $C_{15}H_{18}N_4O_4S_2$ | $C_{12}H_8N_4O_3$ | $C_{12}H_7ClF_3N_3O_3$ |
| Exact mass | 309.1511 | 410.1082 | 382.0769 | 256.0596 | 333.0128 |
| Mol. Wt. | 309.4280 | 410.5070 | 382.4530 | 256.2210 | 333.6512 |
| m/z | 309.1511 (100%) 310.1545 (16.2%) 311.1469 (4.5%) | 410.1082 (100%) 411.1116 | 382.0769 (100%) 382.0803 | 256.0596 (100%) 257.0630 | 333.0128 (100%) 334.0162 (13%) 335.0099 (32%) |
| Elem. Anal. | C, 58.23; H, 7.49; N, 13.58; O, 10.34; S, 10.36 | C, 49.74; H, 5.40; N, 13.65; O, 15.59; S, 15.62 | C, 47.11; H, 4.74; N, 14.65; O, 16.73; S, 16.77 | C, 56.25; H, 3.15; N, 21.87; O, 18.73 | C, 43.20; H, 2.11; Cl, 10.62; F, 17.08; N, 12.59; O, 14.39 |
| B.P (K) | 1010.21 | 1313.3 | 1267.54 | 1001.1 | 936.01 |
| $T_c$ (K) | - | - | - | 1041.1 | 994.88 |
| $P_c$ (Bar) | - | - | - | 31 | 26.6 |
| LogP | 2.17 | 1.38 | 1.03 | 0.18 | 1.63 |
| MR($cm^3$/mol) | 86.20±0.5 | 107.08±0.5 | 97.87±0.5 | 66.38±0.5 | 69.21±0.5 |
| MV($cm^3$/mol) | 244.8±7.0 | 294.0±7.0 | 261.9±7.0 | 171.5±7.0 | 198.1±7.0 |
| Parachor($cm^3$) | 637.0±8.0 | 802.5±8.0 | 725.3±8.0 | 491.5±8.0 | 528.6±8.0 |
| Refract. Index | 1.621±0.05 | 1.648±0.05 | 1.670±0.05 | 1.700±0.05 | 1.615±0.05 |

| | | | | | |
|---|---|---|---|---|---|
| Surf. Tension (dynes/cm) | 45.7±7.0 | 55.4±7.0 | 58.8±7.0 | 67.3±7.0 | 50.6±7.0 |
| Dens. (g/cm$^3$) | 1.26±0.1 | 1.39±0.1 | 1.46±0.1 | 1.49±0.1 | 1.68±0.1 |
| Polarizability (10-24cm$^3$) | 34.17±0.5 | 42.45±0.5 | 38.79±0.5 | 26.31±0.5 | 27.42±0.5 |
| Henry's law | - | - | - | 2.43 | 2.43 |
| Heat of Form. (KJ/mol) | - | - | - | 44.84 | -744.33 |
| tPSA | 56.14 | 116.3 | 116.3 | 114.58 | 90.79 |
| CLogP | 2.685 | 0.764 | 0.2395 | 0.2903 | 2.2155 |
| CMR | 8.9696 | 11.0442 | 9.9646 | 6.4897 | 7.0137 |
| LogS | -3.571 | -3.9 | -3.534 | -2.309 | -3.96 |
| No. of HBA | 4 | 7 | 7 | 5 | 4 |
| No. of HBD | 1 | 3 | 3 | 3 | 3 |
| MolLogP | 2.69 | 0.93 | 0.65 | 0.05 | 1.90 |
| MolLogS | -2.62 | -2.17 | -2.03 | -2.17 | -3.05 |
| MolPSA (A$^2$) | 44.00 | 93.93 | 93.55 | 90.48 | 73.42 |
| MolVol (A$^3$) | 358.42 | 411.64 | 376.35 | 272.71 | 291.99 |
| pKa of most basic group | -1.77 | -3.53 | -4.23 | -4.99 | -4.99 |
| pKa of most acidic group | 6.01 | 6.32 | 5.41 | 3.19 | 3.19 |
| BBB score | 3.44 | 2.36 | 2.43 | 2.55 | 2.77 |
| Drug-likeness model score | 0.01 | -0.22 | -0.95 | -1.36 | -0.66 |

A1-4NOS

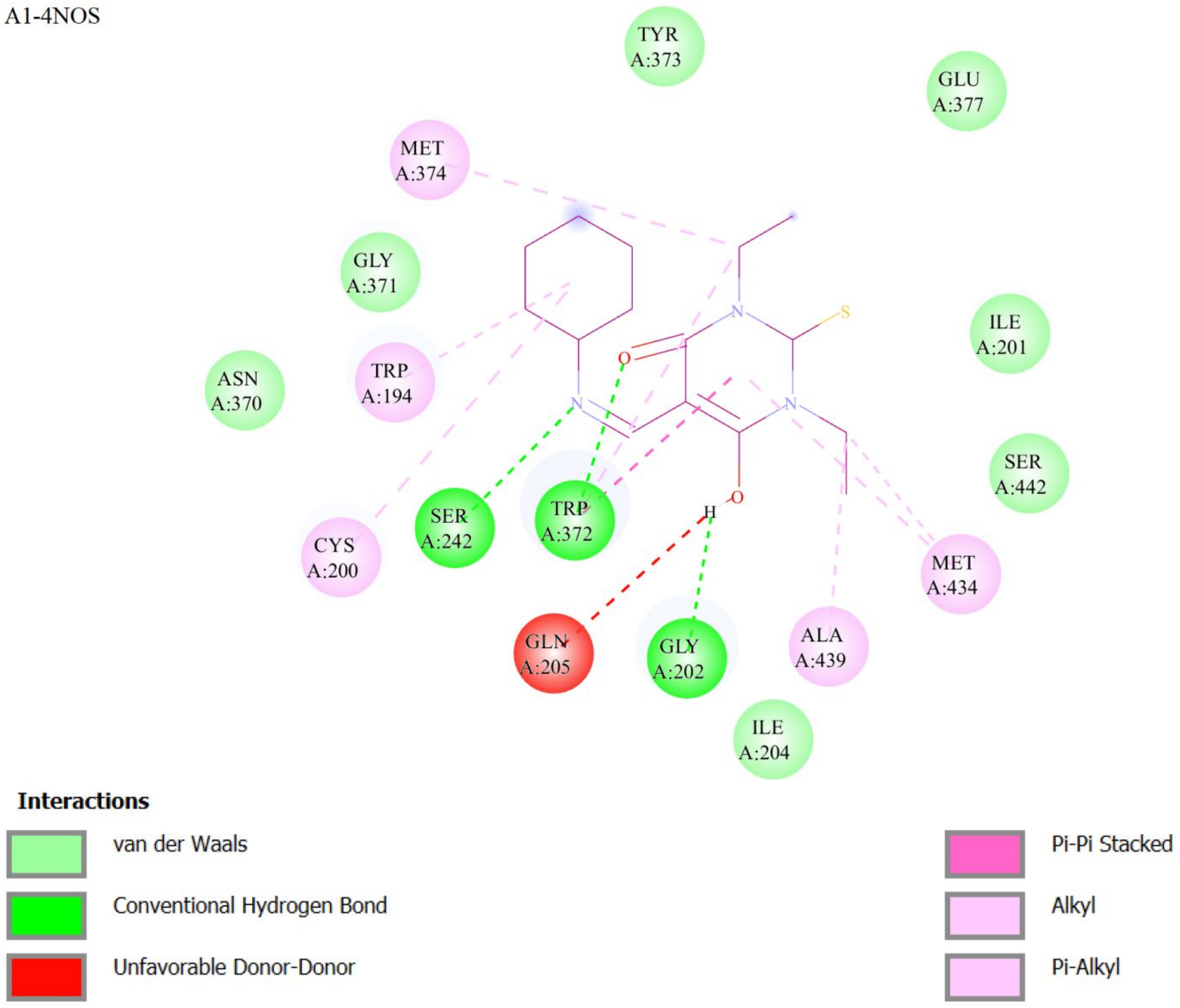


**Figure S40:** 2D diagram representing interactions of ligand molecule with the amino acid residues in the binding site of ligand-protein complex of compound A1 with nitric oxide synthase enzyme

A2-4NOS

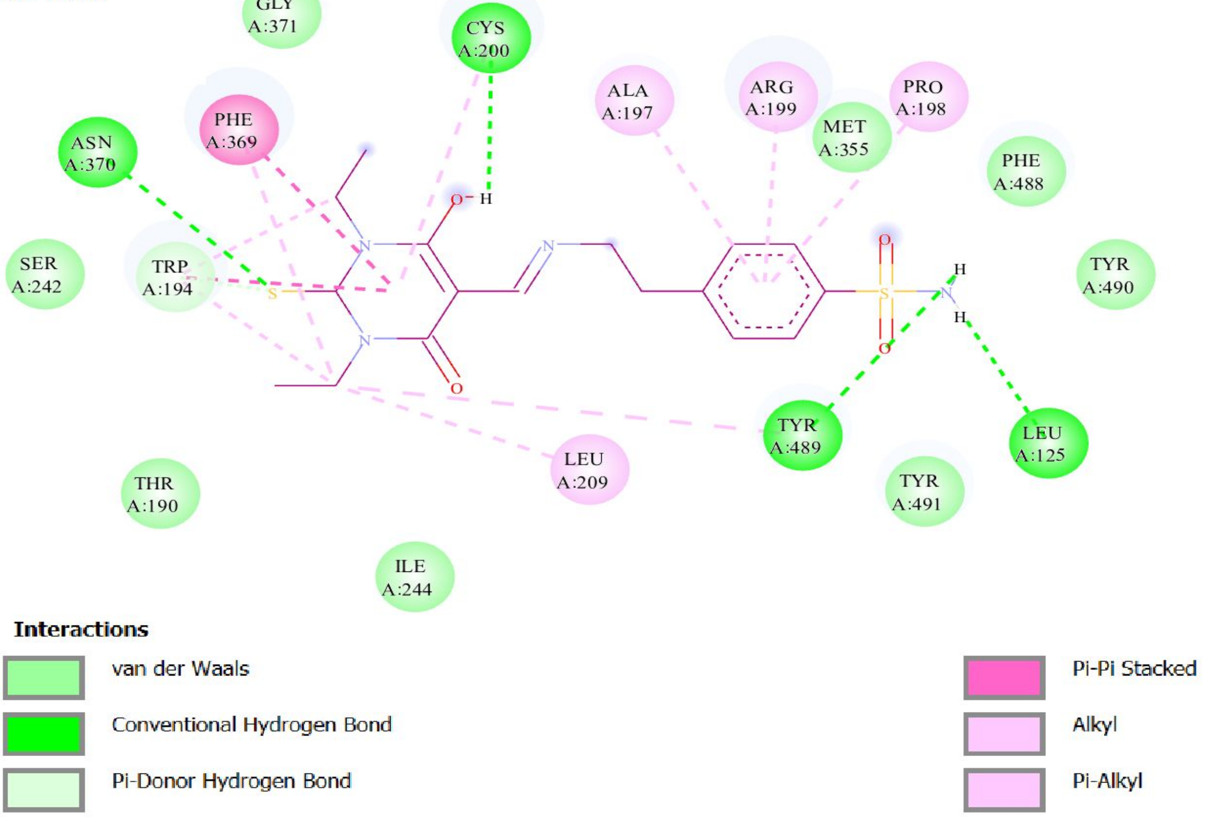


**Figure S41:** 2D diagram representing interactions of ligand molecule with the amino acid residues in the binding site of ligand-protein complex of compound A2 with nitric oxide synthase enzyme

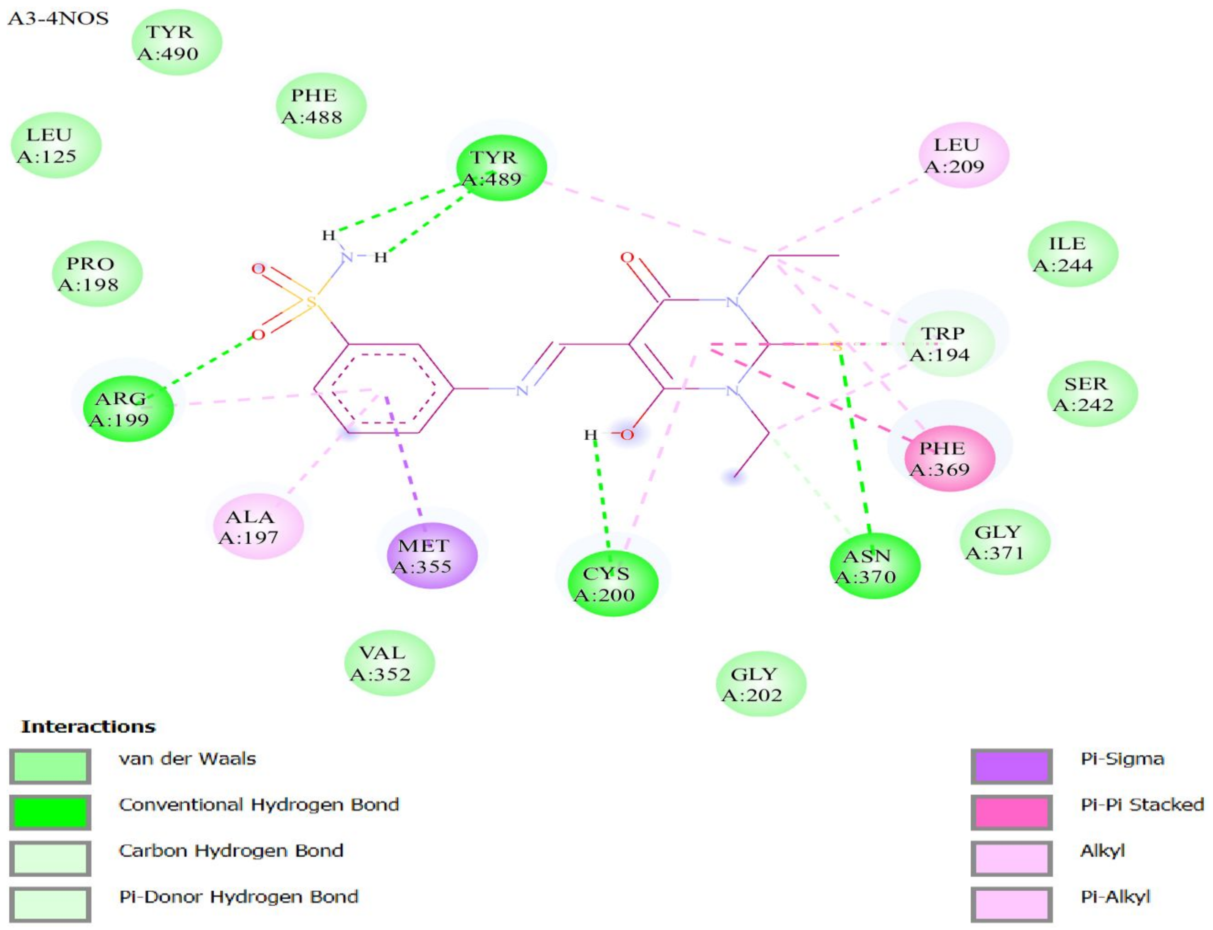


**Figure S42:** 2D diagram representing interactions of ligand molecule with the amino acid residues in the binding site of ligand-protein complex of compound A3 with nitric oxide synthase enzyme

A4-4NOS

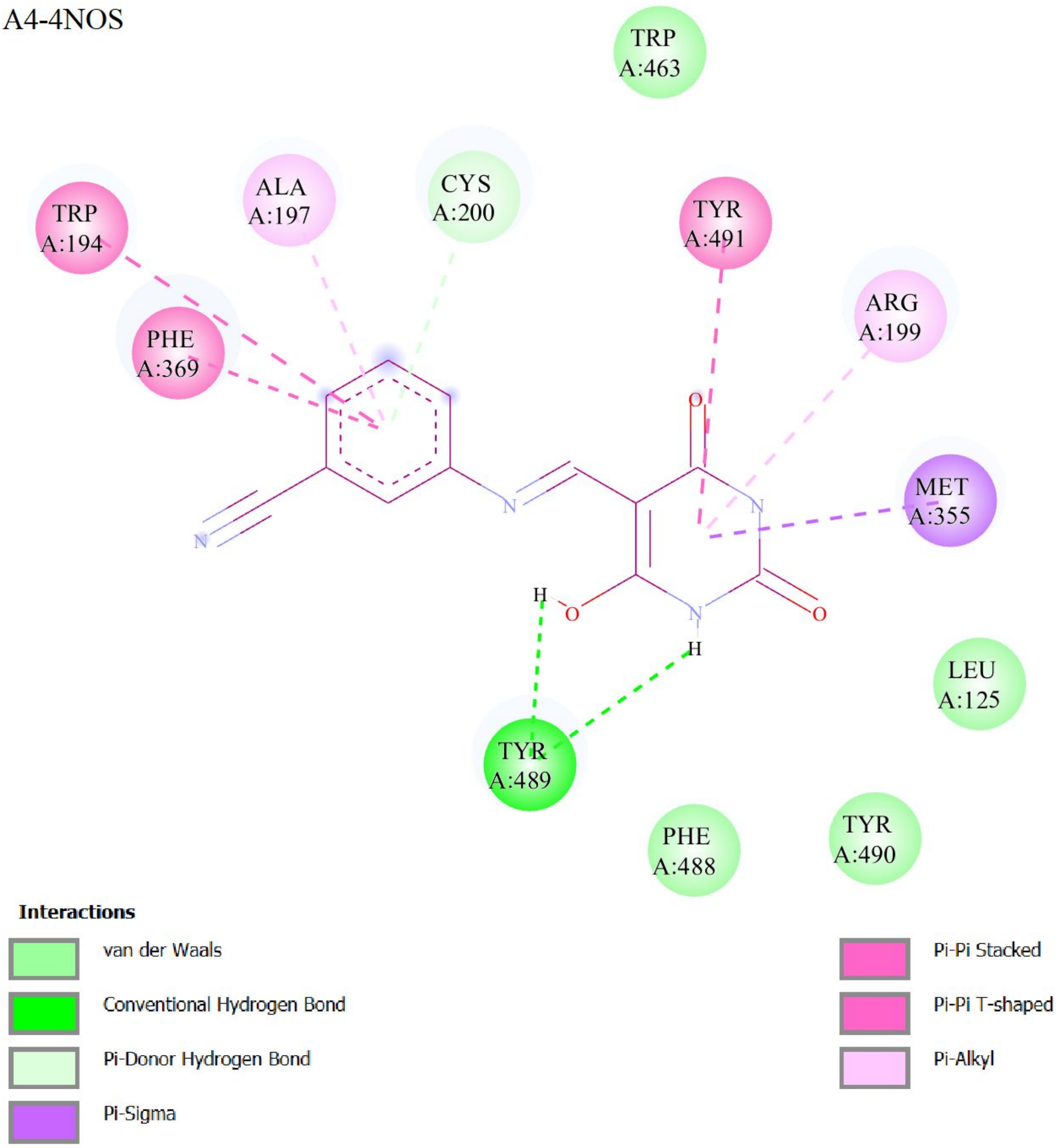


**Figure S43:** 2D diagram representing interactions of ligand molecule with the amino acid residues in the binding site of ligand-protein complex of compound A4 with nitric oxide synthase enzyme

A5-4NOS

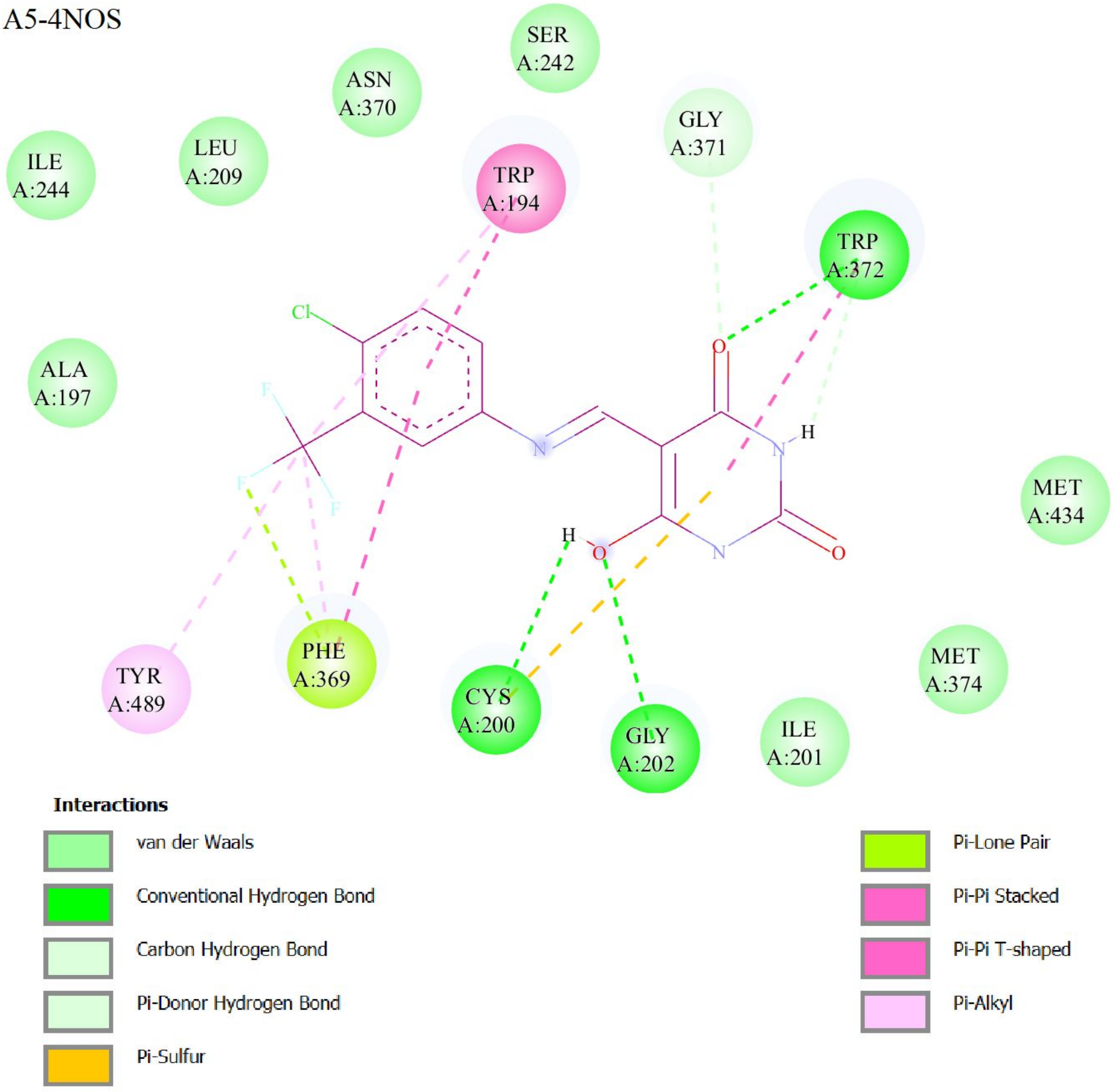


**Figure S44:** 2D diagram representing interactions of ligand molecule with the amino acid residues in the binding site of ligand-protein complex of compound A5 with nitric oxide synthase enzyme

A6-4NOS

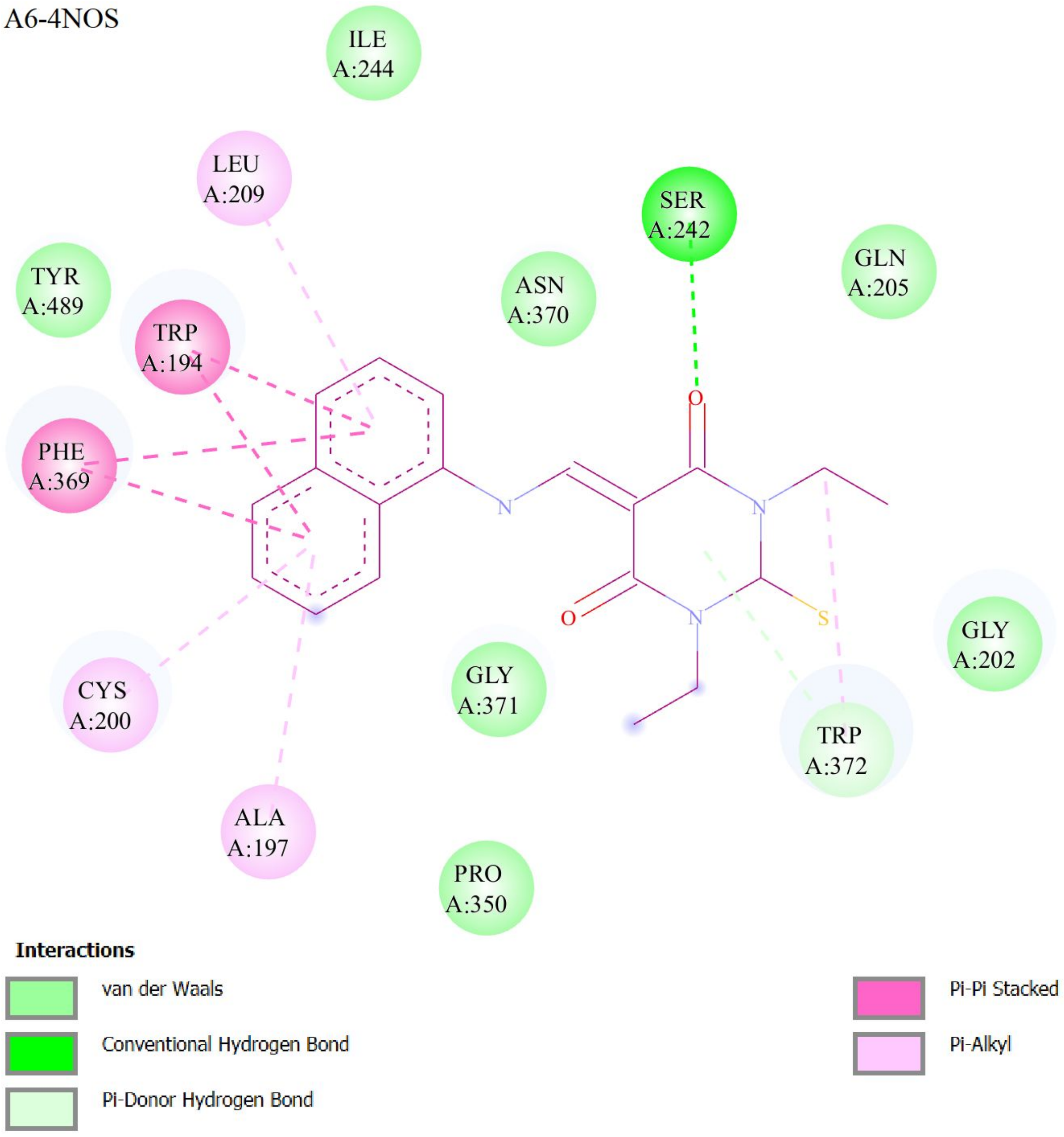


**Figure S45:** 2D diagram representing interactions of ligand molecule with the amino acid residues in the binding site of ligand-protein complex of compound A6 with nitric oxide synthase enzyme

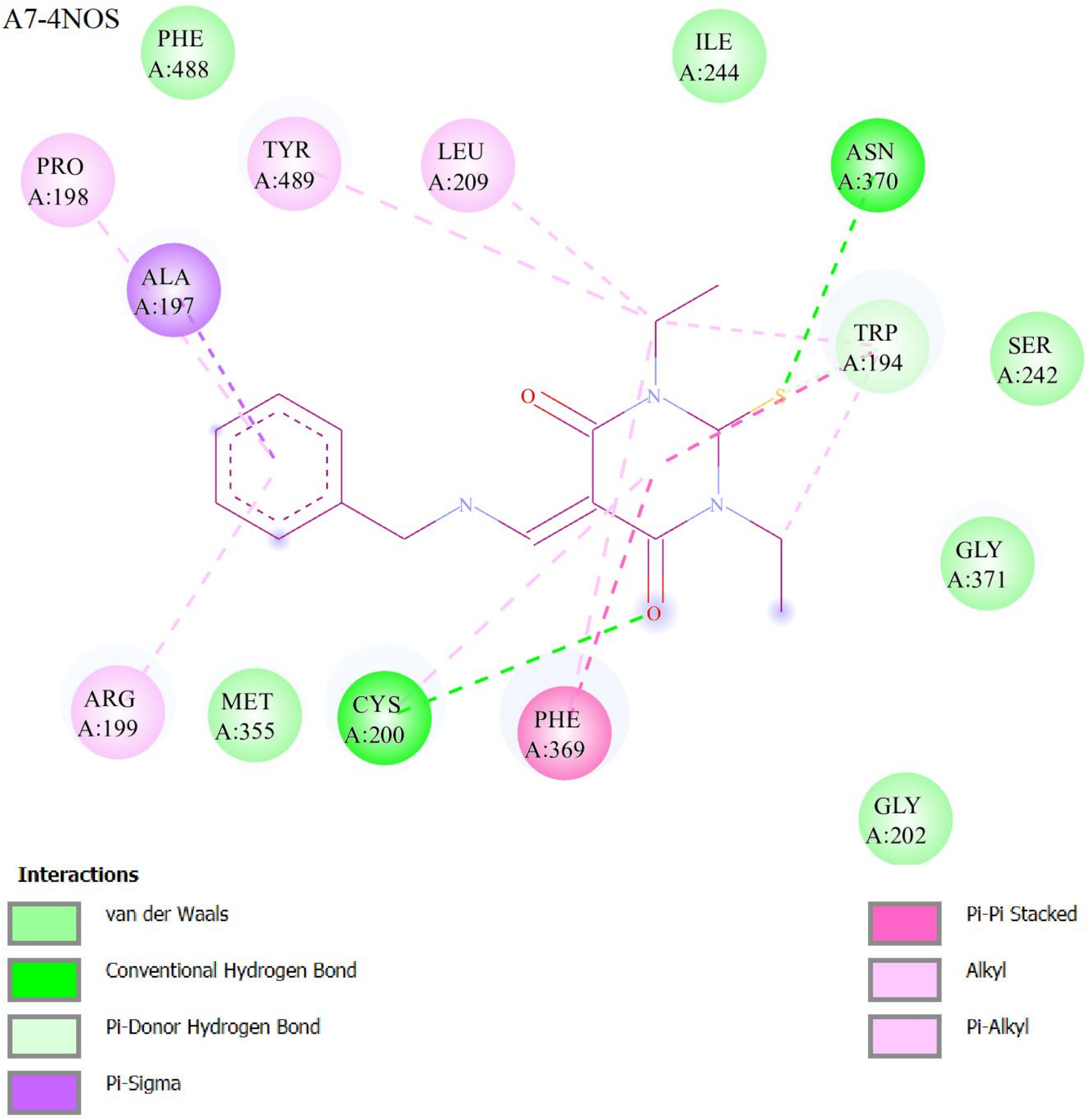


**Figure S46:** 2D diagram representing interactions of ligand molecule with the amino acid residues in the binding site of ligand-protein complex of compound A7 with nitric oxide synthase enzyme

A8-4NOS

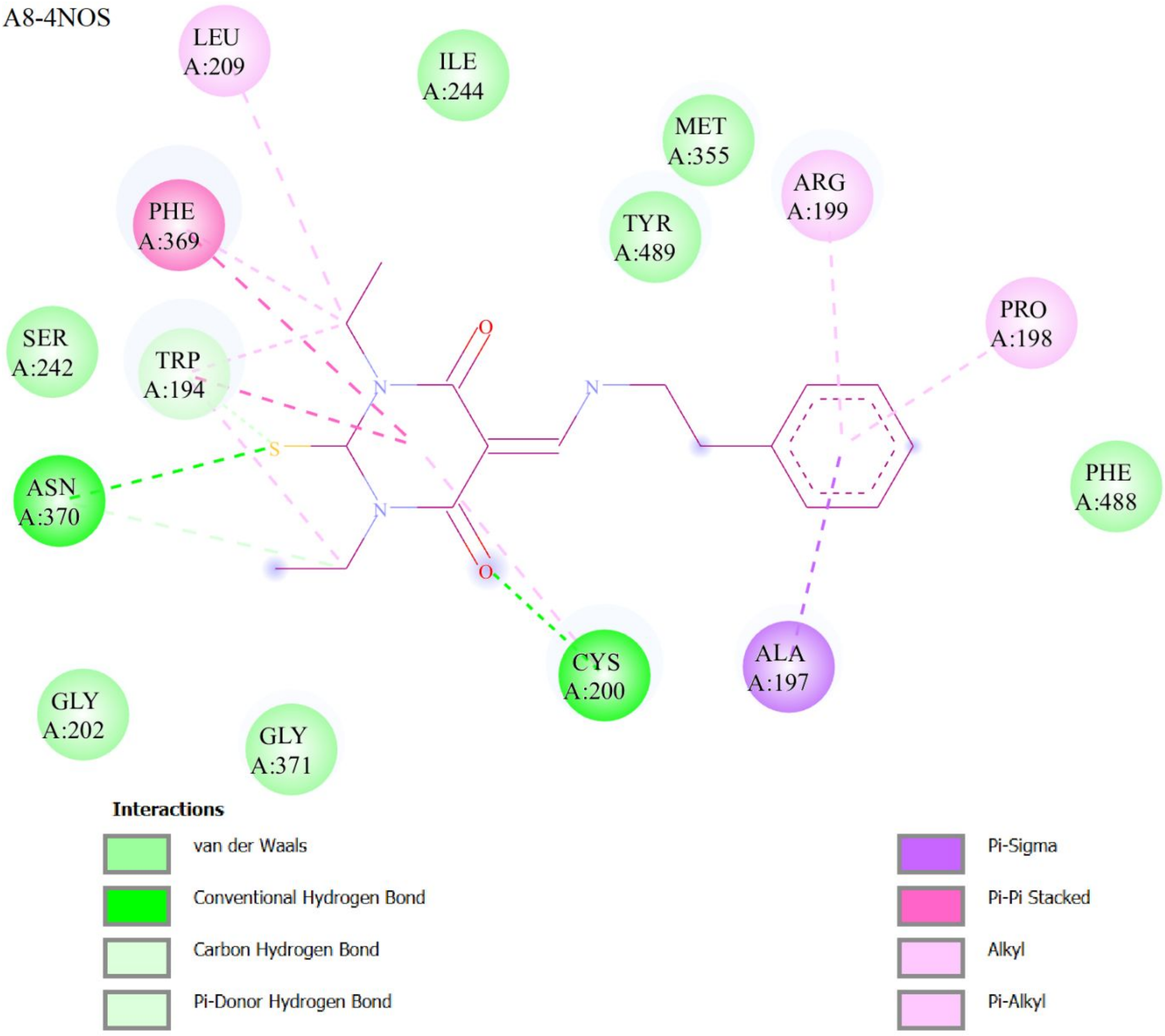


**Figure S47:** 2D diagram representing interactions of ligand molecule with the amino acid residues in the binding site of ligand-protein complex of compound A8 with nitric oxide synthase enzyme

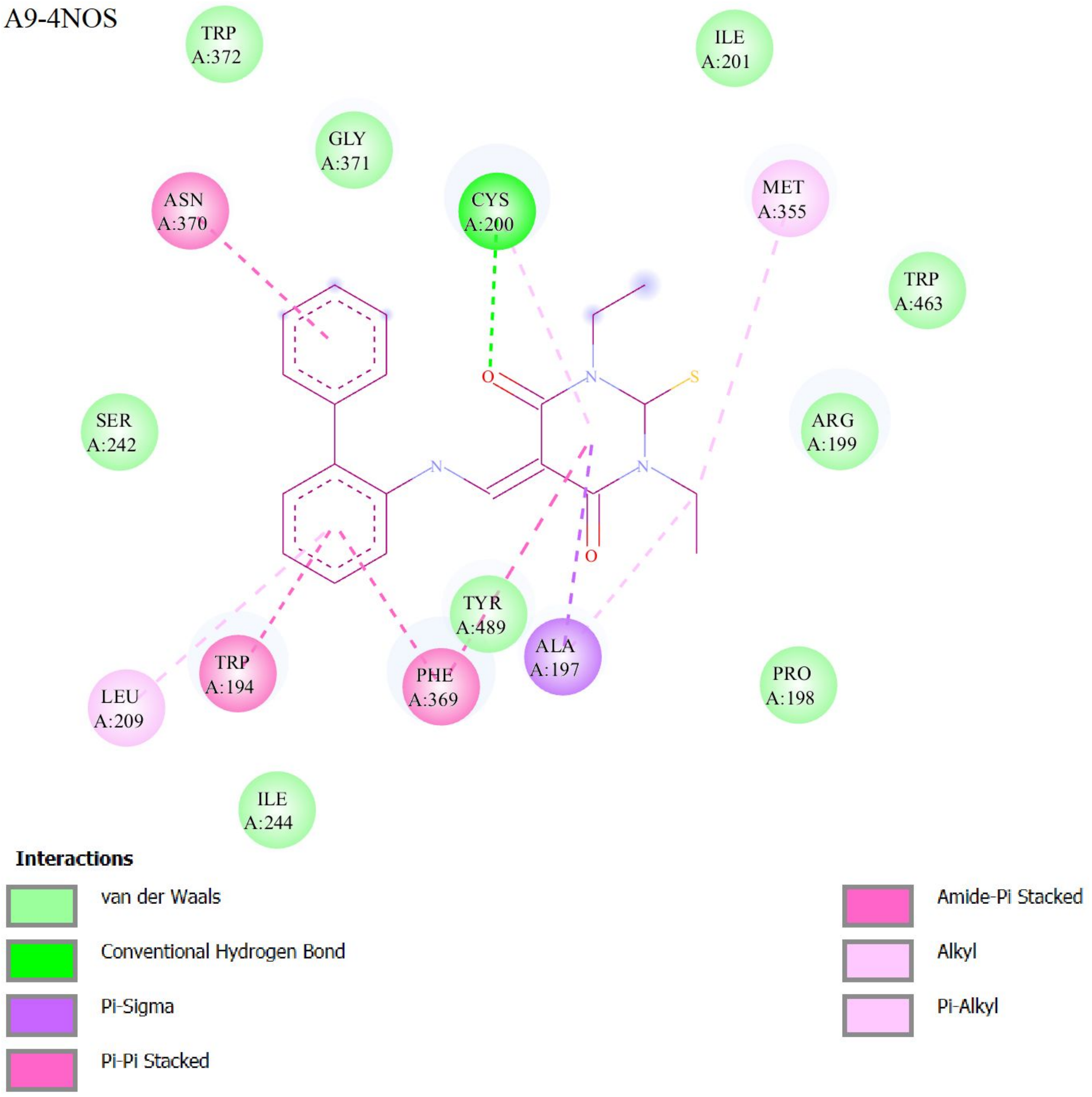


**Figure S48:** 2D diagram representing interactions of ligand molecule with the amino acid residues in the binding site of ligand-protein complex of compound A9 with nitric oxide synthase enzyme

A10-4NOS

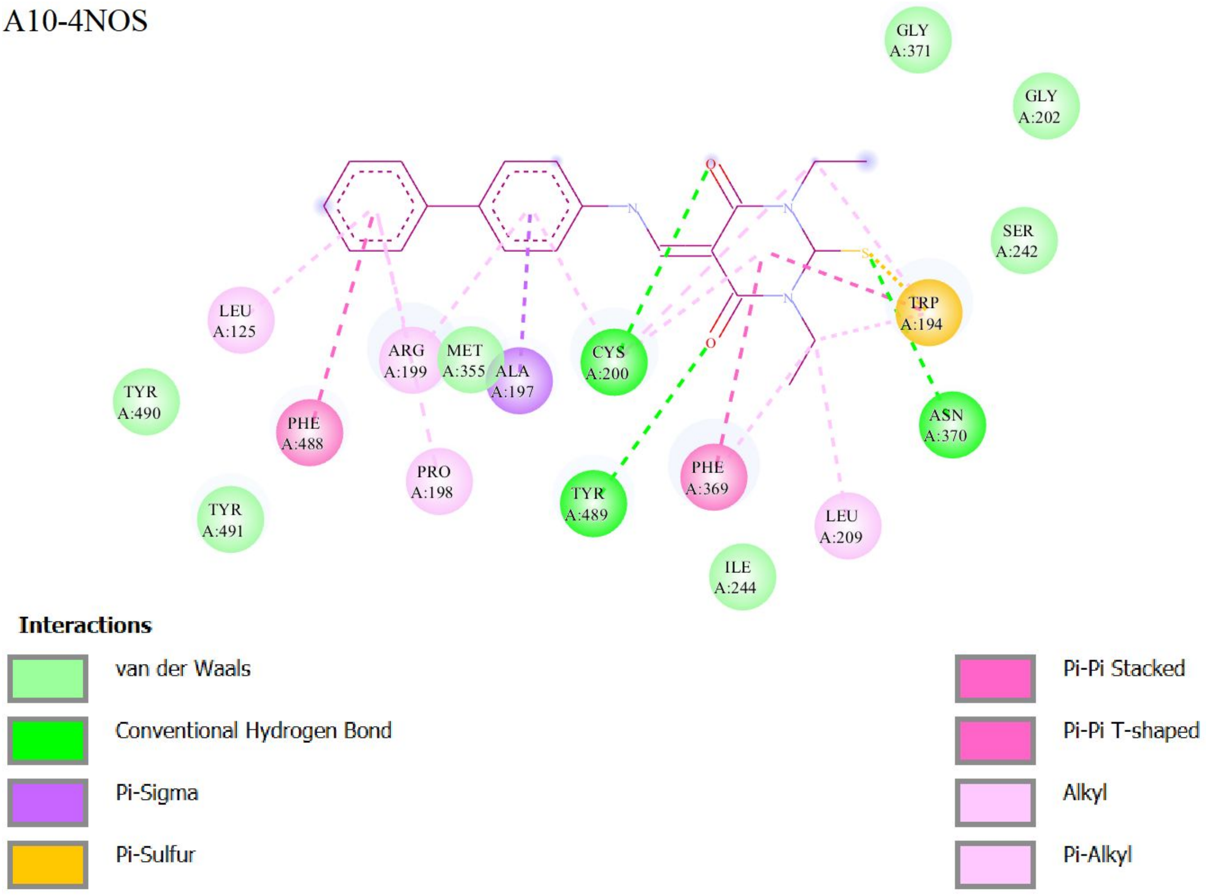


**Figure S49:** 2D diagram representing interactions of ligand molecule with the amino acid residues in the binding site of ligand-protein complex of compound A10 with nitric oxide synthase enzyme

A1-6Y84

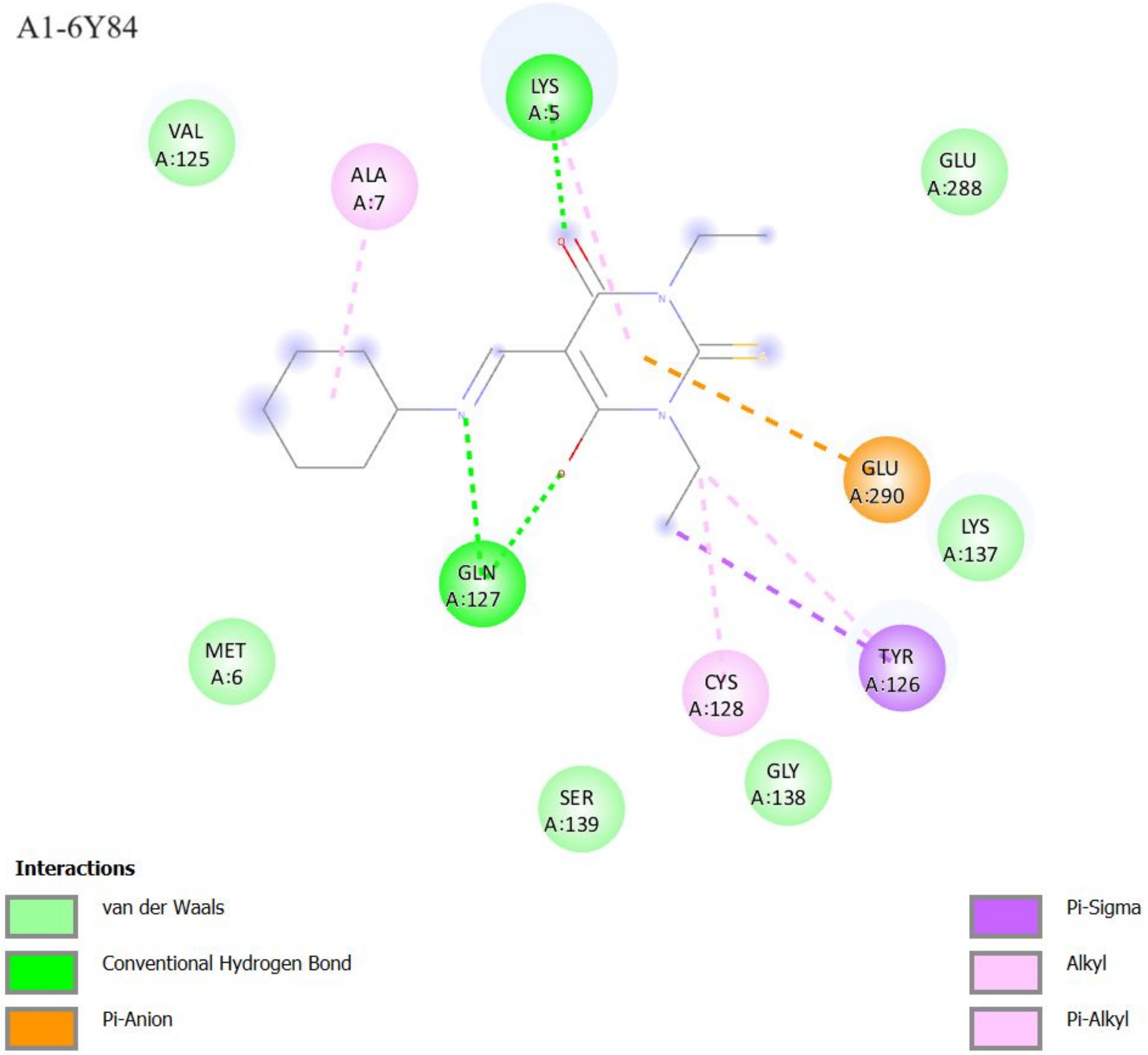


**Figure S50:** 2D diagram representing interactions of ligand molecule with the amino acid residues in the binding site of ligand-protein complex of compound A1 with SARS-CoV-2 protease enzyme

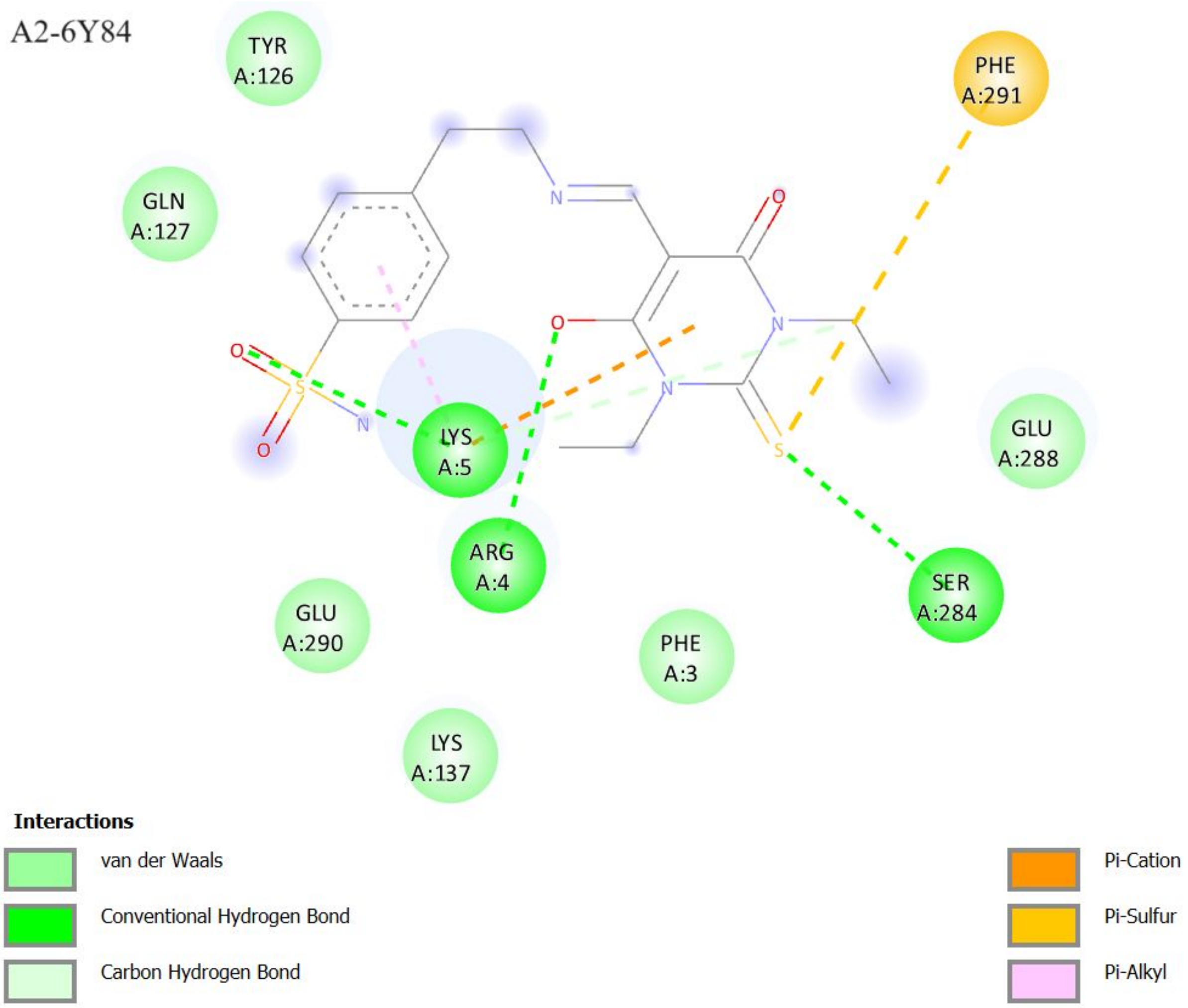


**Figure S51:** 2D diagram representing interactions of ligand molecule with the amino acid residues in the binding site of ligand-protein complex of compound A2 with SARS-CoV-2 protease enzyme

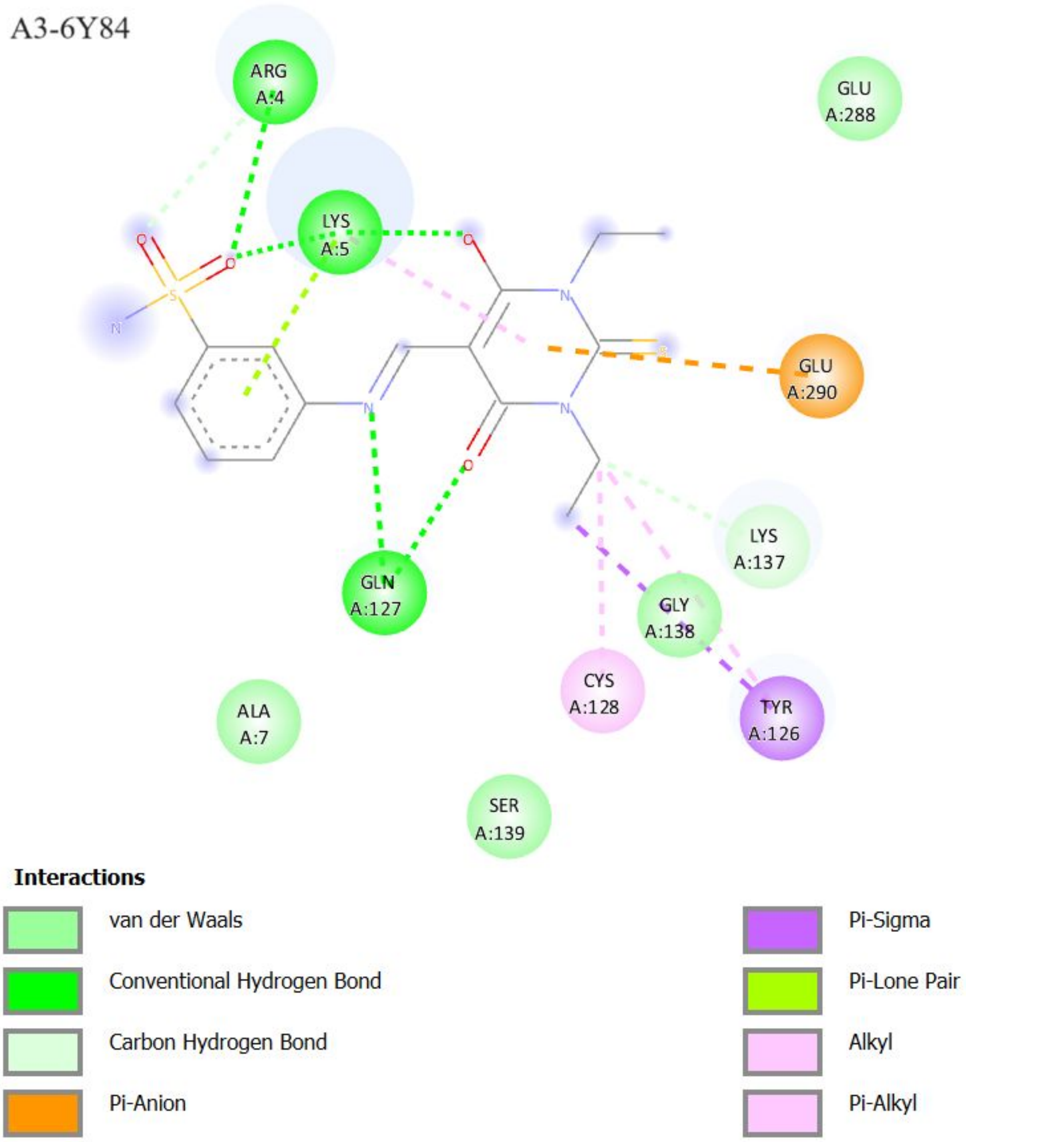


**Figure S52:** 2D diagram representing interactions of ligand molecule with the amino acid residues in the binding site of ligand-protein complex of compound A3 with SARS-CoV-2 protease enzyme

A4-6Y84

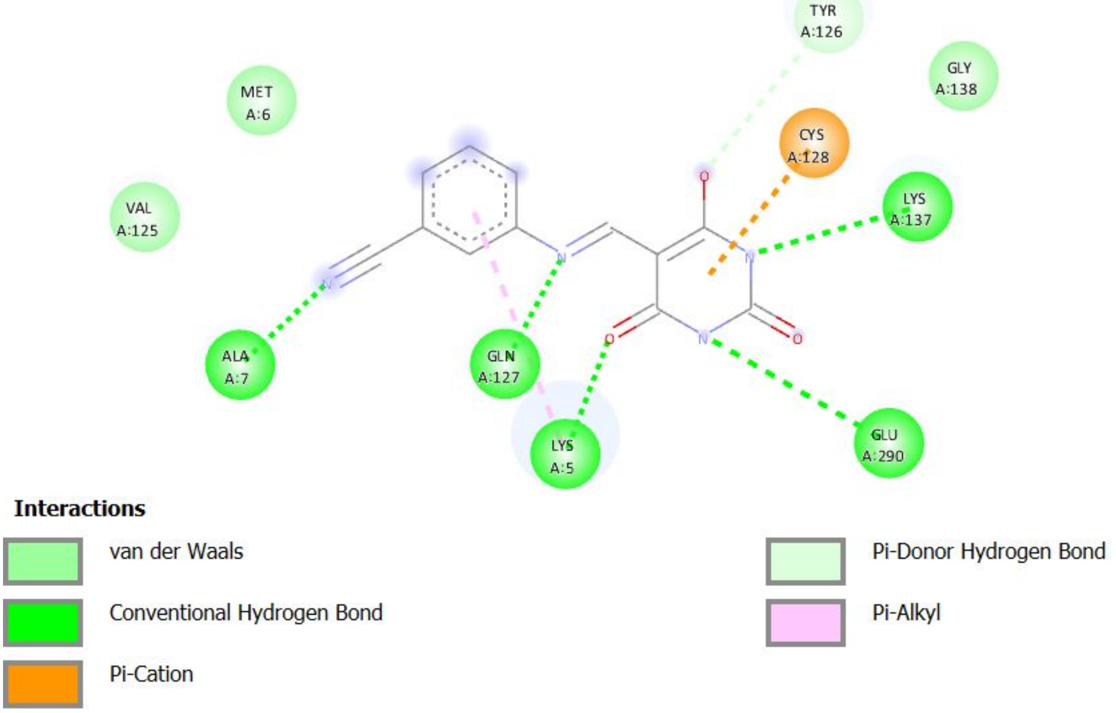


**Figure S53:** 2D diagram representing interactions of ligand molecule with the amino acid residues in the binding site of ligand-protein complex of compound A4 with SARS-CoV-2 protease enzyme

A5-6Y84

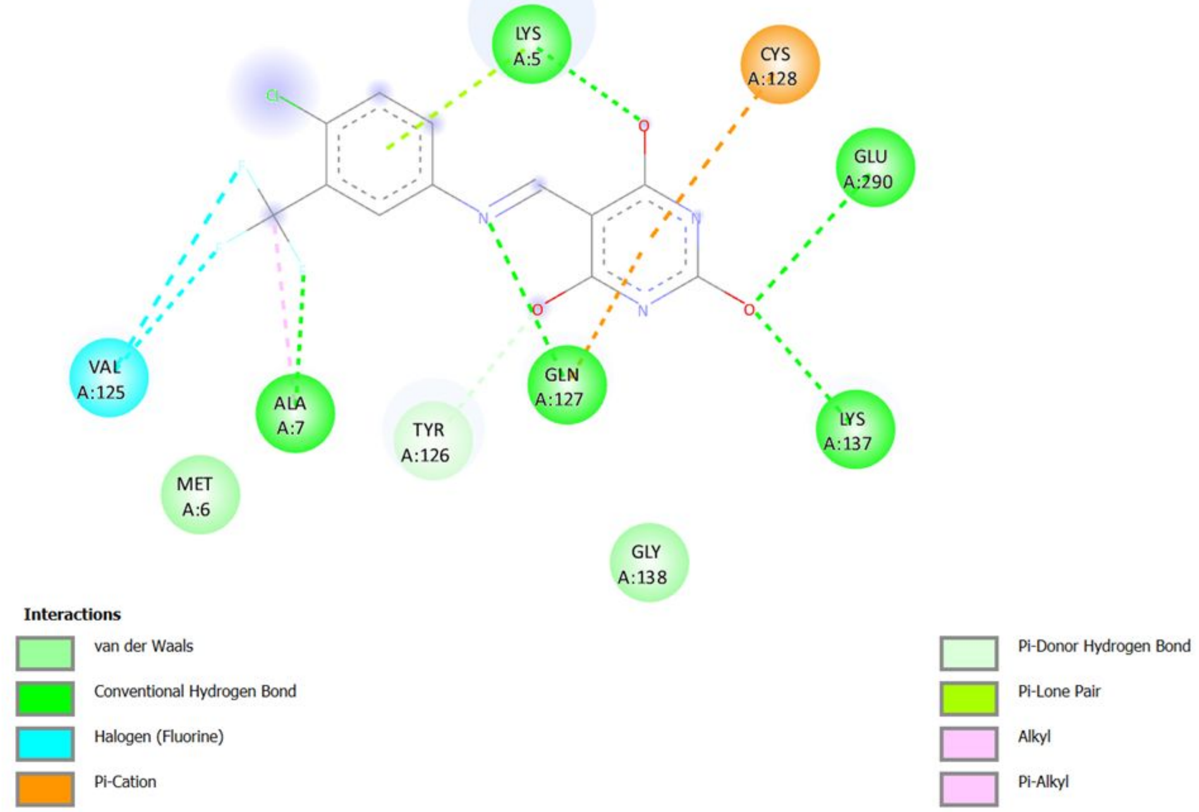


**Figure S54:** 2D diagram representing interactions of ligand molecule with the amino acid residues in the binding site of ligand-protein complex of compound A5 with SARS-CoV-2 protease enzyme

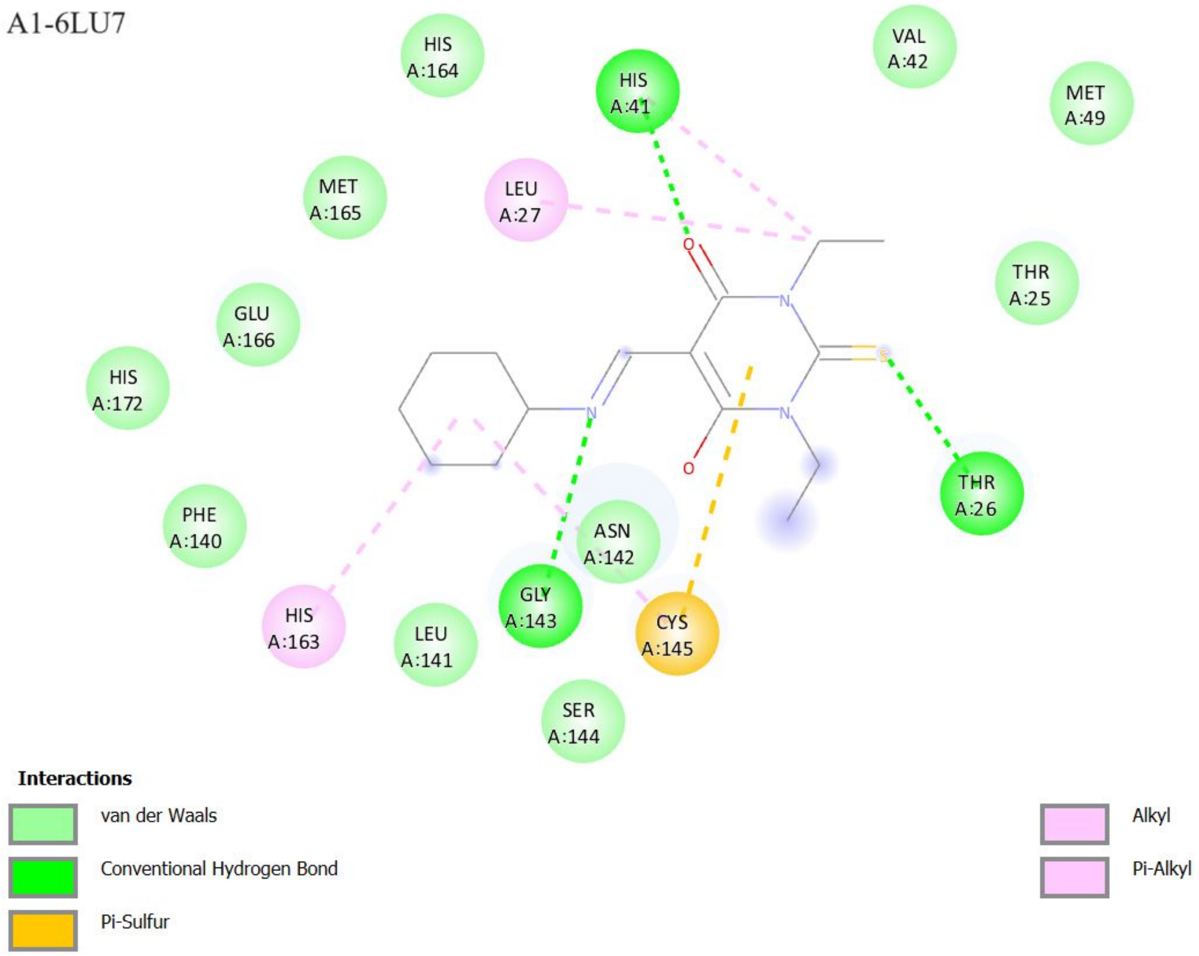


**Figure S55:** 2D diagram representing interactions of ligand molecule with the amino acid residues in the binding site of ligand-protein complex of compound A1 with COVID-19 main protease $M^{Pro}$ enzyme

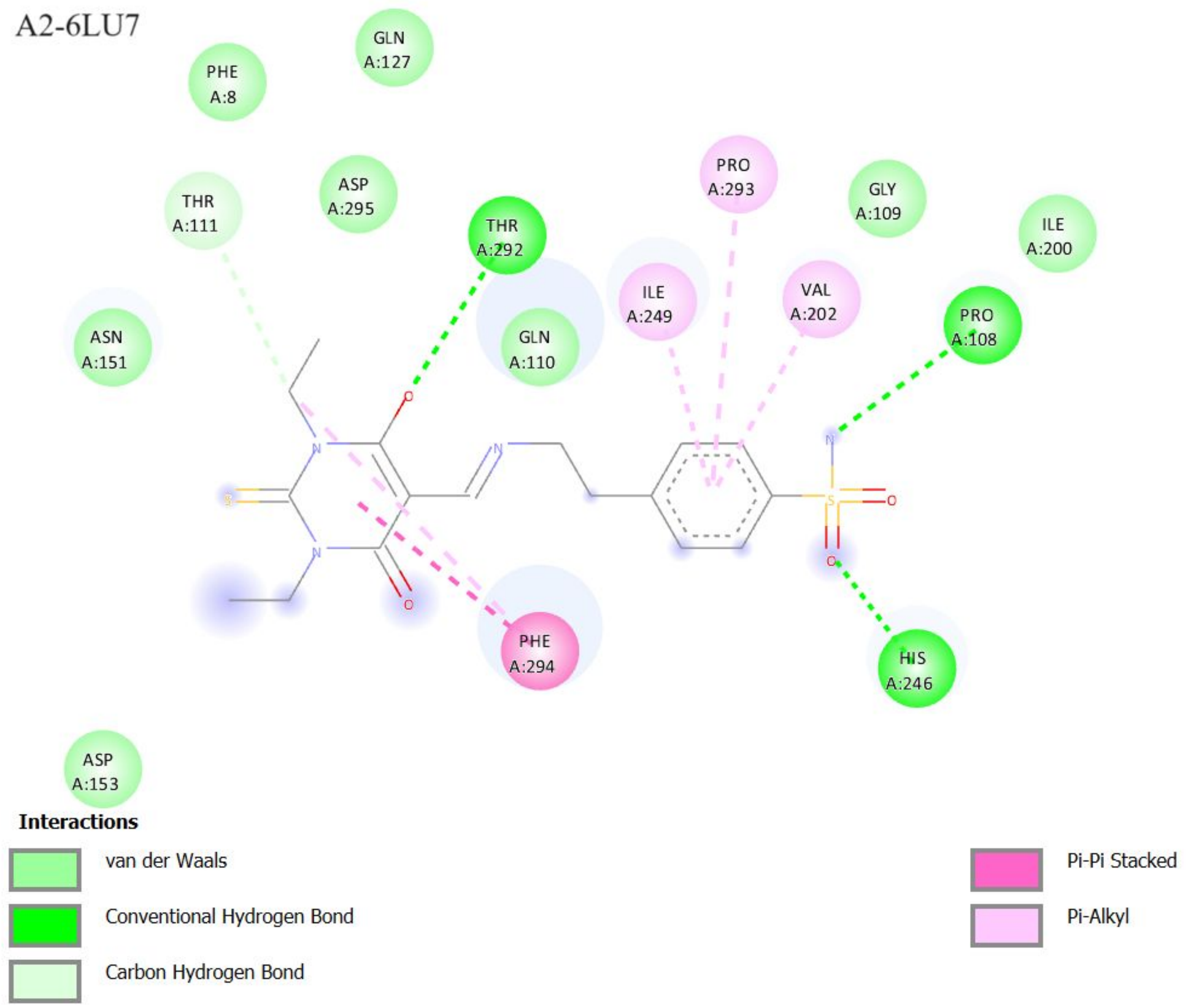


**Figure S56:** 2D diagram representing interactions of ligand molecule with the amino acid residues in the binding site of ligand-protein complex of compound A2 with COVID-19 main protease $M^{Pro}$ enzyme

A3-6LU7

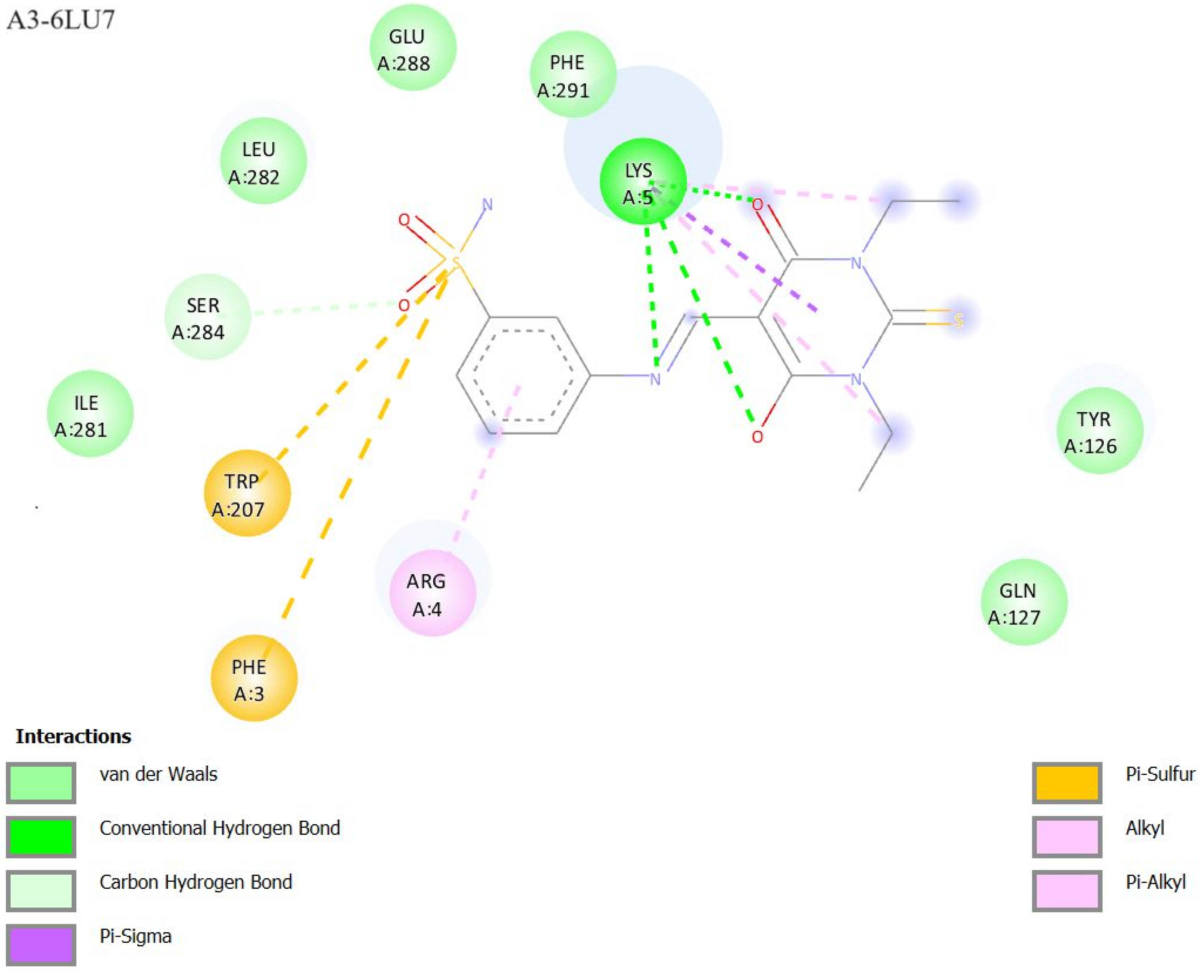


**Figure S57:** 2D diagram representing interactions of ligand molecule with the amino acid residues in the binding site of ligand-protein complex of compound A3 with COVID-19 main protease $M^{Pro}$ enzyme

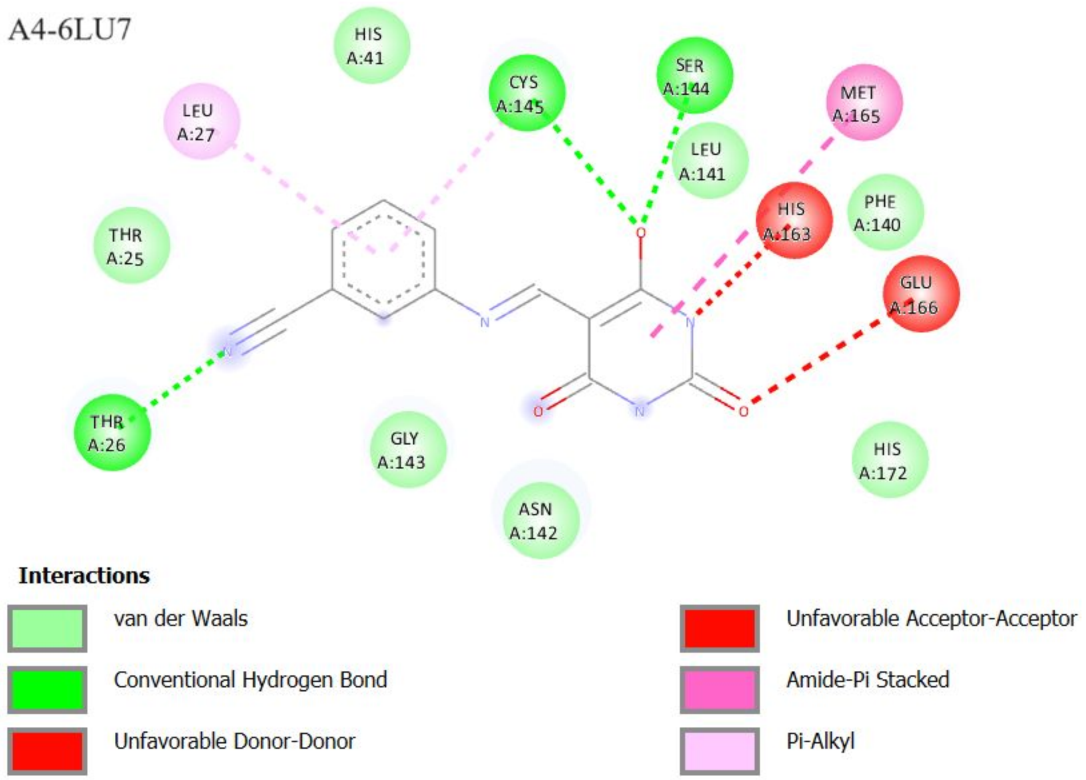


**Figure S58:** 2D diagram representing interactions of ligand molecule with the amino acid residues in the binding site of ligand-protein complex of compound A4 with COVID-19 main protease $M^{Pro}$ enzyme

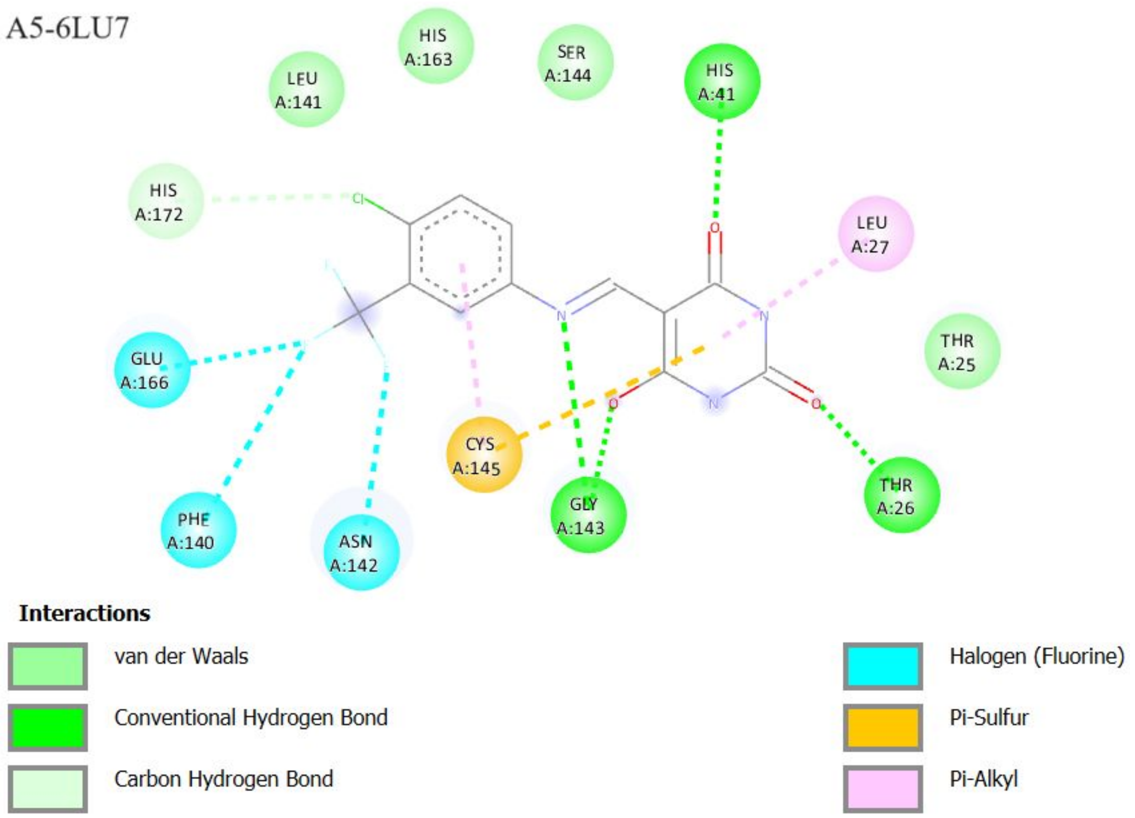


**Figure S59:** 2D diagram representing interactions of ligand molecule with the amino acid residues in the binding site of ligand-protein complex of compound A5 with COVID-19 main protease $M^{Pro}$ enzyme
